# A hybrid multiscale Monte Carlo algorithm (HyMSMC) to cope with disparity in time scales and species populations in intracellular networks

**DOI:** 10.1186/1471-2105-8-175

**Published:** 2007-05-24

**Authors:** Asawari Samant, Babatunde A Ogunnaike, Dionisios G Vlachos

**Affiliations:** 1 Department of Chemical Engineering, University of Delaware, Newark, Delaware, 19716. USA

## Abstract

**Background:**

The fundamental role that intrinsic stochasticity plays in cellular functions has been shown via numerous computational and experimental studies. In the face of such evidence, it is important that intracellular networks are simulated with stochastic algorithms that can capture molecular fluctuations. However, separation of time scales and disparity in species population, two common features of intracellular networks, make stochastic simulation of such networks computationally prohibitive. While recent work has addressed each of these challenges separately, a generic algorithm that can *simultaneously *tackle disparity in time scales *and *population scales in stochastic systems is currently lacking. In this paper, we propose the hybrid, multiscale Monte Carlo (HyMSMC) method that fills in this void.

**Results:**

The proposed HyMSMC method blends stochastic singular perturbation concepts, to deal with potential stiffness, with a hybrid of exact and coarse-grained stochastic algorithms, to cope with separation in population sizes. In addition, we introduce the computational singular perturbation (CSP) method as a means of systematically partitioning fast and slow networks and computing relaxation times for convergence. We also propose a new criteria of convergence of fast networks to stochastic low-dimensional manifolds, which further accelerates the algorithm.

**Conclusion:**

We use several prototype and biological examples, including a gene expression model displaying bistability, to demonstrate the efficiency, accuracy and applicability of the HyMSMC method. Bistable models serve as stringent tests for the success of multiscale MC methods and illustrate limitations of some literature methods.

## Background

Stochastic behavior, resulting from the small population of species in an intracellular medium, can stimulate interesting molecular phenomena, such as noise-induced bifurcations, phenotypic heterogeneity, etc. Through such phenomena, intracellular noise can have an impact on the physiology of the cell [1,2]. It is therefore important that *in silico *analysis of intracellular networks, aimed at relating cell function to molecular events, is capable of capturing fluctuations. Exact stochastic algorithms, such as the stochastic simulation algorithm (SSA) [3,4] and its variants [5], can capture the molecular noise by accounting for the random nature of biochemical processes.

The SSA [3,4] provides the exact solution but can be computationally intensive, especially when large populations and/or reaction networks are involved. τ-leap methods [6-9], which accelerate the simulation by firing multiple reactions in a coarse time step, provide an excellent alternative when large populations are involved. However, the original Poisson-based τ-leap method [6] and its modifications, the binomial τ-leap [7,8] and the implicit τ-leap [10] methods, do not perform too well at low populations. Therefore, an intracellular network comprised of mixed population scales is not amenable to a τ-leap treatment. A modified τ-leap algorithm [11] was recently developed to avoid negative populations that can occur in the Poisson τ-leap method. Implicitly, this modification results in a hybrid, stochastic algorithm that handles mixed population levels by seamlessly switching between the SSA for low population species and the Poisson τ-leap method for large population species [11]. Similarly, the partitioned leaping method [12] combines the exact next reaction method [5] with the Poisson τ-leap [6] method to enable stochastic simulations over a disparate range of populations.

Another aspect of biological networks plaguing stochastic simulation is the presence of processes with disparate reaction rates. Fast reactions, which are sampled more frequently by the SSA, reduce the size of the time step. As a result, it is difficult to simulate macroscopic real-time. τ-leap methods are also inefficient when a large separation of time scales is encountered. Most multiscale, stochastic algorithms [13-21] accelerate simulation of stiff networks by reducing the time spent in simulating the fast network. One way to achieve this is to use an approximate, accelerated algorithm, such as the Langevin method [22], to speed up the simulation of fast reactions [15,19]. This approach tacitly assumes that the faster kinetics arise purely from the large populations, and is not applicable to cases involving fast reactions with small populations. An alternative multiscale approach [13,16-18,20] based on the slow-scale SSA (SS-SSA) [13,14] concept, uses information from the quasi-equilibrium (QE) description of the fast network to evolve the slow network (see Appendix A). In this multiscale approach, individual networks are modeled with the SSA, and thus, large populations in either the fast or the slow network cannot be handled effectively.

**Figure 1:**
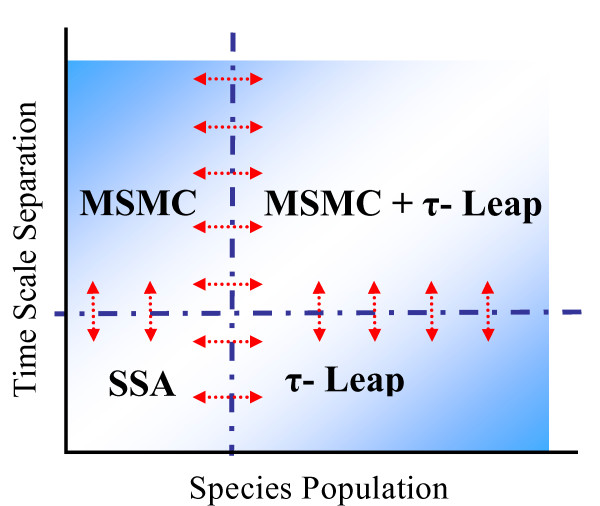
Schematic showing various stochastic algorithms of the HyMSMC method, depending on the scales in the network. The arrows represent a transition between algorithms.

**Figure 2:**
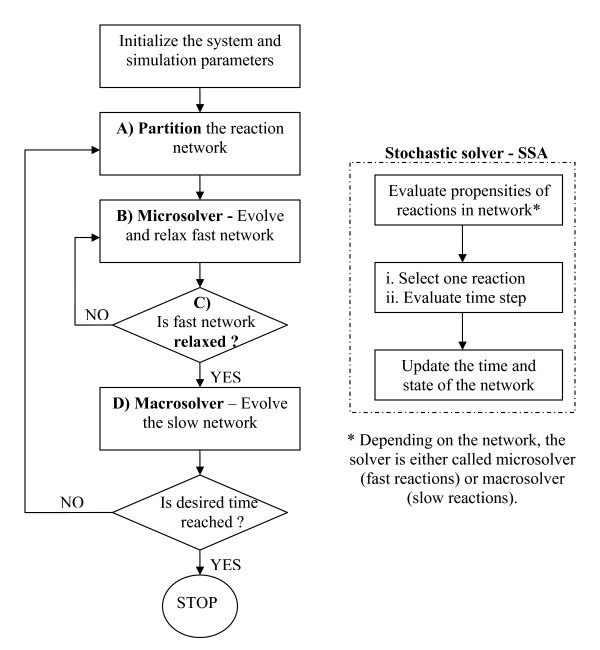
Flowchart illustrating the use of the quasi-equilibrium (QE) approximation in the MSMC or HyMSMC framework (left) and the SSA (right).

A generic stochastic algorithm, which simultaneously addresses the disparity in time scales and species populations in well-mixed reaction networks, is currently lacking (see Figure 1). In this paper, we propose a hybrid, multiscale Monte Carlo (abbreviated as HyMSMC) algorithm to fill in this gap (a flow chart of the steps involved is presented in (Figure 2). Like our original multiscale Monte Carlo (MSMC) algorithm [17], the proposed HyMSMC algorithm makes no *a priori *assumptions about the scales and collapses to the exact SSA and/or non-multiscale stochastic solver, when a scale separation is absent. Additional new elements in our work include: (1) the introduction of the *computational singular perturbation (CSP) *framework, as an auxiliary tool, to aid network partitioning and determine relaxation times of the fast network for its convergence and (2) of a *new statistical relaxation criterion *that eliminates the need for the complete description of the QE probability distribution function (PDF), and (3) the applicability of the HyMSMC method to networks displaying bistability in the fast or the slow networks. To the best of our knowledge, this is the first successful application of a multiscale, stochastic algorithm to a bistable network, reported in the literature.

The paper is organized as follows. We begin with a brief introduction of the SSA notation, and then provide a detailed explanation of the CSP-assisted partitioning technique and the new statistical relaxation criterion. Next, we introduce the new hybrid multiscale Monte Carlo (HyMSMC) algorithm. Finally, we demonstrate the efficiency, accuracy and generality of the new algorithm with a few prototype and real biological examples.

## Results and Discussion

### Stochastic simulation algorithm (SSA) Notation

The notation we follow is identical to that of Gillespie [3,4]. Consider a well-mixed, isothermal system of n species ***S ***≡ {S_1_, S_2_, ..., S_n_} reacting through m reactions ***R ***≡ {R_1_, R_2_, ..., R_m_}. Let the state of the system at any time t be denoted by a n-dimensional vector, **X**(t) = (X_1_(t), X_2_(t), ..., X_n_(t)), where X_i_(t) is the number of molecules of species S_i _at time t. Let the n-dimensional vector **υ**_**j **_correspond to the stoichiometry vector of reaction **R**_j_, such that υ_ij _is the stoichiometric coefficient of species S_i _in reaction R_j_. Given that the system is in a state **X**(t) = **x **at time t, we define a propensity function, a_j_(**x**, t) such that a_j_(**x**,t)dt gives the transition probability of the j^th ^reaction, Rj, occurring in an infinitesimally small time interval (t, t+dt). This function is typically dependent on the state of the species and the reaction conditions of the network, such as temperature and pressure.

### Stochastic Quasi-Equilibrium, Network Partitioning, Relaxation, and Evolution

#### a. Quasi-equilibrium (QE) in stochastic systems

In dealing with numerical "stiffness" in stochastic systems, arising from separation of time scales, one needs to account for the probabilistic nature of the QE [23], which is given by a distribution of states, rather than a single state. Accordingly, every state of the slow variables determines a QE PDF of the fast network, i.e., a stochastic low-dimensional manifold. Stochastic QE is defined by a time-invariant QE PDF, i.e., the existence of a stable equilibrium of the fast network [13]

lim⁡t′→∞P^(xf,t′|x,t)=P∞(xf|x),
 MathType@MTEF@5@5@+=feaafiart1ev1aaatCvAUfKttLearuWrP9MDH5MBPbIqV92AaeXatLxBI9gBaebbnrfifHhDYfgasaacH8akY=wiFfYdH8Gipec8Eeeu0xXdbba9frFj0=OqFfea0dXdd9vqai=hGuQ8kuc9pgc9s8qqaq=dirpe0xb9q8qiLsFr0=vr0=vr0dc8meaabaqaciaacaGaaeqabaqabeGadaaakeaadaWfqaqaaiGbcYgaSjabcMgaPjabc2gaTbWcbaacbaGaf8hDaqNbauaacqGHsgIRcqGHEisPaeqaaOGaf8huaaLbaKaacqGGOaakieqacqGF4baEdaahaaWcbeqaaiab+zgaMbaakiabcYcaSiqb=rha0zaafaGaeiiFaWNae4hEaGNaeiilaWIae8hDaqNaeiykaKIaeyypa0Jae8huaa1aaSbaaSqaaiabg6HiLcqabaGccqGGOaakcqGF4baEdaahaaWcbeqaaiab+zgaMbaakiabcYha8jab+Hha4jabcMcaPiabcYcaSaaa@4FC6@

where P^
 MathType@MTEF@5@5@+=feaafiart1ev1aaatCvAUfKttLearuWrP9MDH5MBPbIqV92AaeXatLxBI9gBaebbnrfifHhDYfgasaacH8akY=wiFfYdH8Gipec8Eeeu0xXdbba9frFj0=OqFfea0dXdd9vqai=hGuQ8kuc9pgc9s8qqaq=dirpe0xb9q8qiLsFr0=vr0=vr0dc8meaabaqaciaacaGaaeqabaqabeGadaaakeaaieaacuWFqbaugaqcaaaa@2DEA@(**x**^f^, t'|**x**, t) is the probability of observing X^f
 MathType@MTEF@5@5@+=feaafiart1ev1aaatCvAUfKttLearuWrP9MDH5MBPbIqV92AaeXatLxBI9gBaebbnrfifHhDYfgasaacH8akY=wiFfYdH8Gipec8Eeeu0xXdbba9frFj0=OqFfea0dXdd9vqai=hGuQ8kuc9pgc9s8qqaq=dirpe0xb9q8qiLsFr0=vr0=vr0dc8meaabaqaciaacaGaaeqabaqabeGadaaakeaadaqiaaqaaGqabiab=HfaybGaayPadaWaaWbaaSqabeaaieaacqGFMbGzaaaaaa@3033@(t') = **x**^f^, given that the system is in a state **x **at time t. X^f
 MathType@MTEF@5@5@+=feaafiart1ev1aaatCvAUfKttLearuWrP9MDH5MBPbIqV92AaeXatLxBI9gBaebbnrfifHhDYfgasaacH8akY=wiFfYdH8Gipec8Eeeu0xXdbba9frFj0=OqFfea0dXdd9vqai=hGuQ8kuc9pgc9s8qqaq=dirpe0xb9q8qiLsFr0=vr0=vr0dc8meaabaqaciaacaGaaeqabaqabeGadaaakeaadaqiaaqaaGqabiab=HfaybGaayPadaWaaWbaaSqabeaaieaacqGFMbGzaaaaaa@3033@(t) is a stochastic process that is identical to **X**^f^(t), but describes the evolution of the fast species purely via the fast network.

The fast species relax to the stationary PDF, P_∞_(**x**^**f**^|**x**), at t'→∞ (asymptotic limit); in practice, this happens over a relaxation time, τRelf
 MathType@MTEF@5@5@+=feaafiart1ev1aaatCvAUfKttLearuWrP9MDH5MBPbIqV92AaeXatLxBI9gBaebbnrfifHhDYfgasaacH8akY=wiFfYdH8Gipec8Eeeu0xXdbba9frFj0=OqFfea0dXdd9vqai=hGuQ8kuc9pgc9s8qqaq=dirpe0xb9q8qiLsFr0=vr0=vr0dc8meaabaqaciaacaGaaeqabaqabeGadaaakeaaiiGacqWFepaDdaqhaaWcbaacbaGae4NuaiLae4xzauMae4hBaWgabaGae4Nzaygaaaaa@33D0@, of the fast network. Accuracy of the QE approximation requires that the relaxation time, τRelf
 MathType@MTEF@5@5@+=feaafiart1ev1aaatCvAUfKttLearuWrP9MDH5MBPbIqV92AaeXatLxBI9gBaebbnrfifHhDYfgasaacH8akY=wiFfYdH8Gipec8Eeeu0xXdbba9frFj0=OqFfea0dXdd9vqai=hGuQ8kuc9pgc9s8qqaq=dirpe0xb9q8qiLsFr0=vr0=vr0dc8meaabaqaciaacaGaaeqabaqabeGadaaakeaaiiGacqWFepaDdaqhaaWcbaacbaGae4NuaiLae4xzauMae4hBaWgabaGae4Nzaygaaaaa@33D0@, be much smaller than the smallest time scale, min⁡j∈Rs(τjs)
 MathType@MTEF@5@5@+=feaafiart1ev1aaatCvAUfKttLearuWrP9MDH5MBPbIqV92AaeXatLxBI9gBaebbnrfifHhDYfgasaacH8akY=wiFfYdH8Gipec8Eeeu0xXdbba9frFj0=OqFfea0dXdd9vqai=hGuQ8kuc9pgc9s8qqaq=dirpe0xb9q8qiLsFr0=vr0=vr0dc8meaabaqaciaacaGaaeqabaqabeGadaaakeaadaWfqaqaaiGbc2gaTjabcMgaPjabc6gaUbWcbaacbaGae8NAaOMaeyicI4mcbmGae4Nuai1aaWbaaWqabeaaieGacqqFZbWCaaaaleqaaOGaeiikaGccciGaeWhXdq3aa0baaSqaaiab=PgaQbqaaiab=nhaZbaakiabcMcaPaaa@3D4E@, of the slow network

lim⁡t′→τRelfP^(xf,t′|x,t)≈P∞(xf|x).
 MathType@MTEF@5@5@+=feaafiart1ev1aaatCvAUfKttLearuWrP9MDH5MBPbIqV92AaeXatLxBI9gBaebbnrfifHhDYfgasaacH8akY=wiFfYdH8Gipec8Eeeu0xXdbba9frFj0=OqFfea0dXdd9vqai=hGuQ8kuc9pgc9s8qqaq=dirpe0xb9q8qiLsFr0=vr0=vr0dc8meaabaqaciaacaGaaeqabaqabeGadaaakeaadaWfqaqaaiGbcYgaSjabcMgaPjabc2gaTbWcbaacbaGaf8hDaqNbauaacqGHsgIRiiGacqGFepaDdaqhaaadbaGae8NuaiLae8xzauMae8hBaWgabaGae8NzaygaaaWcbeaakiqb=bfaqzaajaGaeiikaGccbeGae0hEaG3aaWbaaSqabeaacqqFMbGzaaGccqGGSaalcuWF0baDgaqbaiabcYha8jab9Hha4jabcYcaSiab=rha0jabcMcaPiabgIKi7kab=bfaqnaaBaaaleaacqGHEisPaeqaaOGaeiikaGIae0hEaG3aaWbaaSqabeaacqqFMbGzaaGccqGG8baFcqqF4baEcqGGPaqkcqGGUaGlaaa@5628@

Provided that Eq. (2) is satisfied, Eq. (1) transforms into

lim⁡t′→τRelfP^(xf,t′|x,t)≈P∞(xf|x).
 MathType@MTEF@5@5@+=feaafiart1ev1aaatCvAUfKttLearuWrP9MDH5MBPbIqV92AaeXatLxBI9gBaebbnrfifHhDYfgasaacH8akY=wiFfYdH8Gipec8Eeeu0xXdbba9frFj0=OqFfea0dXdd9vqai=hGuQ8kuc9pgc9s8qqaq=dirpe0xb9q8qiLsFr0=vr0=vr0dc8meaabaqaciaacaGaaeqabaqabeGadaaakeaadaWfqaqaaiGbcYgaSjabcMgaPjabc2gaTbWcbaacbaGaf8hDaqNbauaacqGHsgIRiiGacqGFepaDdaqhaaadbaGae8NuaiLae8xzauMae8hBaWgabaGae8NzaygaaaWcbeaakiqb=bfaqzaajaGaeiikaGccbeGae0hEaG3aaWbaaSqabeaacqqFMbGzaaGccqGGSaalcuWF0baDgaqbaiabcYha8jab9Hha4jabcYcaSiab=rha0jabcMcaPiabgIKi7kab=bfaqnaaBaaaleaacqGHEisPaeqaaOGaeiikaGIae0hEaG3aaWbaaSqabeaacqqFMbGzaaGccqGG8baFcqqF4baEcqGGPaqkcqGGUaGlaaa@5628@

Obtaining this probabilistic description and then using it in the evolution of the slow network forms the core of multiscale, stochastic MC methods [13,16,17,20] (see also Appendix A). The solver used to compute it is called microscopic solver. A difficulty is that the relaxation time, τRelf
 MathType@MTEF@5@5@+=feaafiart1ev1aaatCvAUfKttLearuWrP9MDH5MBPbIqV92AaeXatLxBI9gBaebbnrfifHhDYfgasaacH8akY=wiFfYdH8Gipec8Eeeu0xXdbba9frFj0=OqFfea0dXdd9vqai=hGuQ8kuc9pgc9s8qqaq=dirpe0xb9q8qiLsFr0=vr0=vr0dc8meaabaqaciaacaGaaeqabaqabeGadaaakeaaiiGacqWFepaDdaqhaaWcbaacbaGae4NuaiLae4xzauMae4hBaWgabaGae4Nzaygaaaaa@33D0@, is unknown in complex systems. We have indirectly addressed this issue in Refs. [17,24] and revisit it below.

#### b. Partitioning of a stiff stochastic system

In our algorithm, one method we use follows the partitioning scheme proposed by Cao et al. [13] by identifying the fast and slow processes based on reaction propensities, a_j_(**x**, t). We identify a fast reaction subset Rf≡{R1f,R2f,...,Rmff}
 MathType@MTEF@5@5@+=feaafiart1ev1aaatCvAUfKttLearuWrP9MDH5MBPbIqV92AaeXatLxBI9gBaebbnrfifHhDYfgasaacH8akY=wiFfYdH8Gipec8Eeeu0xXdbba9frFj0=OqFfea0dXdd9vqai=hGuQ8kuc9pgc9s8qqaq=dirpe0xb9q8qiLsFr0=vr0=vr0dc8meaabaqaciaacaGaaeqabaqabeGadaaakeaaieWacqWFsbGudaahaaWcbeqaaiab=zgaMbaakiabggMi6oaacmqabaacbaGae4Nuai1aa0baaSqaaiabigdaXaqaaiab+zgaMbaakiabcYcaSiab+jfasnaaDaaaleaacqaIYaGmaeaacqGFMbGzaaGccqGGSaalcqGGUaGlcqGGUaGlcqGGUaGlcqGGSaalcqGFsbGudaqhaaWcbaGae4xBa02aaWbaaWqabeaacqGFMbGzaaaaleaacqGFMbGzaaaakiaawUhacaGL9baaaaa@458F@ with m^f ^reactions, and a slow reaction subset Rs≡{R1s,R2s,...,Rmss}
 MathType@MTEF@5@5@+=feaafiart1ev1aaatCvAUfKttLearuWrP9MDH5MBPbIqV92AaeXatLxBI9gBaebbnrfifHhDYfgasaacH8akY=wiFfYdH8Gipec8Eeeu0xXdbba9frFj0=OqFfea0dXdd9vqai=hGuQ8kuc9pgc9s8qqaq=dirpe0xb9q8qiLsFr0=vr0=vr0dc8meaabaqaciaacaGaaeqabaqabeGadaaakeaaieWacqWFsbGudaahaaWcbeqaaiab=nhaZbaakiabggMi6oaacmqabaacbaGae4Nuai1aa0baaSqaaiabigdaXaqaaiab+nhaZbaakiabcYcaSiab+jfasnaaDaaaleaacqaIYaGmaeaacqGFZbWCaaGccqGGSaalcqGGUaGlcqGGUaGlcqGGUaGlcqGGSaalcqGFsbGudaqhaaWcbaGae4xBa02aaWbaaWqabeaacqGFZbWCaaaaleaacqGFZbWCaaaakiaawUhacaGL9baaaaa@4611@ with m^s ^reactions. All species (reactants and products) participating in fast reactions are defined as *fast species*, and the remaining species as *slow species*. The state is given by **X**(t) = (**X**^f^(t),**X**^s^(t)), where Xf(t)=(X1f,X2f,..,Xnff)
 MathType@MTEF@5@5@+=feaafiart1ev1aaatCvAUfKttLearuWrP9MDH5MBPbIqV92AaeXatLxBI9gBaebbnrfifHhDYfgasaacH8akY=wiFfYdH8Gipec8Eeeu0xXdbba9frFj0=OqFfea0dXdd9vqai=hGuQ8kuc9pgc9s8qqaq=dirpe0xb9q8qiLsFr0=vr0=vr0dc8meaabaqaciaacaGaaeqabaqabeGadaaakeaaieqacqWFybawdaahaaWcbeqaaiab=zgaMbaakiabcIcaOGqaaiab+rha0jabcMcaPiabg2da9maabmaabaGae4hwaG1aa0baaSqaaiabigdaXaqaaiab+zgaMbaakiabcYcaSiab+HfaynaaDaaaleaacqaIYaGmaeaacqGFMbGzaaGccqGGSaalcqGGUaGlcqGGUaGlcqGGSaalcqGFybawdaqhaaWcbaGae4NBa42aaWbaaWqabeaacqGFMbGzaaaaleaacqGFMbGzaaaakiaawIcacaGLPaaaaaa@468D@ and Xs(t)=(X1s,X2s,..,Xnss)
 MathType@MTEF@5@5@+=feaafiart1ev1aaatCvAUfKttLearuWrP9MDH5MBPbIqV92AaeXatLxBI9gBaebbnrfifHhDYfgasaacH8akY=wiFfYdH8Gipec8Eeeu0xXdbba9frFj0=OqFfea0dXdd9vqai=hGuQ8kuc9pgc9s8qqaq=dirpe0xb9q8qiLsFr0=vr0=vr0dc8meaabaqaciaacaGaaeqabaqabeGadaaakeaaieqacqWFybawdaahaaWcbeqaaiab=nhaZbaakiabcIcaOGqaaiab+rha0jabcMcaPiabg2da9maabmaabaGae4hwaG1aa0baaSqaaiabigdaXaqaaiab+nhaZbaakiabcYcaSiab+HfaynaaDaaaleaacqaIYaGmaeaacqGFZbWCaaGccqGGSaalcqGGUaGlcqGGUaGlcqGGSaalcqGFybawdaqhaaWcbaGae4NBa42aaWbaaWqabeaacqGFZbWCaaaaleaacqGFZbWCaaaakiaawIcacaGLPaaaaaa@470F@ are the states of the fast and slow species, respectively. n^f ^and n^s ^are the number of fast and slow species, respectively, and n = n^f^+n^s ^is the total number of species. The propensity function of the slow reaction Rjs
 MathType@MTEF@5@5@+=feaafiart1ev1aaatCvAUfKttLearuWrP9MDH5MBPbIqV92AaeXatLxBI9gBaebbnrfifHhDYfgasaacH8akY=wiFfYdH8Gipec8Eeeu0xXdbba9frFj0=OqFfea0dXdd9vqai=hGuQ8kuc9pgc9s8qqaq=dirpe0xb9q8qiLsFr0=vr0=vr0dc8meaabaqaciaacaGaaeqabaqabeGadaaakeaaieaacqWFsbGudaqhaaWcbaGae8NAaOgabaGae83Camhaaaaa@30CF@ and of the fast reaction Rjf
 MathType@MTEF@5@5@+=feaafiart1ev1aaatCvAUfKttLearuWrP9MDH5MBPbIqV92AaeXatLxBI9gBaebbnrfifHhDYfgasaacH8akY=wiFfYdH8Gipec8Eeeu0xXdbba9frFj0=OqFfea0dXdd9vqai=hGuQ8kuc9pgc9s8qqaq=dirpe0xb9q8qiLsFr0=vr0=vr0dc8meaabaqaciaacaGaaeqabaqabeGadaaakeaaieaacqWFsbGudaqhaaWcbaGae8NAaOgabaGae8Nzaygaaaaa@30B5@ at state **X**(t) = (**x**^**f**^,**x**^**s**^) can be generally written as ajs(x;t)=ajs(xf,xs;t)
 MathType@MTEF@5@5@+=feaafiart1ev1aaatCvAUfKttLearuWrP9MDH5MBPbIqV92AaeXatLxBI9gBaebbnrfifHhDYfgasaacH8akY=wiFfYdH8Gipec8Eeeu0xXdbba9frFj0=OqFfea0dXdd9vqai=hGuQ8kuc9pgc9s8qqaq=dirpe0xb9q8qiLsFr0=vr0=vr0dc8meaabaqaciaacaGaaeqabaqabeGadaaakeaaieaacqWFHbqydaqhaaWcbaGae8NAaOgabaGae83CamhaaOWaaeWaaeaaieqacqGF4baEcqGG7aWocqWF0baDaiaawIcacaGLPaaacqGH9aqpcqWFHbqydaqhaaWcbaGae8NAaOgabaGae83CamhaaOWaaeWaaeaacqGF4baEdaahaaWcbeqaaiab+zgaMbaakiabcYcaSiab+Hha4naaCaaaleqabaGae43CamhaaOGaei4oaSJae8hDaqhacaGLOaGaayzkaaaaaa@4695@, and ajf(x;t)=ajf(xf;t)
 MathType@MTEF@5@5@+=feaafiart1ev1aaatCvAUfKttLearuWrP9MDH5MBPbIqV92AaeXatLxBI9gBaebbnrfifHhDYfgasaacH8akY=wiFfYdH8Gipec8Eeeu0xXdbba9frFj0=OqFfea0dXdd9vqai=hGuQ8kuc9pgc9s8qqaq=dirpe0xb9q8qiLsFr0=vr0=vr0dc8meaabaqaciaacaGaaeqabaqabeGadaaakeaaieaacqWFHbqydaqhaaWcbaGae8NAaOgabaGae8NzaygaaOWaaeWaaeaaieqacqGF4baEcqGG7aWocqWF0baDaiaawIcacaGLPaaacqGH9aqpcqWFHbqydaqhaaWcbaGae8NAaOgabaGae8NzaygaaOWaaeWaaeaacqGF4baEdaahaaWcbeqaaiab+zgaMbaakiabcUda7iab=rha0bGaayjkaiaawMcaaaaa@426C@, respectively.

Partitioning is done according to a user-defined threshold of the propensities. The choice of this threshold is influenced by the required accuracy and the computational cost. Using simple model systems [17], it was found that a separation of time scales of at least 2 orders of magnitude is necessary for the MSMC method to be accurate and computationally more efficient; this value is further discussed below using new and more complex examples. This ranked propensity-based partitioning scheme does not always yield a single cut-off point, especially for large networks with multiple gaps in time scales. Furthermore, it does not identify the relaxation time. We propose the use of the computational singular perturbation (CSP) technique to assist with these difficulties.

##### Computational Singular Perturbation (CSP)-assisted stochastic partitioning

The computational singular perturbation (CSP) technique was introduced by Lam, Goussis and co-workers, e.g., [25,26], as a diagnostic tool for gaining physical insight into the reaction dynamics of large complex networks, and as an accelerated deterministic solver for stiff networks. Our principal interest in the CSP approach is in its diagnostic power, specifically its ability to identify the species present in QE, and the dominating reactions in the fast and slow modes. We refer to our use of CSP techniques in network partitioning as a "CSP-assisted stochastic partitioning".

Consider the deterministic representation of the dynamics of a reaction network

g(x)=dXdt=∑i=1mυiai(x).
 MathType@MTEF@5@5@+=feaafiart1ev1aaatCvAUfKttLearuWrP9MDH5MBPbIqV92AaeXatLxBI9gBaebbnrfifHhDYfgasaacH8akY=wiFfYdH8Gipec8Eeeu0xXdbba9frFj0=OqFfea0dXdd9vqai=hGuQ8kuc9pgc9s8qqaq=dirpe0xb9q8qiLsFr0=vr0=vr0dc8meaabaqaciaacaGaaeqabaqabeGadaaakeaaieqacqWFNbWzcqGGOaakcqWF4baEcqGGPaqkcqGH9aqpdaWcaaqaaGqaaiab+rgaKjab=Hfaybqaaiab+rgaKjab+rha0baacqGH9aqpdaaeWbqaaGGadiab9v8a1naaBaaaleaacqWFPbqAaeqaaOGae4xyae2aaSbaaSqaaiab+LgaPbqabaGccqGGOaakcqWF4baEcqGGPaqkaSqaaiab+LgaPjabg2da9iabigdaXaqaaiab+1gaTbqdcqGHris5aOGaeiOla4caaa@49BB@

**g(x) **is an n-dimensional vector, such that g_i_(t) = dX_i_/dt describes the rate of change of species S_i_. Differentiating (4), yields

dg(x)dt=∂g∂X⋅dXdt=J¯¯⋅g,
MathType@MTEF@5@5@+=feaafiart1ev1aaatCvAUfKttLearuWrP9MDH5MBPbIqV92AaeXatLxBI9gBaebbnrfifHhDYfgasaacH8akY=wiFfYdH8Gipec8Eeeu0xXdbba9frFj0=OqFfea0dXdd9vqai=hGuQ8kuc9pgc9s8qqaq=dirpe0xb9q8qiLsFr0=vr0=vr0dc8meaabaqaciaacaGaaeqabaqabeGadaaakeaadaWcaaqaaGqaaiab=rgaKHqabiab+DgaNjabcIcaOiab+Hha4jabcMcaPaqaaiab=rgaKjab=rha0baacqGH9aqpdaWcaaqaaGGaciab9jGi2kab+DgaNbqaaiab9jGi2kab+HfaybaacqGHflY1daWcaaqaaiab=rgaKjab+Hfaybqaaiab=rgaKjab=rha0baacqGH9aqpdaadbaqaaiab+PeakbaacqGHflY1cqGFNbWzcqGGSaalaaa@49F0@

where J¯¯=∂g∂X
MathType@MTEF@5@5@+=feaafiart1ev1aaatCvAUfKttLearuWrP9MDH5MBPbIqV92AaeXatLxBI9gBaebbnrfifHhDYfgasaacH8akY=wiFfYdH8Gipec8Eeeu0xXdbba9frFj0=OqFfea0dXdd9vqai=hGuQ8kuc9pgc9s8qqaq=dirpe0xb9q8qiLsFr0=vr0=vr0dc8meaabaqaciaacaGaaeqabaqabeGadaaakeaadaadbaqaaGqabiab=PeakbaacqGH9aqpdaWcaaqaaGGaciab+jGi2kab=DgaNbqaaiab+jGi2kab=Hfaybaaaaa@3446@ is the Jacobian matrix of **g**. In general, the jacobian matrix, J¯¯
MathType@MTEF@5@5@+=feaafiart1ev1aaatCvAUfKttLearuWrP9MDH5MBPbIqV92AaeXatLxBI9gBaebbnrfifHhDYfgasaacH8akY=wiFfYdH8Gipec8Eeeu0xXdbba9frFj0=OqFfea0dXdd9vqai=hGuQ8kuc9pgc9s8qqaq=dirpe0xb9q8qiLsFr0=vr0=vr0dc8meaabaqaciaacaGaaeqabaqabeGadaaakeaadaadbaqaaGqabiab=Peakbaaaaa@2DE0@ is not a diagonal matrix. We perform a linear transformation on **g**,

f=b¯¯⋅g,
MathType@MTEF@5@5@+=feaafiart1ev1aaatCvAUfKttLearuWrP9MDH5MBPbIqV92AaeXatLxBI9gBaebbnrfifHhDYfgasaacH8akY=wiFfYdH8Gipec8Eeeu0xXdbba9frFj0=OqFfea0dXdd9vqai=hGuQ8kuc9pgc9s8qqaq=dirpe0xb9q8qiLsFr0=vr0=vr0dc8meaabaqaciaacaGaaeqabaqabeGadaaakeaaieqacqWFMbGzcqGH9aqpdaadbaqaaiab=jgaIbaacqGHflY1cqWFNbWzcqGGSaalaaa@34E4@

such that the transformed mode **f **evolves in a decoupled manner,

dfdt=Λ¯¯⋅f.
MathType@MTEF@5@5@+=feaafiart1ev1aaatCvAUfKttLearuWrP9MDH5MBPbIqV92AaeXatLxBI9gBaebbnrfifHhDYfgasaacH8akY=wiFfYdH8Gipec8Eeeu0xXdbba9frFj0=OqFfea0dXdd9vqai=hGuQ8kuc9pgc9s8qqaq=dirpe0xb9q8qiLsFr0=vr0=vr0dc8meaabaqaciaacaGaaeqabaqabeGadaaakeaadaWcaaqaaGqaaiab=rgaKHqabiab+zgaMbqaaiab=rgaKjab=rha0baacqGH9aqpdaadbaqaaGGabiab9T5ambaacqGHflY1cqGFMbGzcqGGUaGlaaa@3933@

The matrix b¯¯
MathType@MTEF@5@5@+=feaafiart1ev1aaatCvAUfeBSjuyZL2yd9gzLbvyNv2CaerbwvMCKfMBHbqedmvETj2BSbqee0evGueE0jxyaibaieIgFLIOYR2NHOxjYhrPYhrPYpI8F4rqqrFfpeea0xe9Lq=Jc9vqaqpepm0xbbG8FasPYRqj0=yi0lXdbba9pGe9qqFf0dXdHuk9fr=xfr=xfrpiWZqaaeaabiGaaiaacaqabeaabeqacmaaaOqaamaameaabaWexLMBb50ujbqegCuAVzxyU5wAGi0BVTgaiqqacqWFIbGyaaaaaa@3E4D@ consists of a set of n linearly independent row vectors, **b**_i_, and is chosen appropriately to decouple the evolution of the transformed modes, **f**. Λ¯¯
MathType@MTEF@5@5@+=feaafiart1ev1aaatCvAUfKttLearuWrP9MDH5MBPbIqV92AaeXatLxBI9gBaebbnrfifHhDYfgasaacH8akY=wiFfYdH8Gipec8Eeeu0xXdbba9frFj0=OqFfea0dXdd9vqai=hGuQ8kuc9pgc9s8qqaq=dirpe0xb9q8qiLsFr0=vr0=vr0dc8meaabaqaciaacaGaaeqabaqabeGadaaakeaadaadbaqaaGGabiab=T5ambaaaaa@2E37@ is a diagonal matrix with the diagonal elements, |Λ_ii_|, arranged in the order of descending magnitudes. Every vector, **b**_**i**_, is associated with a vector, bi*
 MathType@MTEF@5@5@+=feaafiart1ev1aaatCvAUfKttLearuWrP9MDH5MBPbIqV92AaeXatLxBI9gBaebbnrfifHhDYfgasaacH8akY=wiFfYdH8Gipec8Eeeu0xXdbba9frFj0=OqFfea0dXdd9vqai=hGuQ8kuc9pgc9s8qqaq=dirpe0xb9q8qiLsFr0=vr0=vr0dc8meaabaqaciaacaGaaeqabaqabeGadaaakeaaieqacqWFIbGydaqhaaWcbaGae8xAaKgabaGamaiScQcaQaaaaaa@3197@ through the biorthonormality condition, such that,

bi*⋅bj=1 (i=j) and bi*⋅bj=0 (i≠j)
 MathType@MTEF@5@5@+=feaafiart1ev1aaatCvAUfKttLearuWrP9MDH5MBPbIqV92AaeXatLxBI9gBaebbnrfifHhDYfgasaacH8akY=wiFfYdH8Gipec8Eeeu0xXdbba9frFj0=OqFfea0dXdd9vqai=hGuQ8kuc9pgc9s8qqaq=dirpe0xb9q8qiLsFr0=vr0=vr0dc8meaabaqaciaacaGaaeqabaqabeGadaaakeaaieqacqWFIbGydaqhaaWcbaGae8xAaKgabaGamaiPcQcaQaaakiabgwSixlab=jgaInaaBaaaleaacqWFQbGAaeqaaOGaeyypa0JaeGymaeJaeeiiaaIaeiikaGccbaGae4xAaKMaeyypa0Jae4NAaOMaeiykaKIaeeiiaaIae4xyaeMae4NBa4Mae4hzaqMaeeiiaaIae8Nyai2aa0baaSqaaiab=LgaPbqaaiadasQGQaGkaaGccqGHflY1cqWFIbGydaWgaaWcbaGae8NAaOgabeaakiabg2da9iabicdaWiabbccaGiabcIcaOiab+LgaPjabgcMi5kab+PgaQjabcMcaPaaa@5746@

The matrix b*¯¯
MathType@MTEF@5@5@+=feaafiart1ev1aaatCvAUfKttLearuWrP9MDH5MBPbIqV92AaeXatLxBI9gBaebbnrfifHhDYfgasaacH8akY=wiFfYdH8Gipec8Eeeu0xXdbba9frFj0=OqFfea0dXdd9vqai=hGuQ8kuc9pgc9s8qqaq=dirpe0xb9q8qiLsFr0=vr0=vr0dc8meaabaqaciaacaGaaeqabaqabeGadaaakeaadaadbaqaaGqabiab=jgaInaaCaaaleqabaGamGjVcQcaQaaaaaaaaa@3075@ consists of n linearly independent column vectors, bi*
 MathType@MTEF@5@5@+=feaafiart1ev1aaatCvAUfKttLearuWrP9MDH5MBPbIqV92AaeXatLxBI9gBaebbnrfifHhDYfgasaacH8akY=wiFfYdH8Gipec8Eeeu0xXdbba9frFj0=OqFfea0dXdd9vqai=hGuQ8kuc9pgc9s8qqaq=dirpe0xb9q8qiLsFr0=vr0=vr0dc8meaabaqaciaacaGaaeqabaqabeGadaaakeaaieqacqWFIbGydaqhaaWcbaGae8xAaKgabaGamaiScQcaQaaaaaa@3197@, and is called the basis vectors set. The matrix b¯¯
MathType@MTEF@5@5@+=feaafiart1ev1aaatCvAUfeBSjuyZL2yd9gzLbvyNv2CaerbwvMCKfMBHbqedmvETj2BSbqee0evGueE0jxyaibaieIgFLIOYR2NHOxjYhrPYhrPYpI8F4rqqrFfpeea0xe9Lq=Jc9vqaqpepm0xbbG8FasPYRqj0=yi0lXdbba9pGe9qqFf0dXdHuk9fr=xfr=xfrpiWZqaaeaabiGaaiaacaqabeaabeqacmaaaOqaamaameaabaWexLMBb50ujbqegCuAVzxyU5wAGi0BVTgaiqqacqWFIbGyaaaaaa@3E4D@, which is the inverse of the basis vector set, is called the dual vector set. Lam defined an ideal basis vector set, b*¯¯
MathType@MTEF@5@5@+=feaafiart1ev1aaatCvAUfKttLearuWrP9MDH5MBPbIqV92AaeXatLxBI9gBaebbnrfifHhDYfgasaacH8akY=wiFfYdH8Gipec8Eeeu0xXdbba9frFj0=OqFfea0dXdd9vqai=hGuQ8kuc9pgc9s8qqaq=dirpe0xb9q8qiLsFr0=vr0=vr0dc8meaabaqaciaacaGaaeqabaqabeGadaaakeaadaadbaqaaGqabiab=jgaInaaCaaaleqabaGamGjVcQcaQaaaaaaaaa@3075@ as one that completely decouples the evolution of the transformed modes [25,26]. The CSP method provides an iterative refinement procedure [25,27] to obtain the ideal basis vector set from a random trial basis set.

For linear systems, the Jacobian matrix is independent of time, and thus, the right hand eigenvectors of the Jacobian, J¯¯
MathType@MTEF@5@5@+=feaafiart1ev1aaatCvAUfKttLearuWrP9MDH5MBPbIqV92AaeXatLxBI9gBaebbnrfifHhDYfgasaacH8akY=wiFfYdH8Gipec8Eeeu0xXdbba9frFj0=OqFfea0dXdd9vqai=hGuQ8kuc9pgc9s8qqaq=dirpe0xb9q8qiLsFr0=vr0=vr0dc8meaabaqaciaacaGaaeqabaqabeGadaaakeaadaadbaqaaGqabiab=Peakbaaaaa@2DE0@, can be conveniently used as the ideal basis vector, b*¯¯
MathType@MTEF@5@5@+=feaafiart1ev1aaatCvAUfKttLearuWrP9MDH5MBPbIqV92AaeXatLxBI9gBaebbnrfifHhDYfgasaacH8akY=wiFfYdH8Gipec8Eeeu0xXdbba9frFj0=OqFfea0dXdd9vqai=hGuQ8kuc9pgc9s8qqaq=dirpe0xb9q8qiLsFr0=vr0=vr0dc8meaabaqaciaacaGaaeqabaqabeGadaaakeaadaadbaqaaGqabiab=jgaInaaCaaaleqabaGamGjVcQcaQaaaaaaaaa@3075@(assuming J¯¯
MathType@MTEF@5@5@+=feaafiart1ev1aaatCvAUfKttLearuWrP9MDH5MBPbIqV92AaeXatLxBI9gBaebbnrfifHhDYfgasaacH8akY=wiFfYdH8Gipec8Eeeu0xXdbba9frFj0=OqFfea0dXdd9vqai=hGuQ8kuc9pgc9s8qqaq=dirpe0xb9q8qiLsFr0=vr0=vr0dc8meaabaqaciaacaGaaeqabaqabeGadaaakeaadaadbaqaaGqabiab=Peakbaaaaa@2DE0@ is a perfect matrix of linearly independent eigenvectors). In this case, b¯¯
MathType@MTEF@5@5@+=feaafiart1ev1aaatCvAUfeBSjuyZL2yd9gzLbvyNv2CaerbwvMCKfMBHbqedmvETj2BSbqee0evGueE0jxyaibaieIgFLIOYR2NHOxjYhrPYhrPYpI8F4rqqrFfpeea0xe9Lq=Jc9vqaqpepm0xbbG8FasPYRqj0=yi0lXdbba9pGe9qqFf0dXdHuk9fr=xfr=xfrpiWZqaaeaabiGaaiaacaqabeaabeqacmaaaOqaamaameaabaWexLMBb50ujbqegCuAVzxyU5wAGi0BVTgaiqqacqWFIbGyaaaaaa@3E4D@ is the inverse of the eigenvector matrix of J¯¯
MathType@MTEF@5@5@+=feaafiart1ev1aaatCvAUfKttLearuWrP9MDH5MBPbIqV92AaeXatLxBI9gBaebbnrfifHhDYfgasaacH8akY=wiFfYdH8Gipec8Eeeu0xXdbba9frFj0=OqFfea0dXdd9vqai=hGuQ8kuc9pgc9s8qqaq=dirpe0xb9q8qiLsFr0=vr0=vr0dc8meaabaqaciaacaGaaeqabaqabeGadaaakeaadaadbaqaaGqabiab=Peakbaaaaa@2DE0@. For nonlinear systems, the eigenvectors of J¯¯
MathType@MTEF@5@5@+=feaafiart1ev1aaatCvAUfKttLearuWrP9MDH5MBPbIqV92AaeXatLxBI9gBaebbnrfifHhDYfgasaacH8akY=wiFfYdH8Gipec8Eeeu0xXdbba9frFj0=OqFfea0dXdd9vqai=hGuQ8kuc9pgc9s8qqaq=dirpe0xb9q8qiLsFr0=vr0=vr0dc8meaabaqaciaacaGaaeqabaqabeGadaaakeaadaadbaqaaGqabiab=Peakbaaaaa@2DE0@ are time-dependent, and do not constitute an ideal basis vector set. Lam [25] proved that using the eigenvectors as an ideal basis set for nonlinear systems provides a leading order accuracy. Since we employ the CSP technique as a partitioning guide, leading order accuracy suffices as our examples indicate.

The elements Λ_ii _represent the time scales of the system, with a possible gap in the magnitude of Λ_ii _reflecting a time-scale separation. Assuming that such a gap can be identified, i.e., the system is stiff, we get a set of n^f ^fast modes, **f**^**f**^, and n^s ^slow modes, **f**^**s**^. If the separation is large enough, i.e., min⁡(|Λiif|)≫max⁡(|Λiis|)
 MathType@MTEF@5@5@+=feaafiart1ev1aaatCvAUfKttLearuWrP9MDH5MBPbIqV92AaeXatLxBI9gBaebbnrfifHhDYfgasaacH8akY=wiFfYdH8Gipec8Eeeu0xXdbba9frFj0=OqFfea0dXdd9vqai=hGuQ8kuc9pgc9s8qqaq=dirpe0xb9q8qiLsFr0=vr0=vr0dc8meaabaqaciaacaGaaeqabaqabeGadaaakeaacyGGTbqBcqGGPbqAcqGGUbGBdaqadaqaamaaemaabaGaeu4MdW0aa0baaSqaaGqaaiab=LgaPjab=LgaPbqaaiab=zgaMbaaaOGaay5bSlaawIa7aaGaayjkaiaawMcaaiablUMi=iGbc2gaTjabcggaHjabcIha4naabmaabaWaaqWaaeaacqqHBoatdaqhaaWcbaGae8xAaKMae8xAaKgabaGae83CamhaaaGccaGLhWUaayjcSdaacaGLOaGaayzkaaaaaa@4B20@, then the fast modes relax rapidly, and the reduced system consisting of only the slow modes can be evolved over the low-dimensional manifold using larger time increments. While such a transformation in deterministic systems is helpful in removing stiffness from the system and reducing the system dimension, here we are more interested in extracting the physical meaning of the modes, and identifying reactions and/or species that contribute to the fast modes.

Lam [25] defined the mode participation index (PI) of reaction j in mode i as

Pji≡Bjiaj∑r=1m|Briar|+O(|bi⋅Δy*/Δt*|)i=1,2,...n, j=1,2,...,m
 MathType@MTEF@5@5@+=feaafiart1ev1aaatCvAUfKttLearuWrP9MDH5MBPbIqV92AaeXatLxBI9gBaebbnrfifHhDYfgasaacH8akY=wiFfYdH8Gipec8Eeeu0xXdbba9frFj0=OqFfea0dXdd9vqai=hGuQ8kuc9pgc9s8qqaq=dirpe0xb9q8qiLsFr0=vr0=vr0dc8meaabaqaciaacaGaaeqabaqabeGadaaakeaaieaacqWFqbaudaqhaaWcbaGae8NAaOgabaGae8xAaKgaaOGaeyyyIO7aaSaaaeaacqWFcbGqdaqhaaWcbaGae8NAaOgabaGae8xAaKgaaOGae8xyae2aaSbaaSqaaiab=PgaQbqabaaakeaadaaeWbqaamaaemaabaGae8Nqai0aa0baaSqaaiab=jhaYbqaaiab=LgaPbaakiab=fgaHnaaBaaaleaacqWFYbGCaeqaaaGccaGLhWUaayjcSdGaey4kaSIae83ta80aaeWaaeaadaabdaqaaiab=jgaInaaBaaaleaacqWFPbqAaeqaaOGaeyyXICTaeyiLdqKae8xEaK3aiai1CaaaleqcasDaiai1cWaGulOkaOcaaOGaei4la8IaeyiLdqKae8hDaq3aiaiVCaaaleqcaYBaiaiVcWaGulOkaOcaaaGccaGLhWUaayjcSdaacaGLOaGaayzkaaaaleaacqWFYbGCcqGH9aqpcqaIXaqmaeaacqWFTbqBa0GaeyyeIuoaaaGccqWFPbqAcqGH9aqpcqaIXaqmcqGGSaalcqaIYaGmcqGGSaalcqGGUaGlcqGGUaGlcqGGUaGlcqWFUbGBcqGGSaalcqqGGaaicqWFQbGAcqGH9aqpcqaIXaqmcqGGSaalcqaIYaGmcqGGSaalcqGGUaGlcqGGUaGlcqGGUaGlcqGGSaalcqWFTbqBaaa@8037@

where Bji
 MathType@MTEF@5@5@+=feaafiart1ev1aaatCvAUfKttLearuWrP9MDH5MBPbIqV92AaeXatLxBI9gBaebbnrfifHhDYfgasaacH8akY=wiFfYdH8Gipec8Eeeu0xXdbba9frFj0=OqFfea0dXdd9vqai=hGuQ8kuc9pgc9s8qqaq=dirpe0xb9q8qiLsFr0=vr0=vr0dc8meaabaqaciaacaGaaeqabaqabeGadaaakeaaieaacqWFcbGqdaqhaaWcbaGae8NAaOgabaGae8xAaKgaaaaa@309B@ is given by the dot product **b**_i _⊙ **υ**_j_. Δy* and Δt* are user-defined values of acceptable error in temporal solution provided by the CSP method. To simplify our analysis, we neglect the second term, O(|b_i_·Δy*/Δt*|), that sets the threshold for magnitude of relaxed f. The participation index, Pji
 MathType@MTEF@5@5@+=feaafiart1ev1aaatCvAUfKttLearuWrP9MDH5MBPbIqV92AaeXatLxBI9gBaebbnrfifHhDYfgasaacH8akY=wiFfYdH8Gipec8Eeeu0xXdbba9frFj0=OqFfea0dXdd9vqai=hGuQ8kuc9pgc9s8qqaq=dirpe0xb9q8qiLsFr0=vr0=vr0dc8meaabaqaciaacaGaaeqabaqabeGadaaakeaaieaacqWFqbaudaqhaaWcbaGae8NAaOgabaGae8xAaKgaaaaa@30B7@, for the fast modes (i = 1, 2, ..., n^f^) gives the fractional contribution of reaction j to the fast mode, i.

Herein, the CSP method is used at any stochastic state to understand the instantaneous time scales and appropriately partition the network at that state. This constitutes our second partitioning method. The evolution of the reaction network is still carried out in a stochastic manner, using the HyMSMC method. The Jacobian at a stochastic state can be approximated using the corresponding deterministic model or its variant where the mean-field propensity functions are used instead of the deterministic reaction rates. We propose the latter approach. In addition, we recommend using an analytical Jacobian, if possible, due to its higher accuracy and lower computational cost. Aside from partitioning, the CSP method provides an estimate of the relaxation time of the system that could be used for converging the fast network. Since there are multiple eigenvalues, and thus multiple time scales, in our implementation we choose the smallest eigenvalue (in magnitude) from the n^f ^fast modes, to relax the fastest dynamics. We discuss this concept further below and in the results section.

Obviously, there is a computational overhead associated with the evaluation of the Jacobian and the eigenvalue analysis. This overhead depends on whether the system is linear (eigenvalue analysis is done only once) or not, the system size and the frequency of carrying out the CSP analysis. Ideally, network partitioning, either CSP-assisted or ranked propensity-based, should be performed prior to every call to the microscopic solver. Practically however, when the trajectory evolves fairly smoothly and gradually, a few calls of the CSP routine during a simulation are sufficient. For the networks considered in this work, the overhead of CSP is negligible in comparison to the cost of a stochastic simulation (see results section). Below, we demonstrate the potential of the CSP technique using several examples and discuss the CPU of the CSP analysis for the most complicated example.

#### c. Evolution of the slow network

Once the QE PDF of the fast network has been obtained, the slow network is evolved. Appendix A discusses various approximations. The *slow-scale approximation *[13] evolves the slow network using the slow-scale propensities as the effective transition probabilities of the slow reactions. The slow-scale propensity of reaction Rjs
 MathType@MTEF@5@5@+=feaafiart1ev1aaatCvAUfKttLearuWrP9MDH5MBPbIqV92AaeXatLxBI9gBaebbnrfifHhDYfgasaacH8akY=wiFfYdH8Gipec8Eeeu0xXdbba9frFj0=OqFfea0dXdd9vqai=hGuQ8kuc9pgc9s8qqaq=dirpe0xb9q8qiLsFr0=vr0=vr0dc8meaabaqaciaacaGaaeqabaqabeGadaaakeaaieaacqWFsbGudaqhaaWcbaGae8NAaOgabaGae83Camhaaaaa@30CF@ is the expectation of the propensity function, ajs
 MathType@MTEF@5@5@+=feaafiart1ev1aaatCvAUfKttLearuWrP9MDH5MBPbIqV92AaeXatLxBI9gBaebbnrfifHhDYfgasaacH8akY=wiFfYdH8Gipec8Eeeu0xXdbba9frFj0=OqFfea0dXdd9vqai=hGuQ8kuc9pgc9s8qqaq=dirpe0xb9q8qiLsFr0=vr0=vr0dc8meaabaqaciaacaGaaeqabaqabeGadaaakeaaieaacqWFHbqydaqhaaWcbaGae8NAaOgabaGae83Camhaaaaa@30ED@(**x**^**f**^, **x**^**s**^), over the temporal QE PDF given by

ajs¯(x)=∑xf′P^∞(xf′|x)⋅ajs(xf′,xs),
 MathType@MTEF@5@5@+=feaafiart1ev1aaatCvAUfKttLearuWrP9MDH5MBPbIqV92AaeXatLxBI9gBaebbnrfifHhDYfgasaacH8akY=wiFfYdH8Gipec8Eeeu0xXdbba9frFj0=OqFfea0dXdd9vqai=hGuQ8kuc9pgc9s8qqaq=dirpe0xb9q8qiLsFr0=vr0=vr0dc8meaabaqaciaacaGaaeqabaqabeGadaaakeaadaqdaaqaaGqaaiab=fgaHnaaDaaaleaacqWFQbGAaeaacqWFZbWCaaaaaOGaeiikaGccbeGae4hEaGNaeiykaKIaeyypa0ZaaabuaeaacuWFqbaugaqcamaaBaaaleaacqGHEisPaeqaaOGaeiikaGIae4hEaG3aaWbaaSqabeaacuGFMbGzgaqbaaaakiabcYha8jab+Hha4jabcMcaPiabgwSixlab=fgaHnaaDaaaleaacqWFQbGAaeaacqWFZbWCaaGcdaqadaqaaiab+Hha4naaCaaaleqabaGaf4NzayMbauaaaaGccqGGSaalcqGF4baEdaahaaWcbeqaaiab+nhaZbaaaOGaayjkaiaawMcaaaWcbaGae8hEaG3aaWbaaWqabeaacuWFMbGzgaqbaaaaaSqab0GaeyyeIuoakiabcYcaSaaa@54F0@

where the summation is performed over all the equilibrium states **x**^**f' **^visited by the fast species [13]. Slow reactions are picked and the elapsed time is computed the same way as in the SSA.

After a slow reaction has been picked, the populations of the species need to be updated. This is not as straightforward, and while critical, has been largely overlooked. A rigorous way of picking slow reactions and subsequently updating the slow network is based on a joint PDF [17] (see Appendix A). In this work, we use the last state present after the relaxation criterion (see section d) has been met. In doing this, we minimize the computational effort and also ensure that the fast state belongs to stationary PDF P_∞_(**x**^**f **^| **x**). In some cases, the selected slow reaction cannot be executed from some states of the fast species, e.g., a reactant species with zero population, even though these states might be visited frequently [17]. Care is taken that the chosen slow reaction can be executed from that state by checking the feasibility of firing the chosen slow reaction from the selected state. Failure to satisfy this condition calls for additional simulation of the relaxed fast network until the chosen slow reaction can occur from the current state of fast species or selection from a database of states from P_∞_(**x**^**f **^| **x**). This eliminates the problem of having negative populations.

#### d. Relaxation of the Fast Network

The next question is how does one gauge the relaxation of the fast network and decide the time, τ_Eqm_, required to obtain an accurate approximation of the stationary PDF, P_→_(**x**^**f **^| **x**), or the slow-scale propensities, ajs¯
 MathType@MTEF@5@5@+=feaafiart1ev1aaatCvAUfKttLearuWrP9MDH5MBPbIqV92AaeXatLxBI9gBaebbnrfifHhDYfgasaacH8akY=wiFfYdH8Gipec8Eeeu0xXdbba9frFj0=OqFfea0dXdd9vqai=hGuQ8kuc9pgc9s8qqaq=dirpe0xb9q8qiLsFr0=vr0=vr0dc8meaabaqaciaacaGaaeqabaqabeGadaaakeaadaqdaaqaaGqaaiab=fgaHnaaDaaaleaacqWFQbGAaeaacqWFZbWCaaaaaaaa@30FE@**(x) **? E et al. [20] decide τ_Eqm _via an implicit relation to the deviation of the numerical estimate of the slow-scale propensity from its true value. Salis and Kaznessis [16] relax the fast network for a user-defined number of MC events, MCEEqmf
 MathType@MTEF@5@5@+=feaafiart1ev1aaatCvAUfKttLearuWrP9MDH5MBPbIqV92AaeXatLxBI9gBaebbnrfifHhDYfgasaacH8akY=wiFfYdH8Gipec8Eeeu0xXdbba9frFj0=OqFfea0dXdd9vqai=hGuQ8kuc9pgc9s8qqaq=dirpe0xb9q8qiLsFr0=vr0=vr0dc8meaabaqaciaacaGaaeqabaqabeGadaaakeaaieaacqWFnbqtcqWFdbWqcqWFfbqrdaqhaaWcbaGae8xrauKae8xCaeNae8xBa0gabaGae8Nzaygaaaaa@3541@. A predetermined choice of τ_Eqm _or MCEEqmf
 MathType@MTEF@5@5@+=feaafiart1ev1aaatCvAUfKttLearuWrP9MDH5MBPbIqV92AaeXatLxBI9gBaebbnrfifHhDYfgasaacH8akY=wiFfYdH8Gipec8Eeeu0xXdbba9frFj0=OqFfea0dXdd9vqai=hGuQ8kuc9pgc9s8qqaq=dirpe0xb9q8qiLsFr0=vr0=vr0dc8meaabaqaciaacaGaaeqabaqabeGadaaakeaaieaacqWFnbqtcqWFdbWqcqWFfbqrdaqhaaWcbaGae8xrauKae8xCaeNae8xBa0gabaGae8Nzaygaaaaa@3541@ overlooks the likelihood of the relaxation time varying with time, and may make the multiscale simulation inaccurate and potentially inefficient. Thus, we need a more generic relaxation criterion that decides the relaxation time (or number of MC events) of the fast network on-the-fly. This issue is addressed next.

The slow-scale propensity is the first moment (or mean) of the propensity of the slow reaction, evaluated over the entire stationary PDF P_∞_(x^**f **^| **x**). In this work, we test convergence of the slow-scale propensities rather than of the entire PDF. We propose the 2 sample t-test to check for convergence of the slow-scale propensities. The 2 sample t-test is a commonly used statistical tool to verify with a certain degree of confidence, if two independently drawn samples of a random variable come from the same probability distribution. In the current context, the state of the fast species (and also the propensity, ajs
 MathType@MTEF@5@5@+=feaafiart1ev1aaatCvAUfKttLearuWrP9MDH5MBPbIqV92AaeXatLxBI9gBaebbnrfifHhDYfgasaacH8akY=wiFfYdH8Gipec8Eeeu0xXdbba9frFj0=OqFfea0dXdd9vqai=hGuQ8kuc9pgc9s8qqaq=dirpe0xb9q8qiLsFr0=vr0=vr0dc8meaabaqaciaacaGaaeqabaqabeGadaaakeaaieaacqWFHbqydaqhaaWcbaGae8NAaOgabaGae83Camhaaaaa@30ED@(**x**^**f**^,**x**^**s**^), of the slow reaction) is the random variable, such that a sample of a size *p *corresponds to *p *events of the fast network, with the state at every fast event corresponding to one data point in the sample. The t-statistic for a 2 sample t-test, assuming unequal sample sizes, N_1 _and N_2_, and unequal variances, is given by

tj=ajs¯|1−ajs¯|2σj2|1N1+σj2|2N2∀ j∈Rs.
 MathType@MTEF@5@5@+=feaafiart1ev1aaatCvAUfKttLearuWrP9MDH5MBPbIqV92AaeXatLxBI9gBaebbnrfifHhDYfgasaacH8akY=wiFfYdH8Gipec8Eeeu0xXdbba9frFj0=OqFfea0dXdd9vqai=hGuQ8kuc9pgc9s8qqaq=dirpe0xb9q8qiLsFr0=vr0=vr0dc8meaabaqaciaacaGaaeqabaqabeGadaaakeaaieWacqWF0baDdaWgaaWcbaGaemOAaOgabeaakiabg2da9maalaaabaWaaqGaaeaadaqdaaqaaGqaaiab+fgaHnaaDaaaleaacqGFQbGAaeaacqGFZbWCaaaaaaGccaGLiWoadaWgaaWcbaGaeGymaedabeaakiabgkHiTmaaeiaabaWaa0aaaeaacqGFHbqydaqhaaWcbaGae4NAaOgabaGae43CamhaaaaaaOGaayjcSdWaaSbaaSqaaiabikdaYaqabaaakeaadaGcaaqaamaalaaabaWaaqGaaeaaiiGacqqFdpWCdaqhaaWcbaGae4NAaOgabaGaeGOmaidaaaGccaGLiWoadaWgaaWcbaGaeGymaedabeaaaOqaaiab+5eaonaaBaaaleaacqaIXaqmaeqaaaaakiabgUcaRmaalaaabaWaaqGaaeaacqqFdpWCdaqhaaWcbaGae4NAaOgabaGaeGOmaidaaaGccaGLiWoadaWgaaWcbaGaeGOmaidabeaaaOqaaiab+5eaonaaBaaaleaacqaIYaGmaeqaaaaaaeqaaaaakiabgcGiIiabbccaGiab+PgaQjabgIGiolab=jfasnaaCaaaleqabaGaem4CamhaaOGaeiOla4caaa@5BA4@

Here ajs¯|1
 MathType@MTEF@5@5@+=feaafiart1ev1aaatCvAUfKttLearuWrP9MDH5MBPbIqV92AaeXatLxBI9gBaebbnrfifHhDYfgasaacH8akY=wiFfYdH8Gipec8Eeeu0xXdbba9frFj0=OqFfea0dXdd9vqai=hGuQ8kuc9pgc9s8qqaq=dirpe0xb9q8qiLsFr0=vr0=vr0dc8meaabaqaciaacaGaaeqabaqabeGadaaakeaadaabcaqaamaanaaabaacbaGae8xyae2aa0baaSqaaiab=PgaQbqaaiab=nhaZbaaaaaakiaawIa7amaaBaaaleaacqaIXaqmaeqaaaaa@33BA@ and σj2|1
 MathType@MTEF@5@5@+=feaafiart1ev1aaatCvAUfKttLearuWrP9MDH5MBPbIqV92AaeXatLxBI9gBaebbnrfifHhDYfgasaacH8akY=wiFfYdH8Gipec8Eeeu0xXdbba9frFj0=OqFfea0dXdd9vqai=hGuQ8kuc9pgc9s8qqaq=dirpe0xb9q8qiLsFr0=vr0=vr0dc8meaabaqaciaacaGaaeqabaqabeGadaaakeaadaabcaqaaGGaciab=n8aZnaaDaaaleaaieaacqGFQbGAaeaacqGFYaGmaaaakiaawIa7amaaBaaaleaacqaIXaqmaeqaaaaa@33A8@ are, respectively, the mean and variance of the propensity ajs
 MathType@MTEF@5@5@+=feaafiart1ev1aaatCvAUfKttLearuWrP9MDH5MBPbIqV92AaeXatLxBI9gBaebbnrfifHhDYfgasaacH8akY=wiFfYdH8Gipec8Eeeu0xXdbba9frFj0=OqFfea0dXdd9vqai=hGuQ8kuc9pgc9s8qqaq=dirpe0xb9q8qiLsFr0=vr0=vr0dc8meaabaqaciaacaGaaeqabaqabeGadaaakeaaieaacqWFHbqydaqhaaWcbaGae8NAaOgabaGae83Camhaaaaa@30ED@ evaluated from N_1 _= (w - 1)·MCE_win _MC events in the first (w-1) simulation windows. ajs¯|2
 MathType@MTEF@5@5@+=feaafiart1ev1aaatCvAUfKttLearuWrP9MDH5MBPbIqV92AaeXatLxBI9gBaebbnrfifHhDYfgasaacH8akY=wiFfYdH8Gipec8Eeeu0xXdbba9frFj0=OqFfea0dXdd9vqai=hGuQ8kuc9pgc9s8qqaq=dirpe0xb9q8qiLsFr0=vr0=vr0dc8meaabaqaciaacaGaaeqabaqabeGadaaakeaadaabcaqaamaanaaabaacbaGae8xyae2aa0baaSqaaiab=PgaQbqaaiab=nhaZbaaaaaakiaawIa7amaaBaaaleaacqaIYaGmaeqaaaaa@33BC@ and σj2|2
 MathType@MTEF@5@5@+=feaafiart1ev1aaatCvAUfKttLearuWrP9MDH5MBPbIqV92AaeXatLxBI9gBaebbnrfifHhDYfgasaacH8akY=wiFfYdH8Gipec8Eeeu0xXdbba9frFj0=OqFfea0dXdd9vqai=hGuQ8kuc9pgc9s8qqaq=dirpe0xb9q8qiLsFr0=vr0=vr0dc8meaabaqaciaacaGaaeqabaqabeGadaaakeaadaabcaqaaGGaciab=n8aZnaaDaaaleaaieaacqGFQbGAaeaacqGFYaGmaaaakiaawIa7amaaBaaaleaacqaIYaGmaeqaaaaa@33AA@ are the mean and the variance of the propensity ajs
 MathType@MTEF@5@5@+=feaafiart1ev1aaatCvAUfKttLearuWrP9MDH5MBPbIqV92AaeXatLxBI9gBaebbnrfifHhDYfgasaacH8akY=wiFfYdH8Gipec8Eeeu0xXdbba9frFj0=OqFfea0dXdd9vqai=hGuQ8kuc9pgc9s8qqaq=dirpe0xb9q8qiLsFr0=vr0=vr0dc8meaabaqaciaacaGaaeqabaqabeGadaaakeaaieaacqWFHbqydaqhaaWcbaGae8NAaOgabaGae83Camhaaaaa@30ED@ from N_2 _= MCE_Win _events in the w^th ^(last) window. We define a window as a short stochastic simulation of the fast network, consisting of MCE_win _fast MC events. The slow-scale propensities are converged to a stationary value when

***-t***_α/2,υ _<***t***_*j*_, <***t***_α/2,υ, _for all j∈***R***^*s*^,

where ***t***_α/2,υ _is the value of the t-statistic for υ degrees of freedom and a significance level of α/2. We use a significance level, α = 0.05, in all the simulations shown in the results section. υ is evaluated as

υ=(σj2|1/N1+σj2|2/N2)2(σj2|1/N1)2/(N1−1)+(σj2|2/N2)2/(N2−1).
 MathType@MTEF@5@5@+=feaafiart1ev1aaatCvAUfKttLearuWrP9MDH5MBPbIqV92AaeXatLxBI9gBaebbnrfifHhDYfgasaacH8akY=wiFfYdH8Gipec8Eeeu0xXdbba9frFj0=OqFfea0dXdd9vqai=hGuQ8kuc9pgc9s8qqaq=dirpe0xb9q8qiLsFr0=vr0=vr0dc8meaabaqaciaacaGaaeqabaqabeGadaaakeaaiiGacqWFfpqDcqGH9aqpdaWcaaqaamaabmaabaWaaSGbaeaadaabcaqaaiab=n8aZnaaDaaaleaaieaacqGFQbGAaeaacqaIYaGmaaaakiaawIa7amaaBaaaleaacqaIXaqmaeqaaaGcbaGae4Nta40aaSbaaSqaaiabigdaXaqabaaaaOGaey4kaSYaaSGbaeaadaabcaqaaiab=n8aZnaaDaaaleaacqGFQbGAaeaacqaIYaGmaaaakiaawIa7amaaBaaaleaacqaIYaGmaeqaaaGcbaGae4Nta40aaSbaaSqaaiabikdaYaqabaaaaaGccaGLOaGaayzkaaWaaWbaaSqabeaacqaIYaGmaaaakeaadaWcgaqaamaabmaabaWaaSGbaeaadaabcaqaaiab=n8aZnaaDaaaleaacqGFQbGAaeaacqaIYaGmaaaakiaawIa7amaaBaaaleaacqaIXaqmaeqaaaGcbaGae4Nta40aaSbaaSqaaiabigdaXaqabaaaaaGccaGLOaGaayzkaaWaaWbaaSqabeaacqaIYaGmaaaakeaacqGGOaakcqGFobGtdaWgaaWcbaGaeGymaedabeaakiabgkHiTiabigdaXiabcMcaPaaacqGHRaWkdaWcgaqaamaabmaabaWaaSGbaeaadaabcaqaaiab=n8aZnaaDaaaleaacqGFQbGAaeaacqaIYaGmaaaakiaawIa7amaaBaaaleaacqaIYaGmaeqaaaGcbaGae4Nta40aaSbaaSqaaiabikdaYaqabaaaaaGccaGLOaGaayzkaaWaaWbaaSqabeaacqaIYaGmaaaakeaacqGGOaakcqGFobGtdaWgaaWcbaGaeGOmaidabeaakiabgkHiTiabigdaXiabcMcaPaaaaaGaeiOla4caaa@6B93@

To simplify the implementation of the above test and to avoid the recurring evaluation of the t_α/2,υ_, we assume that υ > 40, and hence fix the critical value at ***t***_α/2,υ _≅ 1.96. Since ***t***_α/2,υ _increases with decreasing υ, using ***t***_α/2,υ _≅ 1.96 in cases where υ < 40 imposes a more stringent criterion on the relaxation, and hence can be used safely.

The accuracy of the multiscale method depends on the convergence of the slow-scale propensities. For instance, if the QE PDF is bimodal, then it is crucial that both the branches be sufficiently sampled in evaluating the slow-scale propensities. Also, it is possible that by pure chance, the MCE_win _fast events in the first relaxation window do not alter the populations of any fast species participating in the slow network. So the t-test might falsely indicate convergence of slow-scale propensities as a result of inadequate sampling. To avoid such a situation, we suggest that the window length accounts for the size of, and the separation of scales within, the fast network, to ensure that all the fast reactions are adequately sampled.

We propose that the relaxation time, computed via CSP, should be used in conjunction with the relaxation time estimated via the statistical one to ensure sufficient sampling of QE state space. In general, we find good agreement between the statistical and CSP relaxation methods as detailed below in several numerical examples. In general, one should relax the system for the longest of the two times estimated by the CSP and the statistical criterion. Convergence of bistable systems is interesting (and more complex) as discussed in the results section.

### The Hybrid Multiscale Monte Carlo (HyMSMC) Method

The new relaxation criterion discussed above enhances the efficiency of the original MSMC method. There are two other areas that can be exploited to further accelerate the method: one is to execute multiple firings at each microscopic time step and the other is to reduce the number of evaluations of the QE PDF. One could reuse the same QE PDF for a few consecutive coarse (macroscopic) time steps, assuming that the occurrence of the slow events does not significantly alter this PDF. This is reminiscent of the τ-leap (TL) methods [6-10], where the leap condition allows one to perform multiple firings of the reactions in a pre-selected leap time. The inherent assumption of the TL methods is that the propensities remain unchanged in the leap time, and hence one can approximate the number of firings in the reaction channels using a Poisson [6,10] or a binomial random variable [7,8]. While maintaining the same QE PDF for a few coarse (macroscopic) time steps can potentially result in substantial computational savings, we need a criterion to indicate how long one could maintain the same PDF without sacrificing accuracy. We address this question by using the framework of the hybrid SSA-TL solver [11] to systematically coarse grain the macroscopic solver in time. Since leaping the slow reactions significantly moves the fast network away from its QE, we also propose the hybrid SSA-TL method [11] as the microsolver to speedup the relaxation of the fast network at every coarse time step.

The τ-leap solver [6-8], which temporally coarse-grains the exact SSA, works well only in the limit of large population [7,8,11]. At low population of the reactant species, there is a risk of observing negative populations due to the unbounded nature of the Poisson random variable. The binomial τ-leap methods [7,8] eliminate this problem, but are not as accurate and efficient at low populations. Recently, the hybrid SSA-TL method was introduced by Cao et al. [11], primarily, to reduce the possibility of negative population of the Poisson τ-leap solver. A similar hybrid solver, the partitioned leaping method [12], which combines the next reaction method [5] and the Poisson τ-leap method [6], was introduced by Harris and Clancy. We incorporate the hybrid SSA-TL solver [11] into the MSMC method as the macrosolver and the microsolver. This practically eliminates the likelihood of negative populations while using the explicit Poisson TL method. We call this method the hybrid multiscale Monte Carlo (HyMSMC) method.

The overall concept of HyMSMC is depicted in Figure 1. Integration of the hybrid solver with the multiscale framework begins with classification of the fast (and the slow) reactions into SSA reactions and TL reactions subset, RSSAf
 MathType@MTEF@5@5@+=feaafiart1ev1aaatCvAUfKttLearuWrP9MDH5MBPbIqV92AaeXatLxBI9gBaebbnrfifHhDYfgasaacH8akY=wiFfYdH8Gipec8Eeeu0xXdbba9frFj0=OqFfea0dXdd9vqai=hGuQ8kuc9pgc9s8qqaq=dirpe0xb9q8qiLsFr0=vr0=vr0dc8meaabaqaciaacaGaaeqabaqabeGadaaakeaaieWacqWFsbGudaqhaaWcbaGaem4uamLaem4uamLaemyqaeeabaGaemOzaygaaaaa@32CC@ and RTLf
 MathType@MTEF@5@5@+=feaafiart1ev1aaatCvAUfKttLearuWrP9MDH5MBPbIqV92AaeXatLxBI9gBaebbnrfifHhDYfgasaacH8akY=wiFfYdH8Gipec8Eeeu0xXdbba9frFj0=OqFfea0dXdd9vqai=hGuQ8kuc9pgc9s8qqaq=dirpe0xb9q8qiLsFr0=vr0=vr0dc8meaabaqaciaacaGaaeqabaqabeGadaaakeaaieWacqWFsbGudaqhaaWcbaGaemivaqLaemitaWeabaGaemOzaygaaaaa@31B5@ and (RSSAs
 MathType@MTEF@5@5@+=feaafiart1ev1aaatCvAUfKttLearuWrP9MDH5MBPbIqV92AaeXatLxBI9gBaebbnrfifHhDYfgasaacH8akY=wiFfYdH8Gipec8Eeeu0xXdbba9frFj0=OqFfea0dXdd9vqai=hGuQ8kuc9pgc9s8qqaq=dirpe0xb9q8qiLsFr0=vr0=vr0dc8meaabaqaciaacaGaaeqabaqabeGadaaakeaaieWacqWFsbGudaqhaaWcbaGaem4uamLaem4uamLaemyqaeeabaGaem4Camhaaaaa@32E6@ and RTLs
 MathType@MTEF@5@5@+=feaafiart1ev1aaatCvAUfKttLearuWrP9MDH5MBPbIqV92AaeXatLxBI9gBaebbnrfifHhDYfgasaacH8akY=wiFfYdH8Gipec8Eeeu0xXdbba9frFj0=OqFfea0dXdd9vqai=hGuQ8kuc9pgc9s8qqaq=dirpe0xb9q8qiLsFr0=vr0=vr0dc8meaabaqaciaacaGaaeqabaqabeGadaaakeaaieWacqWFsbGudaqhaaWcbaGaemivaqLaemitaWeabaGaem4Camhaaaaa@31CF@), respectively. All reactions whose reactant(s) population is less than some critical population, X_crit_, are defined as SSA reactions [11]. We have successfully used X_crit _= 10 in all simulations to obtain accurate results. In principle, the cost and accuracy of the hybrid solver increases with X_crit_, with the method reducing to SSA for X_crit _→ ∞, and to a Poisson TL for X_crit _= 0. Generating Poisson random numbers is more expensive than generating uniform random numbers. As a result, there is a X_crit _value below which the hybrid solver is more expensive than the SSA. This computational overhead of the hybrid solver should be considered in deciding the optimum X_crit _that maximizes the efficiency of the solution.

#### a. Hybrid Solver as the Microscopic Solver

The objective of using the hybrid solver as the microsolver is to accelerate the relaxation of the fast network via a coarser sampling of the QE state space of the fast species. Only one reaction from the SSA reaction group is executed. The average elapsed time until the occurrence of the next fast reaction belonging to RfSSA
 MathType@MTEF@5@5@+=feaafiart1ev1aaatCvAUfKttLearuWrP9MDH5MBPbIqV92AaeXatLxBI9gBaebbnrfifHhDYfgasaacH8akY=wiFfYdH8Gipec8Eeeu0xXdbba9frFj0=OqFfea0dXdd9vqai=hGuQ8kuc9pgc9s8qqaq=dirpe0xb9q8qiLsFr0=vr0=vr0dc8meaabaqaciaacaGaaeqabaqabeGadaaakeaaieWacqWFsbGudaqhaaWcbaGaemOzaygabaGaem4uamLaem4uamLaemyqaeeaaaaa@32CC@ is evaluated as [11]

τSSAf=−ln⁡(ξ1)∑j∈RSSAfajf.
 MathType@MTEF@5@5@+=feaafiart1ev1aaatCvAUfKttLearuWrP9MDH5MBPbIqV92AaeXatLxBI9gBaebbnrfifHhDYfgasaacH8akY=wiFfYdH8Gipec8Eeeu0xXdbba9frFj0=OqFfea0dXdd9vqai=hGuQ8kuc9pgc9s8qqaq=dirpe0xb9q8qiLsFr0=vr0=vr0dc8meaabaqaciaacaGaaeqabaqabeGadaaakeaaiiGacqWFepaDdaqhaaWcbaacbaGae43uamLae43uamLae4xqaeeabaGae4NzaygaaOGaeyypa0JaeyOeI0YaaSaaaeaacyGGSbaBcqGGUbGBcqGGOaakcqWF+oaEdaWgaaWcbaGaeGymaedabeaakiabcMcaPaqaamaaqafabaGae4xyae2aa0baaSqaaiab+PgaQbqaaiab+zgaMbaaaeaacqGFQbGAcqGHiiIZieWacqqFsbGudaqhaaadbaacbiGaeW3uamLaeW3uamLaeWxqaeeabaGaeWNzaygaaaWcbeqdcqGHris5aaaakiabc6caUaaa@4CD7@

The hybrid solver executes multiple firings of the TL reactions in a leap. The leap time is estimated using a leap criterion that imposes an upper bound on the mean and the variance of the change in the propensity in this leap time [28]. To evaluate τTLf
 MathType@MTEF@5@5@+=feaafiart1ev1aaatCvAUfKttLearuWrP9MDH5MBPbIqV92AaeXatLxBI9gBaebbnrfifHhDYfgasaacH8akY=wiFfYdH8Gipec8Eeeu0xXdbba9frFj0=OqFfea0dXdd9vqai=hGuQ8kuc9pgc9s8qqaq=dirpe0xb9q8qiLsFr0=vr0=vr0dc8meaabaqaciaacaGaaeqabaqabeGadaaakeaaiiGacqWFepaDdaqhaaWcbaacbaGae4hvaqLae4htaWeabaGae4Nzaygaaaaa@3246@, we use the r-criterion (related to the stability of the stochastic model) given by [7]

τTLf=min⁡i∈Sf{rxif∑j∈RTLf;υijf<0−υijfajf(xf)}.
 MathType@MTEF@5@5@+=feaafiart1ev1aaatCvAUfKttLearuWrP9MDH5MBPbIqV92AaeXatLxBI9gBaebbnrfifHhDYfgasaacH8akY=wiFfYdH8Gipec8Eeeu0xXdbba9frFj0=OqFfea0dXdd9vqai=hGuQ8kuc9pgc9s8qqaq=dirpe0xb9q8qiLsFr0=vr0=vr0dc8meaabaqaciaacaGaaeqabaqabeGadaaakeaaiiGacqWFepaDdaqhaaWcbaacbaGae4hvaqLae4htaWeabaGae4NzaygaaOGaeyypa0ZaaCbeaeaacyGGTbqBcqGGPbqAcqGGUbGBaSqaaiab+LgaPjabgIGioJqadiab9nfatnaaCaaameqabaGaemOzaygaaaWcbeaakmaacmaabaWaaSaaaeaacqGFYbGCcqGF4baEdaqhaaWcbaGae4xAaKgabaGae4NzaygaaaGcbaWaaabuaeaacqGHsislcqWFfpqDdaqhaaWcbaGae4xAaKMae4NAaOgabaGae4NzaygaaOGae4xyae2aa0baaSqaaiab+PgaQbqaaiab+zgaMbaakmaabmaabaacbeGaeWhEaG3aaWbaaSqabeaacqaFMbGzaaaakiaawIcacaGLPaaaaSqaaiab+PgaQjabgIGiolab9jfasnaaDaaameaacqWGubavcqWGmbataeaacqWGMbGzaaWccqGG7aWocqWFfpqDdaqhaaadbaGae4xAaKMae4NAaOgabaGae4NzaygaaSGaeyipaWJaeGimaadabeqdcqGHris5aaaaaOGaay5Eaiaaw2haaiabc6caUaaa@68C2@

The time increment τ^f ^is chosen as

τf=min⁡{τTLf,τSSAf}
 MathType@MTEF@5@5@+=feaafiart1ev1aaatCvAUfKttLearuWrP9MDH5MBPbIqV92AaeXatLxBI9gBaebbnrfifHhDYfgasaacH8akY=wiFfYdH8Gipec8Eeeu0xXdbba9frFj0=OqFfea0dXdd9vqai=hGuQ8kuc9pgc9s8qqaq=dirpe0xb9q8qiLsFr0=vr0=vr0dc8meaabaqaciaacaGaaeqabaqabeGadaaakeaaiiGacqWFepaDdaahaaWcbeqaaGqaaiab+zgaMbaakiabg2da9iGbc2gaTjabcMgaPjabc6gaUnaacmaabaGae8hXdq3aa0baaSqaaiab+rfaujab+Xeambqaaiab+zgaMbaakiabcYcaSiab=r8a0naaDaaaleaacqGFtbWucqGFtbWucqGFbbqqaeaacqGFMbGzaaaakiaawUhacaGL9baaaaa@4471@

If τTLf≥τSSAf
 MathType@MTEF@5@5@+=feaafiart1ev1aaatCvAUfKttLearuWrP9MDH5MBPbIqV92AaeXatLxBI9gBaebbnrfifHhDYfgasaacH8akY=wiFfYdH8Gipec8Eeeu0xXdbba9frFj0=OqFfea0dXdd9vqai=hGuQ8kuc9pgc9s8qqaq=dirpe0xb9q8qiLsFr0=vr0=vr0dc8meaabaqaciaacaGaaeqabaqabeGadaaakeaaiiGacqWFepaDdaqhaaWcbaacbaGae4hvaqLae4htaWeabaGae4NzaygaaOGaeyyzImRae8hXdq3aa0baaSqaaiab+nfatjab+nfatjab+feabbqaaiab+zgaMbaaaaa@3AAD@, we sample a single fast reaction (kjf
 MathType@MTEF@5@5@+=feaafiart1ev1aaatCvAUfKttLearuWrP9MDH5MBPbIqV92AaeXatLxBI9gBaebbnrfifHhDYfgasaacH8akY=wiFfYdH8Gipec8Eeeu0xXdbba9frFj0=OqFfea0dXdd9vqai=hGuQ8kuc9pgc9s8qqaq=dirpe0xb9q8qiLsFr0=vr0=vr0dc8meaabaqaciaacaGaaeqabaqabeGadaaakeaaieaacqWFRbWAdaqhaaWcbaGae8NAaOgabaGae8Nzaygaaaaa@30E7@ = 1) from the subset RSSAf
 MathType@MTEF@5@5@+=feaafiart1ev1aaatCvAUfKttLearuWrP9MDH5MBPbIqV92AaeXatLxBI9gBaebbnrfifHhDYfgasaacH8akY=wiFfYdH8Gipec8Eeeu0xXdbba9frFj0=OqFfea0dXdd9vqai=hGuQ8kuc9pgc9s8qqaq=dirpe0xb9q8qiLsFr0=vr0=vr0dc8meaabaqaciaacaGaaeqabaqabeGadaaakeaaieWacqWFsbGudaqhaaWcbaGaem4uamLaem4uamLaemyqaeeabaGaemOzaygaaaaa@32CC@ such that j is the smallest integer satisfying

∑i=1jaif≥ξ2⋅∑i∈RSSAfaif.
 MathType@MTEF@5@5@+=feaafiart1ev1aaatCvAUfKttLearuWrP9MDH5MBPbIqV92AaeXatLxBI9gBaebbnrfifHhDYfgasaacH8akY=wiFfYdH8Gipec8Eeeu0xXdbba9frFj0=OqFfea0dXdd9vqai=hGuQ8kuc9pgc9s8qqaq=dirpe0xb9q8qiLsFr0=vr0=vr0dc8meaabaqaciaacaGaaeqabaqabeGadaaakeaadaaeWbqaaGqaaiab=fgaHnaaDaaaleaacqWFPbqAaeaacqWFMbGzaaGccqGHLjYSiiGacqGF+oaEdaWgaaWcbaGaeGOmaidabeaaaeaacqWFPbqAcqGH9aqpcqaIXaqmaeaacqWFQbGAa0GaeyyeIuoakiabgwSixpaaqafabaGae8xyae2aa0baaSqaaiab=LgaPbqaaiab=zgaMbaaaeaacqWFPbqAcqGHiiIZieWacqqFsbGudaqhaaadbaGaem4uamLaem4uamLaemyqaeeabaGaemOzaygaaaWcbeqdcqGHris5aOGaeiOla4caaa@4EE0@

Here ξ_1 _and ξ_2 _are uniform random numbers, sampled from the unit interval (0,1]. The number of firings, kjf
 MathType@MTEF@5@5@+=feaafiart1ev1aaatCvAUfKttLearuWrP9MDH5MBPbIqV92AaeXatLxBI9gBaebbnrfifHhDYfgasaacH8akY=wiFfYdH8Gipec8Eeeu0xXdbba9frFj0=OqFfea0dXdd9vqai=hGuQ8kuc9pgc9s8qqaq=dirpe0xb9q8qiLsFr0=vr0=vr0dc8meaabaqaciaacaGaaeqabaqabeGadaaakeaaieaacqWFRbWAdaqhaaWcbaGae8NAaOgabaGae8Nzaygaaaaa@30E7@ of a TL reaction Rjf
 MathType@MTEF@5@5@+=feaafiart1ev1aaatCvAUfKttLearuWrP9MDH5MBPbIqV92AaeXatLxBI9gBaebbnrfifHhDYfgasaacH8akY=wiFfYdH8Gipec8Eeeu0xXdbba9frFj0=OqFfea0dXdd9vqai=hGuQ8kuc9pgc9s8qqaq=dirpe0xb9q8qiLsFr0=vr0=vr0dc8meaabaqaciaacaGaaeqabaqabeGadaaakeaaieaacqWFsbGudaqhaaWcbaGae8NAaOgabaGae8Nzaygaaaaa@30B5@ ∈ RTLf
 MathType@MTEF@5@5@+=feaafiart1ev1aaatCvAUfKttLearuWrP9MDH5MBPbIqV92AaeXatLxBI9gBaebbnrfifHhDYfgasaacH8akY=wiFfYdH8Gipec8Eeeu0xXdbba9frFj0=OqFfea0dXdd9vqai=hGuQ8kuc9pgc9s8qqaq=dirpe0xb9q8qiLsFr0=vr0=vr0dc8meaabaqaciaacaGaaeqabaqabeGadaaakeaaieWacqWFsbGudaqhaaWcbaGaemivaqLaemitaWeabaGaemOzaygaaaaa@31B5@is sampled from a Poisson distribution with mean ajfτf
 MathType@MTEF@5@5@+=feaafiart1ev1aaatCvAUfKttLearuWrP9MDH5MBPbIqV92AaeXatLxBI9gBaebbnrfifHhDYfgasaacH8akY=wiFfYdH8Gipec8Eeeu0xXdbba9frFj0=OqFfea0dXdd9vqai=hGuQ8kuc9pgc9s8qqaq=dirpe0xb9q8qiLsFr0=vr0=vr0dc8meaabaqaciaacaGaaeqabaqabeGadaaakeaaieaacqWFHbqydaqhaaWcbaGae8NAaOgabaGae8NzaygaaGGacOGae4hXdq3aaWbaaSqabeaacqWFMbGzaaaaaa@3426@

kjf=P(ajfτf),for allj∈RTLf.
 MathType@MTEF@5@5@+=feaafiart1ev1aaatCvAUfKttLearuWrP9MDH5MBPbIqV92AaeXatLxBI9gBaebbnrfifHhDYfgasaacH8akY=wiFfYdH8Gipec8Eeeu0xXdbba9frFj0=OqFfea0dXdd9vqai=hGuQ8kuc9pgc9s8qqaq=dirpe0xb9q8qiLsFr0=vr0=vr0dc8meaabaqaciaacaGaaeqabaqabeGadaaakeaaieaacqWFRbWAdaqhaaWcbaGae8NAaOgabaGae8NzaygaaOGaeyypa0dcbmGae4huaa1aaeWaaeaacqWFHbqydaqhaaWcbaGae8NAaOgabaGae8NzaygaaGGacOGae0hXdq3aaWbaaSqabeaacqWFMbGzaaaakiaawIcacaGLPaaacqGGSaalcqWFMbGzcqWFVbWBcqWFYbGCcqqGGaaicqWFHbqycqWFSbaBcqWFSbaBcqWFGaaicqWGQbGAcqGHiiIZcqGFsbGudaqhaaWcbaGaemivaqLaemitaWeabaGaemOzaygaaOGaeiOla4caaa@4F7F@

If τTLf<τSSAf
 MathType@MTEF@5@5@+=feaafiart1ev1aaatCvAUfKttLearuWrP9MDH5MBPbIqV92AaeXatLxBI9gBaebbnrfifHhDYfgasaacH8akY=wiFfYdH8Gipec8Eeeu0xXdbba9frFj0=OqFfea0dXdd9vqai=hGuQ8kuc9pgc9s8qqaq=dirpe0xb9q8qiLsFr0=vr0=vr0dc8meaabaqaciaacaGaaeqabaqabeGadaaakeaaiiGacqWFepaDdaqhaaWcbaacbaGae4hvaqLae4htaWeabaGae4NzaygaaOGaeyipaWJae8hXdq3aa0baaSqaaiab+nfatjab+nfatjab+feabbqaaiab+zgaMbaaaaa@39EB@ then only the TL reactions are fired according to Eq. (18).

#### b. Hybrid Solver as the Macroscopic Solver

Employing the hybrid solver as the macroscopic solver to evolve the slow network replaces the instantaneous value of propensities in the above Eqs. with the slow-scale propensities. For completeness, the time increment used to evolve the slow network is given by

τs=min⁡{τTLs,τSSAs}
 MathType@MTEF@5@5@+=feaafiart1ev1aaatCvAUfKttLearuWrP9MDH5MBPbIqV92AaeXatLxBI9gBaebbnrfifHhDYfgasaacH8akY=wiFfYdH8Gipec8Eeeu0xXdbba9frFj0=OqFfea0dXdd9vqai=hGuQ8kuc9pgc9s8qqaq=dirpe0xb9q8qiLsFr0=vr0=vr0dc8meaabaqaciaacaGaaeqabaqabeGadaaakeaaiiGacqWFepaDdaahaaWcbeqaaGqaaiab+nhaZbaakiabg2da9iGbc2gaTjabcMgaPjabc6gaUnaacmaabaGae8hXdq3aa0baaSqaaiab+rfaujab+Xeambqaaiab+nhaZbaakiabcYcaSiab=r8a0naaDaaaleaacqGFtbWucqGFtbWucqGFbbqqaeaacqGFZbWCaaaakiaawUhacaGL9baaaaa@44BF@

where

τSSAs=−ln⁡(ξ1)∑j∈RSSAsajs
 MathType@MTEF@5@5@+=feaafiart1ev1aaatCvAUfKttLearuWrP9MDH5MBPbIqV92AaeXatLxBI9gBaebbnrfifHhDYfgasaacH8akY=wiFfYdH8Gipec8Eeeu0xXdbba9frFj0=OqFfea0dXdd9vqai=hGuQ8kuc9pgc9s8qqaq=dirpe0xb9q8qiLsFr0=vr0=vr0dc8meaabaqaciaacaGaaeqabaqabeGadaaakeaacqaHepaDdaqhaaWcbaacbaGae83uamLae83uamLae8xqaeeabaGae83CamhaaOGaeyypa0JaeyOeI0YaaSaaaeaacyGGSbaBcqGGUbGBdaqadaqaaGGaciab+57a4naaBaaaleaacqaIXaqmaeqaaaGccaGLOaGaayzkaaaabaWaaabuaeaacqWFHbqydaqhaaWcbaGae8NAaOgabaGae83Camhaaaqaaiab=PgaQjabgIGioJqadiab9jfasnaaDaaameaacqWGtbWucqWGtbWucqWGbbqqaeaacqWGZbWCaaaaleqaniabggHiLdaaaaaa@4C2B@

τTLs=min⁡i∈S{rxi|∑j∈RTLs;υijs<0−υijsajs¯|}.
 MathType@MTEF@5@5@+=feaafiart1ev1aaatCvAUfKttLearuWrP9MDH5MBPbIqV92AaeXatLxBI9gBaebbnrfifHhDYfgasaacH8akY=wiFfYdH8Gipec8Eeeu0xXdbba9frFj0=OqFfea0dXdd9vqai=hGuQ8kuc9pgc9s8qqaq=dirpe0xb9q8qiLsFr0=vr0=vr0dc8meaabaqaciaacaGaaeqabaqabeGadaaakeaaiiGacqWFepaDdaqhaaWcbaacbaGae4hvaqLae4htaWeabaGae43CamhaaOGaeyypa0ZaaCbeaeaacyGGTbqBcqGGPbqAcqGGUbGBaSqaaiab+LgaPjabgIGioJqadiab9nfatbqabaGcdaGadaqaamaalaaabaGae4NCaiNae4hEaG3aaSbaaSqaaiab+LgaPbqabaaakeaadaabdaqaamaaqafabaGaeyOeI0Iae8xXdu3aa0baaSqaaiab+LgaPjab+PgaQbqaaiab+nhaZbaakmaanaaabaGae4xyae2aa0baaSqaaiab+PgaQbqaaiab+nhaZbaaaaaabaGae4NAaOMaeyicI4Sae0Nuai1aa0baaWqaaiabdsfaujabdYeambqaaiabdohaZbaaliabcUda7iab=v8a1naaDaaameaacqGFPbqAcqGFQbGAaeaacqGFZbWCaaWccqGH8aapcqaIWaamaeqaniabggHiLdaakiaawEa7caGLiWoaaaaacaGL7bGaayzFaaGaeiOla4caaa@64F9@

If τSSAs≤τTLs
 MathType@MTEF@5@5@+=feaafiart1ev1aaatCvAUfKttLearuWrP9MDH5MBPbIqV92AaeXatLxBI9gBaebbnrfifHhDYfgasaacH8akY=wiFfYdH8Gipec8Eeeu0xXdbba9frFj0=OqFfea0dXdd9vqai=hGuQ8kuc9pgc9s8qqaq=dirpe0xb9q8qiLsFr0=vr0=vr0dc8meaabaqaciaacaGaaeqabaqabeGadaaakeaaiiGacqWFepaDdaqhaaWcbaacbaGae43uamLae43uamLae4xqaeeabaGae43CamhaaOGaeyizImQae8hXdq3aa0baaSqaaiab+rfaujab+Xeambqaaiab+nhaZbaaaaa@3AD0@, we sample a single reaction (kjs
 MathType@MTEF@5@5@+=feaafiart1ev1aaatCvAUfKttLearuWrP9MDH5MBPbIqV92AaeXatLxBI9gBaebbnrfifHhDYfgasaacH8akY=wiFfYdH8Gipec8Eeeu0xXdbba9frFj0=OqFfea0dXdd9vqai=hGuQ8kuc9pgc9s8qqaq=dirpe0xb9q8qiLsFr0=vr0=vr0dc8meaabaqaciaacaGaaeqabaqabeGadaaakeaaieaacqWFRbWAdaqhaaWcbaGae8NAaOgabaGae83Camhaaaaa@3101@ = 1) from the subset RSSAs
 MathType@MTEF@5@5@+=feaafiart1ev1aaatCvAUfKttLearuWrP9MDH5MBPbIqV92AaeXatLxBI9gBaebbnrfifHhDYfgasaacH8akY=wiFfYdH8Gipec8Eeeu0xXdbba9frFj0=OqFfea0dXdd9vqai=hGuQ8kuc9pgc9s8qqaq=dirpe0xb9q8qiLsFr0=vr0=vr0dc8meaabaqaciaacaGaaeqabaqabeGadaaakeaaieWacqWFsbGudaqhaaWcbaGaem4uamLaem4uamLaemyqaeeabaGaem4Camhaaaaa@32E6@, such that j is the smallest integer satisfying

∑i=1jais¯≥ξ2⋅∑i∈RSSAsais¯.
 MathType@MTEF@5@5@+=feaafiart1ev1aaatCvAUfKttLearuWrP9MDH5MBPbIqV92AaeXatLxBI9gBaebbnrfifHhDYfgasaacH8akY=wiFfYdH8Gipec8Eeeu0xXdbba9frFj0=OqFfea0dXdd9vqai=hGuQ8kuc9pgc9s8qqaq=dirpe0xb9q8qiLsFr0=vr0=vr0dc8meaabaqaciaacaGaaeqabaqabeGadaaakeaadaaeWbqaamaanaaabaacbaGae8xyae2aa0baaSqaaiab=LgaPbqaaiab=nhaZbaaaaGccqGHLjYSiiGacqGF+oaEdaWgaaWcbaGaeGOmaidabeaakiabgwSixpaaqafabaWaa0aaaeaacqWFHbqydaqhaaWcbaGae8xAaKgabaGae83CamhaaaaaaeaacqWFPbqAcqGHiiIZcqWGsbGudaqhaaadbaacbmGae03uamLae03uamLae0xqaeeabaGae03CamhaaaWcbeqdcqGHris5aaWcbaGae8xAaKMaeyypa0JaeGymaedabaGae8NAaOganiabggHiLdGccqGGUaGlaaa@4F49@

The number of firings, kjs
 MathType@MTEF@5@5@+=feaafiart1ev1aaatCvAUfKttLearuWrP9MDH5MBPbIqV92AaeXatLxBI9gBaebbnrfifHhDYfgasaacH8akY=wiFfYdH8Gipec8Eeeu0xXdbba9frFj0=OqFfea0dXdd9vqai=hGuQ8kuc9pgc9s8qqaq=dirpe0xb9q8qiLsFr0=vr0=vr0dc8meaabaqaciaacaGaaeqabaqabeGadaaakeaaieaacqWFRbWAdaqhaaWcbaGae8NAaOgabaGae83Camhaaaaa@3101@, of a TL reaction Rjs
 MathType@MTEF@5@5@+=feaafiart1ev1aaatCvAUfKttLearuWrP9MDH5MBPbIqV92AaeXatLxBI9gBaebbnrfifHhDYfgasaacH8akY=wiFfYdH8Gipec8Eeeu0xXdbba9frFj0=OqFfea0dXdd9vqai=hGuQ8kuc9pgc9s8qqaq=dirpe0xb9q8qiLsFr0=vr0=vr0dc8meaabaqaciaacaGaaeqabaqabeGadaaakeaaieaacqWFsbGudaqhaaWcbaGae8NAaOgabaGae83Camhaaaaa@30CF@ ∈ RTLs
 MathType@MTEF@5@5@+=feaafiart1ev1aaatCvAUfKttLearuWrP9MDH5MBPbIqV92AaeXatLxBI9gBaebbnrfifHhDYfgasaacH8akY=wiFfYdH8Gipec8Eeeu0xXdbba9frFj0=OqFfea0dXdd9vqai=hGuQ8kuc9pgc9s8qqaq=dirpe0xb9q8qiLsFr0=vr0=vr0dc8meaabaqaciaacaGaaeqabaqabeGadaaakeaaieWacqWFsbGudaqhaaWcbaGaemivaqLaemitaWeabaGaem4Camhaaaaa@31CF@ is sampled from a Poisson distribution with mean ajs¯
 MathType@MTEF@5@5@+=feaafiart1ev1aaatCvAUfKttLearuWrP9MDH5MBPbIqV92AaeXatLxBI9gBaebbnrfifHhDYfgasaacH8akY=wiFfYdH8Gipec8Eeeu0xXdbba9frFj0=OqFfea0dXdd9vqai=hGuQ8kuc9pgc9s8qqaq=dirpe0xb9q8qiLsFr0=vr0=vr0dc8meaabaqaciaacaGaaeqabaqabeGadaaakeaadaqdaaqaaGqaaiab=fgaHnaaDaaaleaacqWFQbGAaeaacqWFZbWCaaaaaaaa@30FE@*τ*^s^

kjs=P(ajs¯τs),for allj∈RTLs.
 MathType@MTEF@5@5@+=feaafiart1ev1aaatCvAUfKttLearuWrP9MDH5MBPbIqV92AaeXatLxBI9gBaebbnrfifHhDYfgasaacH8akY=wiFfYdH8Gipec8Eeeu0xXdbba9frFj0=OqFfea0dXdd9vqai=hGuQ8kuc9pgc9s8qqaq=dirpe0xb9q8qiLsFr0=vr0=vr0dc8meaabaqaciaacaGaaeqabaqabeGadaaakeaaieaacqWFRbWAdaqhaaWcbaGae8NAaOgabaGae83CamhaaOGaeyypa0dcbmGae4huaa1aaeWaaeaadaqdaaqaaiab=fgaHnaaDaaaleaacqWFQbGAaeaacqWFZbWCaaaaaGGacOGae0hXdq3aaWbaaSqabeaacqWFZbWCaaaakiaawIcacaGLPaaacqGGSaalcqWFMbGzcqWFVbWBcqWFYbGCcqqGGaaicqWFHbqycqWFSbaBcqWFSbaBcqWFGaaicqWFQbGAcqGHiiIZcqGFsbGudaqhaaWcbaGae4hvaqLae4htaWeabaGae43CamhaaOGaeiOla4caaa@4FE5@

If τSSAs>τTLs
 MathType@MTEF@5@5@+=feaafiart1ev1aaatCvAUfKttLearuWrP9MDH5MBPbIqV92AaeXatLxBI9gBaebbnrfifHhDYfgasaacH8akY=wiFfYdH8Gipec8Eeeu0xXdbba9frFj0=OqFfea0dXdd9vqai=hGuQ8kuc9pgc9s8qqaq=dirpe0xb9q8qiLsFr0=vr0=vr0dc8meaabaqaciaacaGaaeqabaqabeGadaaakeaaiiGacqWFepaDdaqhaaWcbaacbaGae43uamLae43uamLae4xqaeeabaGae43CamhaaOGaeyOpa4Jae8hXdq3aa0baaSqaaiab+rfaujab+Xeambqaaiab+nhaZbaaaaa@3A23@, only the TL reactions are executed according to Eq. (23).

### Algorithm Implementation

1. Initialize the system state X(t = 0) at time t = 0 and the simulation parameters, such as the critical population X_crit_, the number of fast MC events, MCE_win_, for each relaxation window, the coarse graining factor, r, in the leap criterion, the partitioning criterion, and the significance level a of the statistical test used for the relaxation.

2. At the state **X**(t) = (**x**^**f**^,**x**^**s**^), evaluate the propensities of all the reactions in the

network. Partition the reaction network into a fast and a slow network, and the species into fast and slow species.

#### a. Microscopic Solver – Fast Network

3. Perform MCE_win _MC events of the fast network using the hybrid SSA-TL solver. In each event,

a. Identify the SSA and the TL subsets in the fast network, RSSAf
 MathType@MTEF@5@5@+=feaafiart1ev1aaatCvAUfKttLearuWrP9MDH5MBPbIqV92AaeXatLxBI9gBaebbnrfifHhDYfgasaacH8akY=wiFfYdH8Gipec8Eeeu0xXdbba9frFj0=OqFfea0dXdd9vqai=hGuQ8kuc9pgc9s8qqaq=dirpe0xb9q8qiLsFr0=vr0=vr0dc8meaabaqaciaacaGaaeqabaqabeGadaaakeaaieWacqWFsbGudaqhaaWcbaGaem4uamLaem4uamLaemyqaeeabaGaemOzaygaaaaa@32CC@ and RTLf
 MathType@MTEF@5@5@+=feaafiart1ev1aaatCvAUfKttLearuWrP9MDH5MBPbIqV92AaeXatLxBI9gBaebbnrfifHhDYfgasaacH8akY=wiFfYdH8Gipec8Eeeu0xXdbba9frFj0=OqFfea0dXdd9vqai=hGuQ8kuc9pgc9s8qqaq=dirpe0xb9q8qiLsFr0=vr0=vr0dc8meaabaqaciaacaGaaeqabaqabeGadaaakeaaieWacqWFsbGudaqhaaWcbaGaemivaqLaemitaWeabaGaemOzaygaaaaa@31B5@, respectively, based on the populations of the participating reactants;

b. Evaluate the time increment τ^f ^using (14)-(16);

c. Execute kjf
 MathType@MTEF@5@5@+=feaafiart1ev1aaatCvAUfKttLearuWrP9MDH5MBPbIqV92AaeXatLxBI9gBaebbnrfifHhDYfgasaacH8akY=wiFfYdH8Gipec8Eeeu0xXdbba9frFj0=OqFfea0dXdd9vqai=hGuQ8kuc9pgc9s8qqaq=dirpe0xb9q8qiLsFr0=vr0=vr0dc8meaabaqaciaacaGaaeqabaqabeGadaaakeaaieaacqWFRbWAdaqhaaWcbaGae8NAaOgabaGae8Nzaygaaaaa@30E7@ reactions, Rjf
 MathType@MTEF@5@5@+=feaafiart1ev1aaatCvAUfKttLearuWrP9MDH5MBPbIqV92AaeXatLxBI9gBaebbnrfifHhDYfgasaacH8akY=wiFfYdH8Gipec8Eeeu0xXdbba9frFj0=OqFfea0dXdd9vqai=hGuQ8kuc9pgc9s8qqaq=dirpe0xb9q8qiLsFr0=vr0=vr0dc8meaabaqaciaacaGaaeqabaqabeGadaaakeaaieaacqWFsbGudaqhaaWcbaGae8NAaOgabaGae8Nzaygaaaaa@30B5@ ∈ ***R***^*f *^using (17) and (18);

d. Update the state of the fast species, xif←xif+∑j∈Rfkjfνijf fori∈Sf
 MathType@MTEF@5@5@+=feaafiart1ev1aaatCvAUfKttLearuWrP9MDH5MBPbIqV92AaeXatLxBI9gBaebbnrfifHhDYfgasaacH8akY=wiFfYdH8Gipec8Eeeu0xXdbba9frFj0=OqFfea0dXdd9vqai=hGuQ8kuc9pgc9s8qqaq=dirpe0xb9q8qiLsFr0=vr0=vr0dc8meaabaqaciaacaGaaeqabaqabeGadaaakeaaieaacqWF4baEdaqhaaWcbaGae8xAaKgabaGae8NzaygaaOGaeyiKHWQae8hEaG3aa0baaSqaaiab=LgaPbqaaiab=zgaMbaakiabgUcaRmaaqafabaGae83AaS2aa0baaSqaaiab=PgaQbqaaiab=zgaMbaaiiGakiab+17aUnaaDaaaleaacqWFPbqAcqWFQbGAaeaacqWFMbGzaaGccqqGGaaiaSqaaiab=PgaQjabgIGioJqadiab9jfasnaaCaaameqabaacbiGaeWNzaygaaaWcbeqdcqGHris5aOGae8NzayMae83Ba8Mae8NCaiNae8hiaaIae8xAaKMaeyicI4Sae03uam1aaWbaaSqabeaacqWGMbGzaaaaaa@5558@

4. Evaluate the slow-scale propensities ajs¯
 MathType@MTEF@5@5@+=feaafiart1ev1aaatCvAUfKttLearuWrP9MDH5MBPbIqV92AaeXatLxBI9gBaebbnrfifHhDYfgasaacH8akY=wiFfYdH8Gipec8Eeeu0xXdbba9frFj0=OqFfea0dXdd9vqai=hGuQ8kuc9pgc9s8qqaq=dirpe0xb9q8qiLsFr0=vr0=vr0dc8meaabaqaciaacaGaaeqabaqabeGadaaakeaadaqdaaqaaGqaaiab=fgaHnaaDaaaleaacqWFQbGAaeaacqWFZbWCaaaaaaaa@30FE@(see Appendix, Eq. (4)).

5. If the convergence criterion (12) is satisfied and the time is longer than the relaxation time predicted by CSP, go to 6. Else, go to 3 and perform another MCE_win _fast events using the hybrid solver.

#### b. Macroscopic Solver – Slow Network

6. Evolve the slow network using the hybrid SSA-TL solver:

a. Identify the TL and the SSA reactions subsets RTLs
 MathType@MTEF@5@5@+=feaafiart1ev1aaatCvAUfKttLearuWrP9MDH5MBPbIqV92AaeXatLxBI9gBaebbnrfifHhDYfgasaacH8akY=wiFfYdH8Gipec8Eeeu0xXdbba9frFj0=OqFfea0dXdd9vqai=hGuQ8kuc9pgc9s8qqaq=dirpe0xb9q8qiLsFr0=vr0=vr0dc8meaabaqaciaacaGaaeqabaqabeGadaaakeaaieWacqWFsbGudaqhaaWcbaGaemivaqLaemitaWeabaGaem4Camhaaaaa@31CF@ and RTLs
 MathType@MTEF@5@5@+=feaafiart1ev1aaatCvAUfKttLearuWrP9MDH5MBPbIqV92AaeXatLxBI9gBaebbnrfifHhDYfgasaacH8akY=wiFfYdH8Gipec8Eeeu0xXdbba9frFj0=OqFfea0dXdd9vqai=hGuQ8kuc9pgc9s8qqaq=dirpe0xb9q8qiLsFr0=vr0=vr0dc8meaabaqaciaacaGaaeqabaqabeGadaaakeaaieWacqWFsbGudaqhaaWcbaGaemivaqLaemitaWeabaGaem4Camhaaaaa@31CF@ at the state (**x**^**f**^, **x**^**s**^);

b. Evaluate the time increment τ^s ^using Eqs. (19)-(21);

c. Evaluate the number of firing kjs
 MathType@MTEF@5@5@+=feaafiart1ev1aaatCvAUfKttLearuWrP9MDH5MBPbIqV92AaeXatLxBI9gBaebbnrfifHhDYfgasaacH8akY=wiFfYdH8Gipec8Eeeu0xXdbba9frFj0=OqFfea0dXdd9vqai=hGuQ8kuc9pgc9s8qqaq=dirpe0xb9q8qiLsFr0=vr0=vr0dc8meaabaqaciaacaGaaeqabaqabeGadaaakeaaieaacqWFRbWAdaqhaaWcbaGae8NAaOgabaGae83Camhaaaaa@3101@ of Rjs
 MathType@MTEF@5@5@+=feaafiart1ev1aaatCvAUfKttLearuWrP9MDH5MBPbIqV92AaeXatLxBI9gBaebbnrfifHhDYfgasaacH8akY=wiFfYdH8Gipec8Eeeu0xXdbba9frFj0=OqFfea0dXdd9vqai=hGuQ8kuc9pgc9s8qqaq=dirpe0xb9q8qiLsFr0=vr0=vr0dc8meaabaqaciaacaGaaeqabaqabeGadaaakeaaieaacqWFsbGudaqhaaWcbaGae8NAaOgabaGae83Camhaaaaa@30CF@, using Eqs. (22) and (23);

d. If any reaction Rjs
 MathType@MTEF@5@5@+=feaafiart1ev1aaatCvAUfKttLearuWrP9MDH5MBPbIqV92AaeXatLxBI9gBaebbnrfifHhDYfgasaacH8akY=wiFfYdH8Gipec8Eeeu0xXdbba9frFj0=OqFfea0dXdd9vqai=hGuQ8kuc9pgc9s8qqaq=dirpe0xb9q8qiLsFr0=vr0=vr0dc8meaabaqaciaacaGaaeqabaqabeGadaaakeaaieaacqWFsbGudaqhaaWcbaGae8NAaOgabaGae83Camhaaaaa@30CF@ ∈ ***R***^***s ***^cannot be fired kjs
 MathType@MTEF@5@5@+=feaafiart1ev1aaatCvAUfKttLearuWrP9MDH5MBPbIqV92AaeXatLxBI9gBaebbnrfifHhDYfgasaacH8akY=wiFfYdH8Gipec8Eeeu0xXdbba9frFj0=OqFfea0dXdd9vqai=hGuQ8kuc9pgc9s8qqaq=dirpe0xb9q8qiLsFr0=vr0=vr0dc8meaabaqaciaacaGaaeqabaqabeGadaaakeaaieaacqWFRbWAdaqhaaWcbaGae8NAaOgabaGae83Camhaaaaa@3101@ times, execute steps 3a-3d until state **X**^**f **^= **x**^**f **^allows kjs
 MathType@MTEF@5@5@+=feaafiart1ev1aaatCvAUfKttLearuWrP9MDH5MBPbIqV92AaeXatLxBI9gBaebbnrfifHhDYfgasaacH8akY=wiFfYdH8Gipec8Eeeu0xXdbba9frFj0=OqFfea0dXdd9vqai=hGuQ8kuc9pgc9s8qqaq=dirpe0xb9q8qiLsFr0=vr0=vr0dc8meaabaqaciaacaGaaeqabaqabeGadaaakeaaieaacqWFRbWAdaqhaaWcbaGae8NAaOgabaGae83Camhaaaaa@3101@ executions of all reactions, Rjs
 MathType@MTEF@5@5@+=feaafiart1ev1aaatCvAUfKttLearuWrP9MDH5MBPbIqV92AaeXatLxBI9gBaebbnrfifHhDYfgasaacH8akY=wiFfYdH8Gipec8Eeeu0xXdbba9frFj0=OqFfea0dXdd9vqai=hGuQ8kuc9pgc9s8qqaq=dirpe0xb9q8qiLsFr0=vr0=vr0dc8meaabaqaciaacaGaaeqabaqabeGadaaakeaaieaacqWFsbGudaqhaaWcbaGae8NAaOgabaGae83Camhaaaaa@30CF@ ∈ ***R***^***s ***^or pick a state from a database that can be fired. Else go to 6e;

e. Update the state of all species, xi←xi+∑i=1nkjsνijs fori∈S
 MathType@MTEF@5@5@+=feaafiart1ev1aaatCvAUfKttLearuWrP9MDH5MBPbIqV92AaeXatLxBI9gBaebbnrfifHhDYfgasaacH8akY=wiFfYdH8Gipec8Eeeu0xXdbba9frFj0=OqFfea0dXdd9vqai=hGuQ8kuc9pgc9s8qqaq=dirpe0xb9q8qiLsFr0=vr0=vr0dc8meaabaqaciaacaGaaeqabaqabeGadaaakeaaieaacqWF4baEdaWgaaWcbaGae8xAaKgabeaakiabgcziSkab=Hha4naaBaaaleaacqWFPbqAaeqaaOGaey4kaSYaaabCaeaacqWFRbWAdaqhaaWcbaGae8NAaOgabaGae83CamhaaGGacOGae4xVd42aa0baaSqaaiab=LgaPjab=PgaQbqaaiab=nhaZbaakiabbccaGaWcbaGae8xAaKMaeyypa0JaeGymaedabaGae8NBa4ganiabggHiLdGccqWFMbGzcqWFVbWBcqWFYbGCcqWFGaaicqWFPbqAcqGHiiIZieWacqqFtbWuaaa@509D@.

f. Update the time t = t + τ^s^.

7. If the desired time is reached, stop. Else, go to step 2.

Step 6d is performed to avoid negative populations that may arise from using the stationary PDF to select the representative state of the fast network (see discussion in sub-section c above and Appendix A). Next we demonstrate the strength of the HyMSMC algorithm with the help of simple prototype examples. Real biochemical networks are also considered to demonstrate the generality of the approach to complex networks.

### Algorithm Testing

In each of the following examples, we demonstrate the efficiency and accuracy of the proposed HyMSMC algorithm. Simulations were performed on 2.40 GHz Intel^® ^Xeon(TM) processors. The relaxation of the fast network is assessed with a relaxation tolerance, t_α/2,υ _= 1.96 (α = 0.05,υ > 40) and using windows of 25 MC events. The hybrid solver uses a critical population of X_crit _= 10 and a leap condition tolerance of r = 0.05. In all the examples shown below, the method is accurate with this parameter set. Each numerical example uniquely serves to highlight other novelties of the new HyMSMC scheme.

#### a. Coupled Isomerization Reactions

The first reaction network consists of two pairs of reversible isomerization reactions, coupled through a common species B

A⇄k−1k1BB⇄k−2k2C.
 MathType@MTEF@5@5@+=feaafiart1ev1aaatCvAUfKttLearuWrP9MDH5MBPbIqV92AaeXatLxBI9gBaebbnrfifHhDYfgasaacH8akY=wiFfYdH8Gipec8Eeeu0xXdbba9frFj0=OqFfea0dXdd9vqai=hGuQ8kuc9pgc9s8qqaq=dirpe0xb9q8qiLsFr0=vr0=vr0dc8meaabaqaciaacaGaaeqabaqabeGadaaakqaabeqaaGqaaiab=feabnaao0aaleaacqWFRbWAdaWgaaadbaGaeGymaedabeaaaSqaaiab=TgaRnaaBaaameaacqGHsislcqaIXaqmaeqaaaGccaGLsgIaayjKHaGae8NqaieabaGae8Nqai0aa4qdaSqaaiab=TgaRnaaBaaameaacqaIYaGmaeqaaaWcbaGae83AaS2aaSbaaWqaaiabgkHiTiabikdaYaqabaaakiaawkzicaGLqgcacqWFdbWqcqGGUaGlaaaa@432A@

The above network is one of the simplest networks conducive to QE treatment. The simplicity of this network allows us to clearly demonstrate the key features of the HyMSMC algorithm. It also clarifies the applicability and adaptability of the algorithm to different levels of time-scale and population-scale separation. The rate constants are chosen such that the first reaction pair is much faster than the second reaction pair, and thus it constitutes the fast network. A four orders of magnitude time scale separation exists between the partitioned networks. Based on our definition, species A and B are the fast species and C is the slow species.

**Figure 3:**
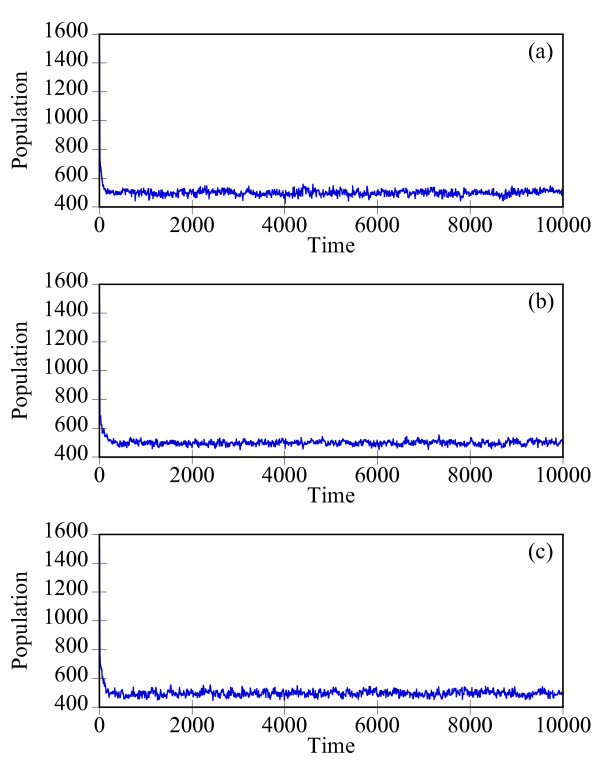
**Temporal trajectories of species A in network (A1)**. The trajectories were generated using (a) the SSA, (b) the MSMC method, and (c) the HyMSMC method. The parameters are k_1 _= k_-1 _= 100 s^-1 ^and k_2 _= k_-2 _= 0.01 s^-1 ^and the initial state is X_A_(t = 0) = 1500, X_B_(t = 0) = X_c_(t = 0) = 0.

**Figure 4:**
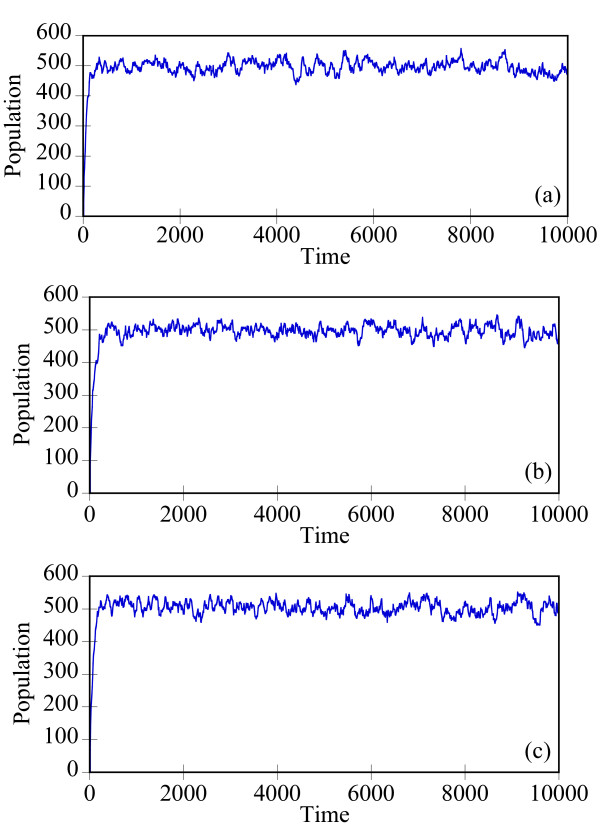
**Temporal trajectories of species C in network (A1)**. The trajectories were generated using (a) the SSA, (b) the MSMC method, and (c) the HyMSMC method. The parameters are those of Figure 3.

Starting with an initial state X_A_(t = 0) = 1500, X_B_(t = 0) = X_c_(t = 0) = 0, we generate stochastic trajectories of network (Al) using the exact SSA, the MSMC method, and the HyMSMC method. As can be seen in Figure 3 and Figure 4, the multiscale methods correctly predict the temporal evolution of the network. The HyMSMC trajectories in Figure 3c and Figure 4c have been generated from states recorded after the occurrence of every slow event, i.e., at every macroscopic step, compared to every millionth MC events in the SSA (Figure 3a and Figure 4a) trajectories. Approximately, 1000 data points have been used to generate all the trajectories seen in Figure 3 and Figure 4. SSA takes around 8 minutes for a single stochastic run from t = 0 to t = 10000 s. The MSMC run is completed in half a minute, and the HyMSMC run, in a fraction of a second. The speedup over the exact SSA is 16 and 320 using the MSMC technique and the HyMSMC technique, respectively. Using number of MC events as a metric, the speedup of the MSMC and HyMSMC methods over the SSA is ~20 and 3600, respectively.

**Figure 5:**
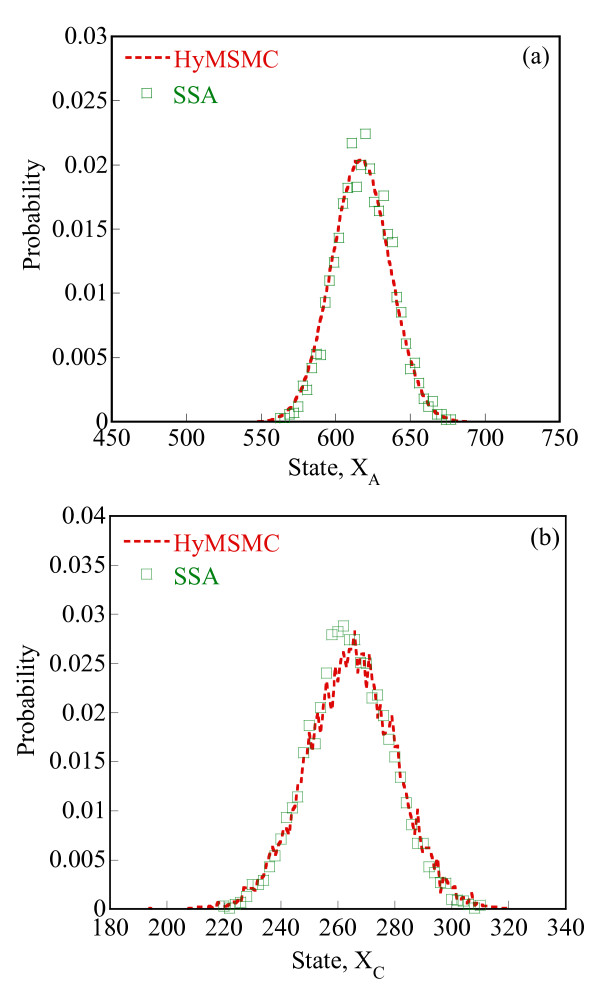
**Probability distribution function (PDF) of (a) species A and (b) species C in network (A1)**. The PDFs were generated at t = 50 s from 10000 trajectories using the SSA (squares) and the HyMSMC method (dotted lines).The parameters are those of Figure 3.

To compare the accuracy of the multiscale methods in a systematic manner, we generate normalized histograms of the states observed at time t = 50 s using 10000 trajectories. Figure 5 shows that the HyMSMC method accurately captures the statistics of the process.

**Figure 6:**
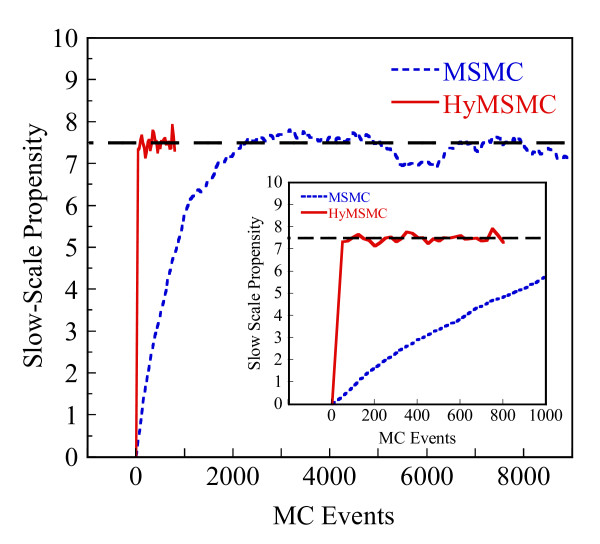
**Comparison of relaxation times of the fast network in network (A1)**. Evolution of the slow-scale propensity, a3¯
 MathType@MTEF@5@5@+=feaafiart1ev1aaatCvAUfKttLearuWrP9MDH5MBPbIqV92AaeXatLxBI9gBaebbnrfifHhDYfgasaacH8akY=wiFfYdH8Gipec8Eeeu0xXdbba9frFj0=OqFfea0dXdd9vqai=hGuQ8kuc9pgc9s8qqaq=dirpe0xb9q8qiLsFr0=vr0=vr0dc8meaabaqaciaacaGaaeqabaqabeGadaaakeaadaqdaaqaaGqaaiab=fgaHnaaBaaaleaacqaIZaWmaeqaaaaaaaa@2F2D@, of reaction 3 in network (Al) before the occurrence of the first slow event B → C, using the MSMC (dotted line) and the HyMSMC (solid line) methods. The horizontal dashed line shows the slow-scale propensity estimated via an analytical description (binomial PDF) of the QE. A magnified view of the initial period is shown in the inset to highlight the rapid convergence of the slow-scale propensity using the hybrid solver of the HyMSMC method. The parameters are those of Figure 3.

The above example clearly demonstrates the superiority of the HyMSMC method over the MSMC method. The strength of the hybrid scheme stems from its ability to handle large populations that typically render SSA solvers inefficient. The hybrid solver quickly relaxes the fast network, even when the pre-relaxation state is far away from the QE. The computational advantage of the hybrid solver, over the SSA, in relaxing the fast network, is demonstrated by comparing the convergence of the slow-scale propensities in Figure 6. The QE PDF of the fast species B is given by the binomial PDF, ℬ
 MathType@MTEF@5@5@+=feaafiart1ev1aaatCvAUfKttLearuWrP9MDH5MBPbIqV92AaeXatLxBI9gBamrtHrhAL1wy0L2yHvtyaeHbnfgDOvwBHrxAJfwnaebbnrfifHhDYfgasaacH8akY=wiFfYdH8Gipec8Eeeu0xXdbba9frFj0=OqFfea0dXdd9vqai=hGuQ8kuc9pgc9s8qqaq=dirpe0xb9q8qiLsFr0=vr0=vr0dc8meaabaqaciaacaGaaeqabaWaaeGaeaaakeaaimaacqWFSeIqaaa@377E@(p,N), where p = k_1_/(k_1 _+ k_-1_) and N = (X_A _+ X_B_). The slow-scale propensity of the linear reaction, B → C, is analytically given by k2Np (see dashed line in Figure 6).

Using this network as an example, we also studied the efficiency of the MSMC and the HyMSMC methods as a function of population scales, at a constant time scale separation. Studying the effect of population under overall equilibrium conditions ensures that the net speedup corresponds to a specific average population. The population scales were varied from O(1) to O(10^6^), and in each case we ran the simulation until a certain time and monitored the CPU time and the number of MC events required to complete the run. The parameters in 5 cases are presented in Table 1. The time scale separation is maintained at around 10^4 ^in all cases.

**Table 1:** Rate constants and initial populations in system (Al) used to maintain the time scale separation at ~O(10^4^) as the population X_0 _increases.

Initial Population X_A _= X_B _= X_C _= X_0_/3	Fast Network k_1 _= k_-1 _(s^-1^)	Slow Network k_2 _= k_-2_(s^-1^)
5	10^4^	1.0
50	10^3^	10^-1^
500	10^2^	10^-2^
5000	10	10^-3^
50000	1.0	10^-4^

The rate constants have been proportionally scaled down with increasing population to maintain the reaction propensities approximately constant.

**Figure 7:**
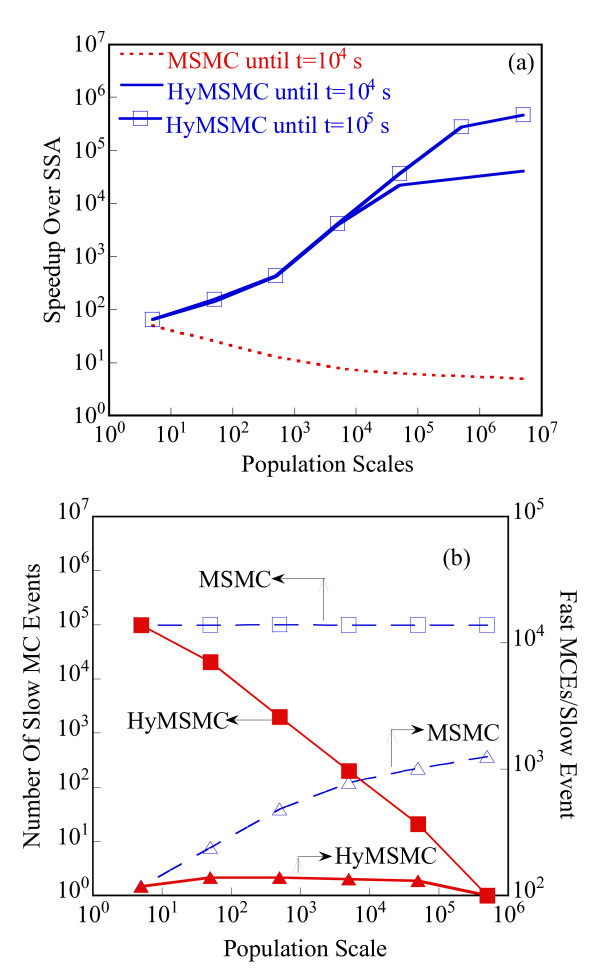
**Effect of population scales on the efficiency of the MSMC and the HyMSMC methods**. (a) Computational acceleration over SSA achieved with the MSMC (dotted line) and the HyMSMC (solid lines for two values of simulation time, 10^4 ^s and 10^5 ^s) methods, as a function of population scales in network (A1). The speedup factor is evaluated as a ratio of CPU time required for an equilibrium simulation of time t, using the SSA and the multiscale schemes. The system parameters used are given in Table 1. (b) Number of slow events (squares) and average number of fast relaxation events per slow event (triangles) to complete a single run of duration t as a function of population. The simulations were performed with the MSMC method (open symbols) and the HyMSMC method (filled symbols) using the parameters given in Table 1.

The cost of relaxing the faster network, gauged by the average number of relaxation events per slow event (Figure 7b), increases with increasing population. As a result, the speedup of the MSMC method over the SSA decreases with increasing population (see Figure 7a).

In contrast to the MSMC method, the speedup achieved with the HyMSMC method improves with increasing population. At low populations, the HyMSMC speedup approaches that of the MSMC technique, because the hybrid solver reduces to the SSA at these population levels that are below the critical population limit. At the other extreme, as the population increases, the number of slow events required to reach a certain time approaches 1 (see Figure 7). As a result, the CPU time and the number of MC events required to reach this end time reduce to the requirements for one slow event. Thus, in the limit of high population, the speedup of the HyMSMC over the exact SSA tends to plateau off. By increasing the end time used for the speedup measurement, say from 10^4 ^s to 10^5 ^s, the leveling of the HyMSMC speedup curve occurs at a higher population (see Figure 7a). In theory, the speedup of the HyMSMC method has a power law dependence on the population scale.

**Figure 8:**
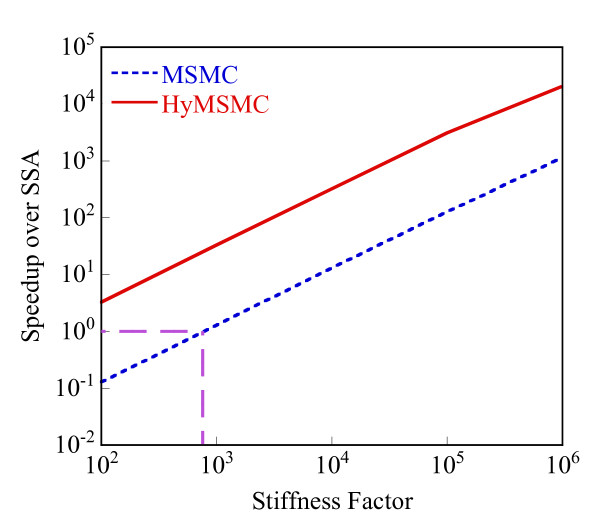
**Effect of time-scale separation on the efficiency of the MSMC and the HyMSMC methods**. Computational acceleration over SSA achieved with the MSMC method (dotted line) and the HyMSMC method (solid line) as a function of time-scale separation in network (Al). The speedup factor is evaluated as a ratio of CPU time required for an equilibrium simulation until t = 10^4 ^s using the SSA and the multiscale methods. The parameters used are presented in Table 2.

Using the same network, we also studied the effect of network stiffness on the speedup achieved at a constant population. The parameters used for this study are presented in Table 2. The network stiffness is approximated by the ratio of the rate constants, O(k_1_/k_2_), given that the populations of all species are in the same range (~500). Figure 8 shows that the acceleration achieved with both multiscale schemes has a power law dependence on stiffness. The speedup of the HyMSMC method lies above that of the MSMC method, highlighting the additional speedup achieved through temporal coarse- graining. As the network stiffness reduces to below 10^3^, the speedup of the MSMC drops below unity, implying that the MSMC method is more expensive than the SSA, and hence should not be used. This is caused by the cost of the MSMC method associated with relaxing the fast network. However, even with this small time-scale separation in the system, the HyMSMC still gives a speedup of around 30 over the SSA due to temporal coarse-graining.

**Table 2:** Rate constants versus network stiffness in system (A1).

Network Stiffness ~O(k_1_/k_2_)	Slow Network k_2 _= k_-2_(s^-1^)
10^2^	1.0
10^3^	10^-1^
10^4^	10^-2^
10^5^	10^-3^
10^6^	10^-4^

k_1 _= k_-1 _= 100 s^-1 ^and X_A_(t = 0) = X_B_(t = 0) = X_C_(t = 0) = 500 are used in all 5 cases.

Finally, we demonstrate the use of CSP to aid network partitioning. The deterministic dynamics of the coupled isomerization network (Al) can be written as

g1=dXAdt=−k1XA+k−1XBg2=dXBdt=k1XA−(k−1+k2)XB+k−2XCg3=dXCdt=k2XB−k−2XC.
 MathType@MTEF@5@5@+=feaafiart1ev1aaatCvAUfKttLearuWrP9MDH5MBPbIqV92AaeXatLxBI9gBaebbnrfifHhDYfgasaacH8akY=wiFfYdH8Gipec8Eeeu0xXdbba9frFj0=OqFfea0dXdd9vqai=hGuQ8kuc9pgc9s8qqaq=dirpe0xb9q8qiLsFr0=vr0=vr0dc8meaabaqaciaacaGaaeqabaqabeGadaaakqaabeqaaGqaaiab=DgaNnaaBaaaleaacqaIXaqmaeqaaOGaeyypa0ZaaSaaaeaacqWFKbazcqWFybawdaWgaaWcbaGae8xqaeeabeaaaOqaaiab=rgaKjab=rha0baacqGH9aqpcqGHsislcqWFRbWAdaWgaaWcbaGaeGymaedabeaakiab=HfaynaaBaaaleaacqWFbbqqaeqaaOGaey4kaSIae83AaS2aaSbaaSqaaiabgkHiTiabigdaXaqabaGccqWFybawdaWgaaWcbaGae8NqaieabeaaaOqaaiab=DgaNnaaBaaaleaacqaIYaGmaeqaaOGaeyypa0ZaaSaaaeaacqWFKbazcqWFybawdaWgaaWcbaGae8NqaieabeaaaOqaaiab=rgaKjab=rha0baacqGH9aqpcqWFRbWAdaWgaaWcbaGaeGymaedabeaakiab=HfaynaaBaaaleaacqWFbbqqaeqaaOGaeyOeI0IaeiikaGIae83AaS2aaSbaaSqaaiabgkHiTiabigdaXaqabaGccqGHRaWkcqWFRbWAdaWgaaWcbaGaeGOmaidabeaakiabcMcaPiab=HfaynaaBaaaleaacqWFcbGqaeqaaOGaey4kaSIae83AaS2aaSbaaSqaaiabgkHiTiabikdaYaqabaGccqWFybawdaWgaaWcbaGae83qameabeaaaOqaaiab=DgaNnaaBaaaleaacqaIZaWmaeqaaOGaeyypa0ZaaSaaaeaacqWFKbazcqWFybawdaWgaaWcbaGae83qameabeaaaOqaaiab=rgaKjab=rha0baacqGH9aqpcqWFRbWAdaWgaaWcbaGaeGOmaidabeaakiab=HfaynaaBaaaleaacqWFcbGqaeqaaOGaeyOeI0Iae83AaS2aaSbaaSqaaiabgkHiTiabikdaYaqabaGccqWFybawdaWgaaWcbaGae83qameabeaakiabc6caUaaaaa@7EE6@

Since the system is linear in **x**, the Jacobian is time-invariant, and thus its eigenvalues and eigenvectors do not evolve with time. The eigenvalues of the Jacobian matrix are Λ_11 _≈ -200, Λ_22 _≈ -0.015, and Λ_33 _= 0. The relaxation time of the fast network approximated from the largest in magnitude eigenvalue, (~|5/Λ_11_|~2.5 × 10^-2^), is consistent with the time estimated from the statistical convergence criterion over the course of a simulation (~ 2.5 × 10^-2^). The corresponding eigenvectors are

b1*=(0.7071−0.70710);b2*=(0.40830.4082−0.8165);b3*=(−0.5774−0.5774−0.5774)
 MathType@MTEF@5@5@+=feaafiart1ev1aaatCvAUfKttLearuWrP9MDH5MBPbIqV92AaeXatLxBI9gBaebbnrfifHhDYfgasaacH8akY=wiFfYdH8Gipec8Eeeu0xXdbba9frFj0=OqFfea0dXdd9vqai=hGuQ8kuc9pgc9s8qqaq=dirpe0xb9q8qiLsFr0=vr0=vr0dc8meaabaqaciaacaGaaeqabaqabeGadaaakeaaieaacqWFIbGydaqhaaWcbaGaeGymaedabaGamaiMcQcaQaaakiabg2da9maabmaabaqbaeqabmqaaaqaaiabicdaWiabc6caUiabiEda3iabicdaWiabiEda3iabigdaXaqaaiabgkHiTiabicdaWiabc6caUiabiEda3iabicdaWiabiEda3iabigdaXaqaaiabicdaWaaaaiaawIcacaGLPaaacqGG7aWocqWFIbGydaqhaaWcbaGaeGOmaidabaGamaiPcQcaQaaakiabg2da9maabmaabaqbaeqabmqaaaqaaiabicdaWiabc6caUiabisda0iabicdaWiabiIda4iabiodaZaqaaiabicdaWiabc6caUiabisda0iabicdaWiabiIda4iabikdaYaqaaiabgkHiTiabicdaWiabc6caUiabiIda4iabigdaXiabiAda2iabiwda1aaaaiaawIcacaGLPaaacqGG7aWocqWFIbGydaqhaaWcbaGaeG4mamdabaGamaiScQcaQaaakiabg2da9maabmaabaqbaeqabmqaaaqaaiabgkHiTiabicdaWiabc6caUiabiwda1iabiEda3iabiEda3iabisda0aqaaiabgkHiTiabicdaWiabc6caUiabiwda1iabiEda3iabiEda3iabisda0aqaaiabgkHiTiabicdaWiabc6caUiabiwda1iabiEda3iabiEda3iabisda0aaaaiaawIcacaGLPaaaaaa@7700@

and the inverse eigenvectors are

b1=[0.7071−0.70713.5×10−5]b2=[0.40830.4083−0.8165]b3=[−0.5774−0.5774−0.5774]
 MathType@MTEF@5@5@+=feaafiart1ev1aaatCvAUfKttLearuWrP9MDH5MBPbIqV92AaeXatLxBI9gBaebbnrfifHhDYfgasaacH8akY=wiFfYdH8Gipec8Eeeu0xXdbba9frFj0=OqFfea0dXdd9vqai=hGuQ8kuc9pgc9s8qqaq=dirpe0xb9q8qiLsFr0=vr0=vr0dc8meaabaqaciaacaGaaeqabaqabeGadaaakqaabeqaaGqaaiab=jgaInaaBaaaleaacqaIXaqmaeqaaOGaeyypa0ZaamWaaeaafaqabeqadaaabaGaeGimaaJaeiOla4IaeG4naCJaeGimaaJaeG4naCJaeGymaedabaGaeyOeI0IaeGimaaJaeiOla4IaeG4naCJaeGimaaJaeG4naCJaeGymaedabaGaeG4mamJaeiOla4IaeGynauJaey41aqRaeGymaeJaeGimaaZaaWbaaSqabeaacqGHsislcqaI1aqnaaaaaaGccaGLBbGaayzxaaaabaGae8Nyai2aaSbaaSqaaiabikdaYaqabaGccqGH9aqpdaWadaqaauaabeqabmaaaeaacqaIWaamcqGGUaGlcqaI0aancqaIWaamcqaI4aaocqaIZaWmaeaacqaIWaamcqGGUaGlcqaI0aancqaIWaamcqaI4aaocqaIZaWmaeaacqGHsislcqaIWaamcqGGUaGlcqaI4aaocqaIXaqmcqaI2aGncqaI1aqnaaaacaGLBbGaayzxaaaabaGae8Nyai2aaSbaaSqaaiabiodaZaqabaGccqGH9aqpdaWadaqaauaabeqabmaaaeaacqGHsislcqaIWaamcqGGUaGlcqaI1aqncqaI3aWncqaI3aWncqaI0aanaeaacqGHsislcqaIWaamcqGGUaGlcqaI1aqncqaI3aWncqaI3aWncqaI0aanaeaacqGHsislcqaIWaamcqGGUaGlcqaI1aqncqaI3aWncqaI3aWncqaI0aanaaaacaGLBbGaayzxaaaaaaa@7821@

**Figure 9:**
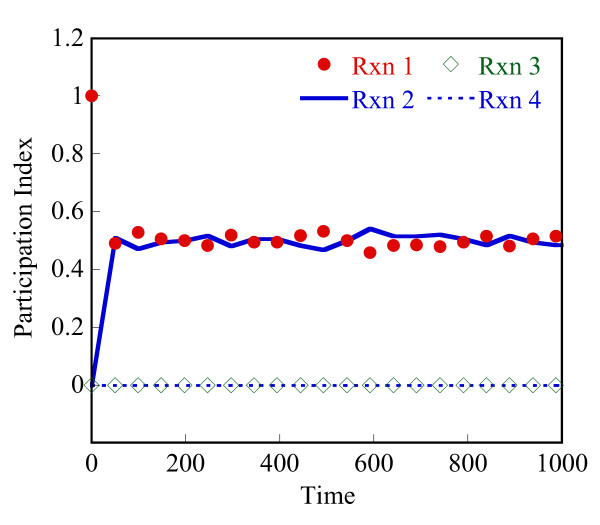
**Participation indices of reactions in the fast mode of network (A1) as a function of time**. The parameters are those of Figure 3.

Based on the magnitude of the eigenvalues, mode 1 is the fast mode. The participation indices of all the reactions in the fast mode are evaluated using (9), and are shown in Figure 9. At small times, when the QE approximation is not yet valid, only reaction 1 is identified as a fast reaction. Then, as the population of X_B _builds up, the contribution of reaction 2 to the fast mode increases, and eventually reactions 1 and 2 contribute almost equally to the fast mode. Thus, the partitioning of the networks with the CSP analysis agrees with that based on the propensities.

#### b. A System with Bistability in the Fast Network

Bistability is a common feature of biological networks, and thus, it is important that the approximate stochastic algorithms capture the correct dynamics of a bistable system. One of the most studied examples of bistabilty is the Schlögl model

X1+2X⇄k−1k13XX2⇄k−2k2X.
 MathType@MTEF@5@5@+=feaafiart1ev1aaatCvAUfKttLearuWrP9MDH5MBPbIqV92AaeXatLxBI9gBaebbnrfifHhDYfgasaacH8akY=wiFfYdH8Gipec8Eeeu0xXdbba9frFj0=OqFfea0dXdd9vqai=hGuQ8kuc9pgc9s8qqaq=dirpe0xb9q8qiLsFr0=vr0=vr0dc8meaabaqaciaacaGaaeqabaqabeGadaaakeaafaqaceGabaaabaacbaGae8hwaG1aaSbaaSqaaiabigdaXaqabaGccqGHRaWkcqaIYaGmcqWFybawdaGdnaWcbaGae83AaS2aaSbaaWqaaiabigdaXaqabaaaleaacqWFRbWAdaWgaaadbaGaeyOeI0IaeGymaedabeaaaOGaayPKHiaawcziaiabiodaZiab=Hfaybqaaiab=HfaynaaBaaaleaacqaIYaGmaeqaaOWaa4qdaSqaaiab=TgaRnaaBaaameaacqaIYaGmaeqaaaWcbaGae83AaS2aaSbaaWqaaiabgkHiTiabikdaYaqabaaakiaawkzicaGLqgcacqWFybawcqGGUaGlaaaaaa@4A2C@

The simplicity of this network makes it an ideal prototype to study the validity of the algorithm in the presence of bimodality. In the above network, we assume that species X_1 _and X_2 _are in excess and act as buffering species. Consequently, the population of species X_1 _and X_2 _can be combined with the rate constants k_1 _and k_2_, to give lumped rate constants k′1
 MathType@MTEF@5@5@+=feaafiart1ev1aaatCvAUfKttLearuWrP9MDH5MBPbIqV92AaeXatLxBI9gBaebbnrfifHhDYfgasaacH8akY=wiFfYdH8Gipec8Eeeu0xXdbba9frFj0=OqFfea0dXdd9vqai=hGuQ8kuc9pgc9s8qqaq=dirpe0xb9q8qiLsFr0=vr0=vr0dc8meaabaqaciaacaGaaeqabaqabeGadaaakeaaieaacuWFRbWAgaqbamaaBaaaleaacqaIXaqmaeqaaaaa@2F38@ and k′2
 MathType@MTEF@5@5@+=feaafiart1ev1aaatCvAUfKttLearuWrP9MDH5MBPbIqV92AaeXatLxBI9gBaebbnrfifHhDYfgasaacH8akY=wiFfYdH8Gipec8Eeeu0xXdbba9frFj0=OqFfea0dXdd9vqai=hGuQ8kuc9pgc9s8qqaq=dirpe0xb9q8qiLsFr0=vr0=vr0dc8meaabaqaciaacaGaaeqabaqabeGadaaakeaaieaacuWFRbWAgaqbamaaBaaaleaacqaIYaGmaeqaaaaa@2F3A@, respectively. The network can be written as

2X⇄k−1k′13X;k′1=X1k1=144(molc−s)−1;k1=6(molc2−s)−1⇄k−2k′2X;k′2=X2k2=5000 molc/s;k2=1276 s−1.
 MathType@MTEF@5@5@+=feaafiart1ev1aaatCvAUfKttLearuWrP9MDH5MBPbIqV92AaeXatLxBI9gBaebbnrfifHhDYfgasaacH8akY=wiFfYdH8Gipec8Eeeu0xXdbba9frFj0=OqFfea0dXdd9vqai=hGuQ8kuc9pgc9s8qqaq=dirpe0xb9q8qiLsFr0=vr0=vr0dc8meaabaqaciaacaGaaeqabaqabeGadaaakeaafaqabeGadaaabaGaeGOmaidcbaGae8hwaG1aa4qdaSqaaiqb=TgaRzaafaWaaSbaaWqaaiabigdaXaqabaaaleaacqWFRbWAdaWgaaadbaGaeyOeI0IaeGymaedabeaaaOGaayPKHiaawcziaiabiodaZiab=HfaybqaaiabcUda7aqaaiqb=TgaRzaafaWaaSbaaSqaaiabigdaXaqabaGccqGH9aqpcqWFybawdaWgaaWcbaGaeGymaedabeaakiab=TgaRnaaBaaaleaacqaIXaqmaeqaaOGaeyypa0JaeGymaeJaeGinaqJaeGinaqJaeiikaGIae8xBa0Mae83Ba8Mae8hBaWMae83yamMaeyOeI0Iae83CamNaeiykaKYaaWbaaSqabeaacqGHsislcqaIXaqmaaGccqGG7aWocqWFRbWAdaWgaaWcbaGaeGymaedabeaakiabg2da9iabiAda2maabmaabaGae8xBa0Mae83Ba8Mae8hBaWMae83yam2aaWbaaSqabeaacqaIYaGmaaGccqGHsislcqWFZbWCaiaawIcacaGLPaaadaahaaWcbeqaaiabgkHiTiabigdaXaaaaOqaamaao0aaleaacuWFRbWAgaqbamaaBaaameaacqaIYaGmaeqaaaWcbaGae83AaS2aaSbaaWqaaiabgkHiTiabikdaYaqabaaakiaawkzicaGLqgcacqWFybawaeaacqGG7aWoaeaacuWFRbWAgaqbamaaBaaaleaacqaIYaGmaeqaaOGaeyypa0Jae8hwaG1aaSbaaSqaaiabikdaYaqabaGccqWFRbWAdaWgaaWcbaGaeGOmaidabeaakiabg2da9iabiwda1iabicdaWiabicdaWiabicdaWiabbccaGiab=1gaTjab=9gaVjab=XgaSjab=ngaJjabc+caViab=nhaZjabcUda7iab=TgaRnaaBaaaleaacqaIYaGmaeqaaOGaeyypa0JaeGymaeJaeGOmaiJaeG4naCJaeGOnayJaeeiiaaIae83Cam3aaWbaaSqabeaacqGHsislcqaIXaqmaaGccqGGUaGlaaaaaa@92EF@

At equilibrium, the network displays a noise-induced switching between the two stable steady states. A typical differential equation solver will relax the above network to either of its stable equilibrium states, depending on the initial state. The deterministic steady states can be obtained by solving the cubic steady-state equation for X

dXdt=k′1X(X−1)2−k−1X(X−1)(X−2)6+k′2−k−2X=0.
 MathType@MTEF@5@5@+=feaafiart1ev1aaatCvAUfKttLearuWrP9MDH5MBPbIqV92AaeXatLxBI9gBaebbnrfifHhDYfgasaacH8akY=wiFfYdH8Gipec8Eeeu0xXdbba9frFj0=OqFfea0dXdd9vqai=hGuQ8kuc9pgc9s8qqaq=dirpe0xb9q8qiLsFr0=vr0=vr0dc8meaabaqaciaacaGaaeqabaqabeGadaaakeaadaWcaaqaaGqaaiab=rgaKjab=Hfaybqaaiab=rgaKjab=rha0baacqGH9aqpdaWcaaqaaiqb=TgaRzaafaWaaSbaaSqaaiabigdaXaqabaGccqWFybawcqGGOaakcqWFybawcqGHsislcqaIXaqmcqGGPaqkaeaacqaIYaGmaaGaeyOeI0YaaSaaaeaacqWFRbWAdaWgaaWcbaGaeyOeI0IaeGymaedabeaakiab=HfayjabcIcaOiab=HfayjabgkHiTiabigdaXiabcMcaPiabcIcaOiab=HfayjabgkHiTiabikdaYiabcMcaPaqaaiabiAda2aaacqGHRaWkcuWFRbWAgaqbamaaBaaaleaacqaIYaGmaeqaaOGaeyOeI0Iae83AaS2aaSbaaSqaaiabgkHiTiabikdaYaqabaGccqWFybawcqGH9aqpcqaIWaamcqGGUaGlaaa@588E@

The reaction rates in (B3) are based on the discrete nature of the reacting species. Solving (B3) for X gives XEqm1
 MathType@MTEF@5@5@+=feaafiart1ev1aaatCvAUfKttLearuWrP9MDH5MBPbIqV92AaeXatLxBI9gBaebbnrfifHhDYfgasaacH8akY=wiFfYdH8Gipec8Eeeu0xXdbba9frFj0=OqFfea0dXdd9vqai=hGuQ8kuc9pgc9s8qqaq=dirpe0xb9q8qiLsFr0=vr0=vr0dc8meaabaqaciaacaGaaeqabaqabeGadaaakeaaieaacqWFybawdaqhaaWcbaGae8xrauKae8xCaeNae8xBa0gabaGaeGymaedaaaaa@32DC@ = 5, XEqm2
 MathType@MTEF@5@5@+=feaafiart1ev1aaatCvAUfKttLearuWrP9MDH5MBPbIqV92AaeXatLxBI9gBaebbnrfifHhDYfgasaacH8akY=wiFfYdH8Gipec8Eeeu0xXdbba9frFj0=OqFfea0dXdd9vqai=hGuQ8kuc9pgc9s8qqaq=dirpe0xb9q8qiLsFr0=vr0=vr0dc8meaabaqaciaacaGaaeqabaqabeGadaaakeaaieaacqWFybawdaqhaaWcbaGae8xrauKae8xCaeNae8xBa0gabaGaeGOmaidaaaaa@32DE@ = 20 and XEqm3
 MathType@MTEF@5@5@+=feaafiart1ev1aaatCvAUfKttLearuWrP9MDH5MBPbIqV92AaeXatLxBI9gBaebbnrfifHhDYfgasaacH8akY=wiFfYdH8Gipec8Eeeu0xXdbba9frFj0=OqFfea0dXdd9vqai=hGuQ8kuc9pgc9s8qqaq=dirpe0xb9q8qiLsFr0=vr0=vr0dc8meaabaqaciaacaGaaeqabaqabeGadaaakeaaieaacqWFybawdaqhaaWcbaGae8xrauKae8xCaeNae8xBa0gabaGaeG4mamdaaaaa@32E0@ = 50 as the steady state solutions of (B2) with XEqm1
 MathType@MTEF@5@5@+=feaafiart1ev1aaatCvAUfKttLearuWrP9MDH5MBPbIqV92AaeXatLxBI9gBaebbnrfifHhDYfgasaacH8akY=wiFfYdH8Gipec8Eeeu0xXdbba9frFj0=OqFfea0dXdd9vqai=hGuQ8kuc9pgc9s8qqaq=dirpe0xb9q8qiLsFr0=vr0=vr0dc8meaabaqaciaacaGaaeqabaqabeGadaaakeaaieaacqWFybawdaqhaaWcbaGae8xrauKae8xCaeNae8xBa0gabaGaeGymaedaaaaa@32DC@ and XEqm3
 MathType@MTEF@5@5@+=feaafiart1ev1aaatCvAUfKttLearuWrP9MDH5MBPbIqV92AaeXatLxBI9gBaebbnrfifHhDYfgasaacH8akY=wiFfYdH8Gipec8Eeeu0xXdbba9frFj0=OqFfea0dXdd9vqai=hGuQ8kuc9pgc9s8qqaq=dirpe0xb9q8qiLsFr0=vr0=vr0dc8meaabaqaciaacaGaaeqabaqabeGadaaakeaaieaacqWFybawdaqhaaWcbaGae8xrauKae8xCaeNae8xBa0gabaGaeG4mamdaaaaa@32E0@ being stable and XEqm2
 MathType@MTEF@5@5@+=feaafiart1ev1aaatCvAUfKttLearuWrP9MDH5MBPbIqV92AaeXatLxBI9gBaebbnrfifHhDYfgasaacH8akY=wiFfYdH8Gipec8Eeeu0xXdbba9frFj0=OqFfea0dXdd9vqai=hGuQ8kuc9pgc9s8qqaq=dirpe0xb9q8qiLsFr0=vr0=vr0dc8meaabaqaciaacaGaaeqabaqabeGadaaakeaaieaacqWFybawdaqhaaWcbaGae8xrauKae8xCaeNae8xBa0gabaGaeGOmaidaaaaa@32DE@ being unstable. The equilibrium solution of the above steady state rate equation, using a generic algebraic solver, such as the Newton's method, depends on the initial guess.

The Schlögl model does not display any separation of time scales. So to apply the hybrid multiscale scheme to this example, the Schlögl model was coupled with a pair of slow reversible isomerization reactions

2X⇄k−1k′13X;k′1=X1k1=144(molc−s)−1;k1=6(molc2−s)−1⇄k−2k′2X;k′2=X2k2=5000 molc/s;k2=1276 s−1X⇄k−3k3Y;k3=0.0005 s−1;k−3=0.0001 s−1.
 MathType@MTEF@5@5@+=feaafiart1ev1aaatCvAUfKttLearuWrP9MDH5MBPbIqV92AaeXatLxBI9gBaebbnrfifHhDYfgasaacH8akY=wiFfYdH8Gipec8Eeeu0xXdbba9frFj0=OqFfea0dXdd9vqai=hGuQ8kuc9pgc9s8qqaq=dirpe0xb9q8qiLsFr0=vr0=vr0dc8meaabaqaciaacaGaaeqabaqabeGadaaakeaafaqabeWadaaabaGaeGOmaidcbaGae8hwaG1aa4qdaSqaaiqb=TgaRzaafaWaaSbaaWqaaiabigdaXaqabaaaleaacqWFRbWAdaWgaaadbaGaeyOeI0IaeGymaedabeaaaOGaayPKHiaawcziaiabiodaZiab=HfaybqaaiabcUda7aqaaiqb=TgaRzaafaWaaSbaaSqaaiabigdaXaqabaGccqGH9aqpcqWFybawdaWgaaWcbaGaeGymaedabeaakiab=TgaRnaaBaaaleaacqaIXaqmaeqaaOGaeyypa0JaeGymaeJaeGinaqJaeGinaqJaeiikaGIae8xBa0Mae83Ba8Mae8hBaWMae83yamMaeyOeI0Iae83CamNaeiykaKYaaWbaaSqabeaacqGHsislcqaIXaqmaaGccqGG7aWocqWFRbWAdaWgaaWcbaGaeGymaedabeaakiabg2da9iabiAda2maabmaabaGae8xBa0Mae83Ba8Mae8hBaWMae83yam2aaWbaaSqabeaacqaIYaGmaaGccqGHsislcqWFZbWCaiaawIcacaGLPaaadaahaaWcbeqaaiabgkHiTiabigdaXaaaaOqaamaao0aaleaacuWFRbWAgaqbamaaBaaameaacqaIYaGmaeqaaaWcbaGae83AaS2aaSbaaWqaaiabgkHiTiabikdaYaqabaaakiaawkzicaGLqgcacqWFybawaeaacqGG7aWoaeaacuWFRbWAgaqbamaaBaaaleaacqaIYaGmaeqaaOGaeyypa0Jae8hwaG1aaSbaaSqaaiabikdaYaqabaGccqWFRbWAdaWgaaWcbaGaeGOmaidabeaakiabg2da9iabiwda1iabicdaWiabicdaWiabicdaWiabbccaGiab=1gaTjab=9gaVjab=XgaSjab=ngaJjabc+caViab=nhaZjabcUda7iab=TgaRnaaBaaaleaacqaIYaGmaeqaaOGaeyypa0JaeGymaeJaeGOmaiJaeG4naCJaeGOnayJaeeiiaaIae83Cam3aaWbaaSqabeaacqGHsislcqaIXaqmaaaakeaacqWFybawdaGdnaWcbaGae83AaS2aaSbaaWqaaiabiodaZaqabaaaleaacqWFRbWAdaWgaaadbaGaeyOeI0IaeG4mamdabeaaaOGaayPKHiaawcziaiab=LfazbqaaiabcUda7aqaaiab=TgaRnaaBaaaleaacqaIZaWmaeqaaOGaeyypa0JaeGimaaJaeiOla4IaeGimaaJaeGimaaJaeGimaaJaeGynauJaeeiiaaIae83Cam3aaWbaaSqabeaacqGHsislcqaIXaqmaaGccqGG7aWocqWFRbWAdaWgaaWcbaGaeyOeI0IaeG4mamdabeaakiabg2da9iabicdaWiabc6caUiabicdaWiabicdaWiabicdaWiabigdaXiabbccaGiab=nhaZnaaCaaaleqabaGaeyOeI0IaeGymaedaaaaakiabc6caUaaa@BBC3@

**Figure 10:**
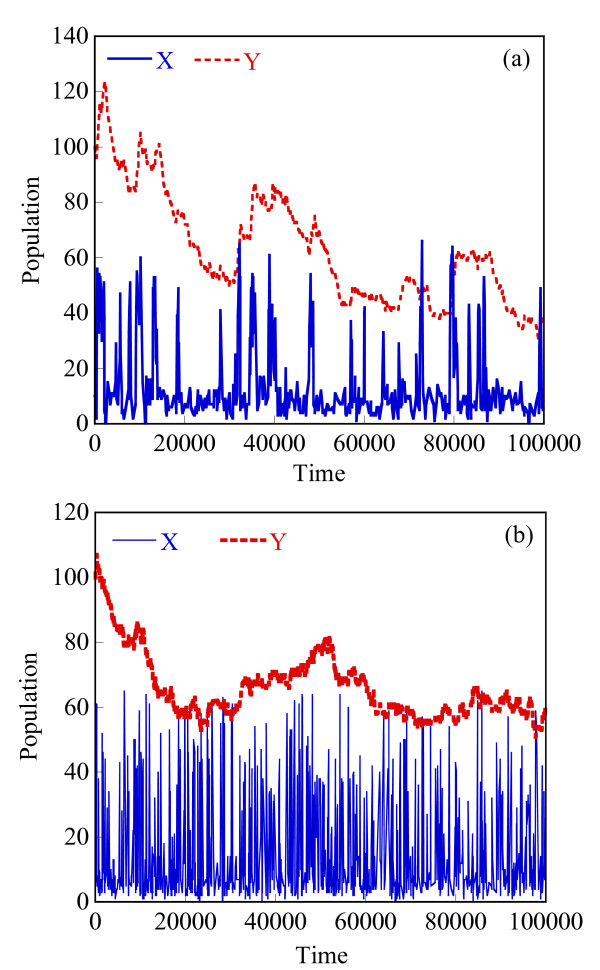
**Temporal trajectories of species X (solid line) and species Y (dashed line) in network (B4)**. The simulation was performed using the HyMSMC method (a) and the SSA (b), with X(t = 0) = 10 and Y(t = 0) = 100 as the starting state.

Thus, in our example, the Schlögl model constitutes the fast network, and the solution of Eq. (B3) is the deterministic QE solution of the fast network. The presence of bimodality in the fast network introduces interesting features in the transfer of information between the fast and the slow networks. Figure 10a and Figure 10b show the population trajectories for species X and Y, using the HyMSMC method and the SSA, respectively. Visually, the HyMSMC technique seems to capture the temporal behavior of the network, including the bistability of the species X. The SSA trajectory appears noisier than the HyMSMC one because it records the numerous fast events that are neglected in the HyMSMC plot. Only the states observed at every coarse time step have been recorded in the HyMSMC plot, compared to every 5 millionth point in the SSA trajectory. Based on the CPU time required for a single run, the HyMSMC and MSMC speedups over the SSA are 9000 and 2800, respectively. Based on MC events, the speedups are ~5000 and 600, respectively. As a result of the moderate size of populations in the network, the HyMSMC method has a rather marginal (~3 in CPU time and ~5 in MC events) advantage over the MSMC method.

**Figure 11:**
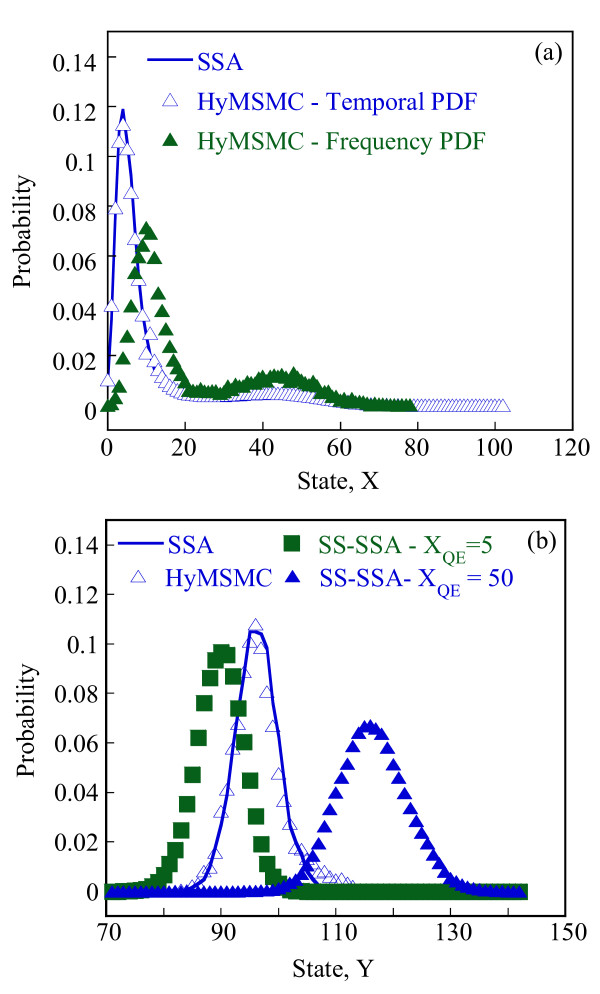
**Probability distribution function (PDF) of species X and species Y in network (B4)**. (a) PDF of species X using the SSA (solid line) and the HyMSMC method (triangles), generated at 1000 s using 10000 trajectories. The PDFs of the HyMSMC method were generated at 1000 s, using temporal averaging for 0.1 s followed by ensemble average (open triangles) and without temporal sampling (just ensemble average; filled triangles). (b) Corresponding PDF of species Y in network (B4) generated using the SSA (solid line) and the HyMSMC (open symbols) and the slow-scale SSA (SS-SSA) [13] (filled symbols) methods. The SS-SSA method uses the mean-field approximation of the fast network to evaluate the slow-scale propensities. The PDFs were generated using X_QE _= 5 (filled squares) and X_QE _= 50 (filled triangles) as the quasi-equilibrium solution of fast network (B2)-(B4). See text for a detailed explanation.

To assess the accuracy of the HyMSMC technique, we compared the PDFs at 1000 s from 10000 trajectories, with those of the SSA. As was mentioned earlier, by selecting the last state at convergence as the representative state at the macroscopic time step, we are implicitly selecting the fast state from the frequency PDF (see Appendix A) present at that macroscopic time. For an accurate histogram of the fast species at any time, their states need to be sampled from the temporal PDF (see Appendix A) at any state of the slow network. In most cases, sampling from the frequency PDF does not cause any noticeable errors in the histogram of the fast species. However, these sampling-induced discrepancies surface in circumstances involving low populations and/or multimodality. This network exemplifies such a situation. Even though the statistics of the slow species Y is correctly captured (open triangles in Figure 11b), there is a distinct mismatch with the SSA results for the fast species (see filled triangles in Figure 11a). It is possible to obtain the correct probabilistic information of the fast network at any time, provided the slow network has been evolved correctly. To demonstrate this, we performed additional sampling of the fast network to obtain the temporal PDF. We then compute the average of the temporal PDFs at 1000 s using 10000 trajectories to account for the joint probability of observing a slow state, **x**^s^, and the relaxed PDF, PDF (**x**^s^), of the fast species at that slow state. Using this simple procedure, the PDFs of both the species match with those obtained using the SSA (see Figure 11 a, open triangles). This corroborates the fact that QE information of the fast network is embedded in the evolution of the slow species, and can be extracted by sufficient time-average sampling when needed. Usually, we do not have an *a priori *knowledge of the differences between the temporal and the frequency PDFs. Therefore, we propose that only the temporal PDF should be used.

We further illustrate the role of the correlations of the fast network, in the evolution of the slow network. In the slow-scale SSA (SS-SSA), Cao et al. [13] used the mean-field approximation of the relaxed fast network to evaluate the slow-scale propensity. In the present example, the fast network has 3 steady-state solutions that are independent of the slow network, due to the buffering species in the Schlögl model. As a result, the deterministic equilibrium solutions, X_Eqm_, of the fast network are the same at every slow step. Assuming that the same initial guess is given to the solver at every macroscopic time step, the bistability of the fast species will not be identified by the SS-SSA. This error creeps into the slow species evolution too. This can be seen in Figure 11b (filled symbols), where using X_Eqm _= 5 or X_Eqm _= 50 to evaluate the slow-scale propensities in the SS-SSA results in a wrong PDF of the slow species. This example emphasizes the need for accurate transfer of information (stochastic closure) between scales, and the failure of the mean-field approximation of the slow-scale propensities for bistable systems.

The CSP analysis is based on the analytical Jacobian of the following set of ODEs

g1=dXdt=k′1X(X−1)2−k−1X(X−1)(X−2)6+k′2−k−2X−k3X+k−3Yg2=dYdt=k3X−k−3Y.
 MathType@MTEF@5@5@+=feaafiart1ev1aaatCvAUfKttLearuWrP9MDH5MBPbIqV92AaeXatLxBI9gBaebbnrfifHhDYfgasaacH8akY=wiFfYdH8Gipec8Eeeu0xXdbba9frFj0=OqFfea0dXdd9vqai=hGuQ8kuc9pgc9s8qqaq=dirpe0xb9q8qiLsFr0=vr0=vr0dc8meaabaqaciaacaGaaeqabaqabeGadaaakqaabeqaaGqaaiab=DgaNnaaBaaaleaacqaIXaqmaeqaaOGaeyypa0ZaaSaaaeaacqWFKbazcqWFybawaeaacqWFKbazcqWF0baDaaGaeyypa0ZaaSaaaeaacuWFRbWAgaqbamaaBaaaleaacqaIXaqmaeqaaOGae8hwaGLaeiikaGIae8hwaGLaeyOeI0IaeGymaeJaeiykaKcabaGaeGOmaidaaiabgkHiTmaalaaabaGae83AaS2aaSbaaSqaaiabgkHiTiabigdaXaqabaGccqWFybawcqGGOaakcqWFybawcqGHsislcqaIXaqmcqGGPaqkcqGGOaakcqWFybawcqGHsislcqaIYaGmcqGGPaqkaeaacqaI2aGnaaGaey4kaSIaf83AaSMbauaadaWgaaWcbaGaeGOmaidabeaakiabgkHiTiab=TgaRnaaBaaaleaacqGHsislcqaIYaGmaeqaaOGae8hwaGLaeyOeI0Iae83AaS2aaSbaaSqaaiabiodaZaqabaGccqWFybawiiaacqGFRaWkcqWFRbWAdaWgaaWcbaGaeyOeI0IaeG4mamdabeaakiab=Lfazbqaaiab=DgaNnaaBaaaleaacqaIYaGmaeqaaOGaeyypa0ZaaSaaaeaacqWFKbazcqWFzbqwaeaacqWFKbazcqWF0baDaaGaeyypa0Jae83AaS2aaSbaaSqaaiabiodaZaqabaGccqWFybawcqGHsislcqWFRbWAdaWgaaWcbaGaeyOeI0IaeG4mamdabeaakiab=Lfazjabc6caUaaaaa@7779@

**Figure 12:**
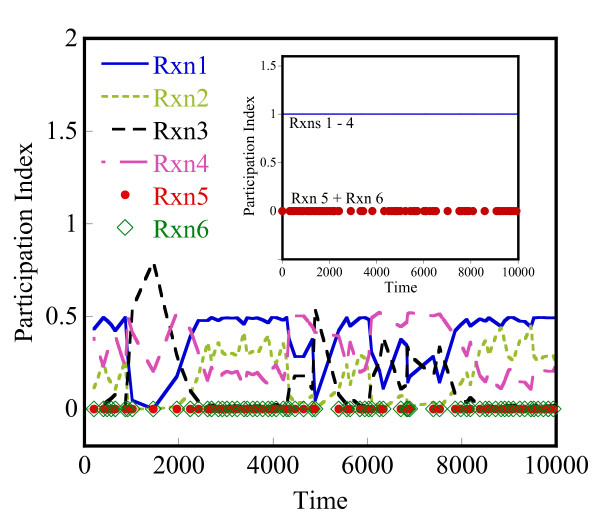
**Participation indices in the fast mode versus time, for reactions 1–6 in network (B4)**. The inset shows the combined participation indices of reactions 1–4, and of reactions 5 and 6 as a function of time.

The two eigenvalues are well separated, indicative of a significant scale separation in the network. Specifically, |Λ_22_| is ~10^-4 ^and |Λ_11_| fluctuates between negative and positive values (this is characteristic of sampling states from the unstable attractor as the system transitions from one branch to the other) but is of the order of 10^2^-10^3^. The temporal profile of the participation indices in the fast mode is presented in Figure 12. The combined participation of reactions 5 and 6 is negligible at all times. The individual contribution of reactions 1 to 4 fluctuates a lot, but their combined participation to the fast mode equals ~1, see inset in Figure 12, implying that the fast mode is comprised of the first four reactions. Thus, the partitioning of the two methods (propensity and CSP based) is consistent.

Assessment of convergence in this bistable system is more complicated. The relaxation time estimated via CSP provides the time needed to sample a branch. For bistable systems, aside from two relaxation times, there is also a time scale related to the (residence) time the system stays in a branch. In order to adequately sample both branches, one needs to simulate the system for times longer than all three time scales. The mean residence time of the fast network was estimated to be ~0.01 s on the higher branch (X ~50) and ~0.04 s on the lower branch (X ~5). The relaxation time of the fast network based on magnitude of the eigenvalues lies in the range O(10^-2^)-O(10^-3^). Thus, in this case, due to the comparable magnitude of residence times and relaxation times, the relaxation time based on the largest in magnitude eigenvalue gives a reasonable estimate of the sampling time of the fast network. In our simulation, we simulated the fast network for an additional 0.1 s, which was decided as a multiple of the sum of the residence times on each of the bifurcation branches.

#### c. Gene Expression Model: Bistability in the Slow Network

Having established the benefits of the algorithms with simple networks, we focus on real biological networks. The positive feedback-regulated gene expression model proposed in the work of Kepler and Elston [1] mathematically proved that fluctuations between operator states are a potential source of stochasticity in the level of translated protein, even at high mean populations. The gene expression model used by Kepler and Elston is an excellent biological example of "multiscales plaguing stochastic simulation". Unlike the previous example where the bistability was present in the fast network, in this gene expression model, the bistability is rooted in the slow network, and propagates to the fast network.

In the gene expression model shown below, the dimerized form, D, of the gene product, M, activates the gene in a positive feedback manner, by binding its operator site.

2M⇄k−1k1Dk1=100(molc−s)−1; k−1=106s−1θ0+D⇄k−2k2θ1k2=0.15(molc−s)−1; k−2=1.5s−1θ0→k3θ0+Mk3=50s−1θ1→k4θ1+Mk4=1000s−1M→k5k5=1s−1.
 MathType@MTEF@5@5@+=feaafiart1ev1aaatCvAUfKttLearuWrP9MDH5MBPbIqV92AaeXatLxBI9gBaebbnrfifHhDYfgasaacH8akY=wiFfYdH8Gipec8Eeeu0xXdbba9frFj0=OqFfea0dXdd9vqai=hGuQ8kuc9pgc9s8qqaq=dirpe0xb9q8qiLsFr0=vr0=vr0dc8meaabaqaciaacaGaaeqabaqabeGadaaakeaafaqadeqbcaaaaeaacqaIYaGmieaacqWFnbqtdaGdnaWcbaGae83AaS2aaSbaaWqaaiabigdaXaqabaaaleaacqWFRbWAdaWgaaadbaGaeyOeI0IaeGymaedabeaaaOGaayPKHiaawcziaiab=reaebqaaiab=TgaRnaaBaaaleaacqaIXaqmaeqaaOGaeyypa0JaeGymaeJaeGimaaJaeGimaaJaeiikaGIae8xBa0Mae83Ba8Mae8hBaWMae83yamMaeyOeI0Iae83CamNaeiykaKYaaWbaaSqabeaacqGHsislcqaIXaqmaaGccqGG7aWocqqGGaaicqWFRbWAdaWgaaWcbaGaeyOeI0IaeGymaedabeaakiabg2da9iabigdaXiabicdaWmaaCaaaleqabaGaeGOnaydaaOGae83Cam3aaWbaaSqabeaacqGHsislcqaIXaqmaaaakeaaiiGacqGF4oqCdaWgaaWcbaGaeGimaadabeaakiabgUcaRiab=reaenaao0aaleaacqWFRbWAdaWgaaadbaGaeGOmaidabeaaaSqaaiab=TgaRnaaBaaameaacqGHsislcqaIYaGmaeqaaaGccaGLsgIaayjKHaGae4hUde3aaSbaaSqaaiabigdaXaqabaaakeaacqWFRbWAdaWgaaWcbaGaeGOmaidabeaakiabg2da9iabicdaWiabc6caUiabigdaXiabiwda1iabcIcaOiab=1gaTjab=9gaVjab=XgaSjab=ngaJjabgkHiTiab=nhaZjabcMcaPmaaCaaaleqabaGaeyOeI0IaeGymaedaaOGaei4oaSJaeeiiaaIae83AaS2aaSbaaSqaaiabgkHiTiabikdaYaqabaGccqGH9aqpcqaIXaqmcqGGUaGlcqaI1aqncqWFZbWCdaahaaWcbeqaaiabgkHiTiabigdaXaaaaOqaaiab+H7aXnaaBaaaleaacqaIWaamaeqaaOWaa4ajaSqaaiab=TgaRnaaBaaameaacqaIZaWmaeqaaaWcbeGccaGLsgcacqGF4oqCdaWgaaWcbaGaeGimaadabeaakiabgUcaRiab=1eanbqaaiab=TgaRnaaBaaaleaacqaIZaWmaeqaaOGaeyypa0JaeGynauJaeGimaaJae83Cam3aaWbaaSqabeaacqGHsislcqaIXaqmaaaakeaacqGF4oqCdaWgaaWcbaGaeGymaedabeaakmaaoqcaleaacqWFRbWAdaWgaaadbaGaeGinaqdabeaaaSqabOGaayPKHaGae4hUde3aaSbaaSqaaiabigdaXaqabaGccqGHRaWkcqWFnbqtaeaacqWFRbWAdaWgaaWcbaGaeGinaqdabeaakiabg2da9iabigdaXiabicdaWiabicdaWiabicdaWiab=nhaZnaaCaaaleqabaGaeyOeI0IaeGymaedaaaGcbaGae8xta00aa4ajaSqaaiab=TgaRnaaBaaameaacqaI1aqnaeqaaaWcbeGccaGLsgcaaeaacqWFRbWAdaWgaaWcbaGaeGynaudabeaakiabg2da9iabigdaXiab=nhaZnaaCaaaleqabaGaeyOeI0IaeGymaedaaOGaeiOla4caaaaa@C05C@

**Figure 13:**
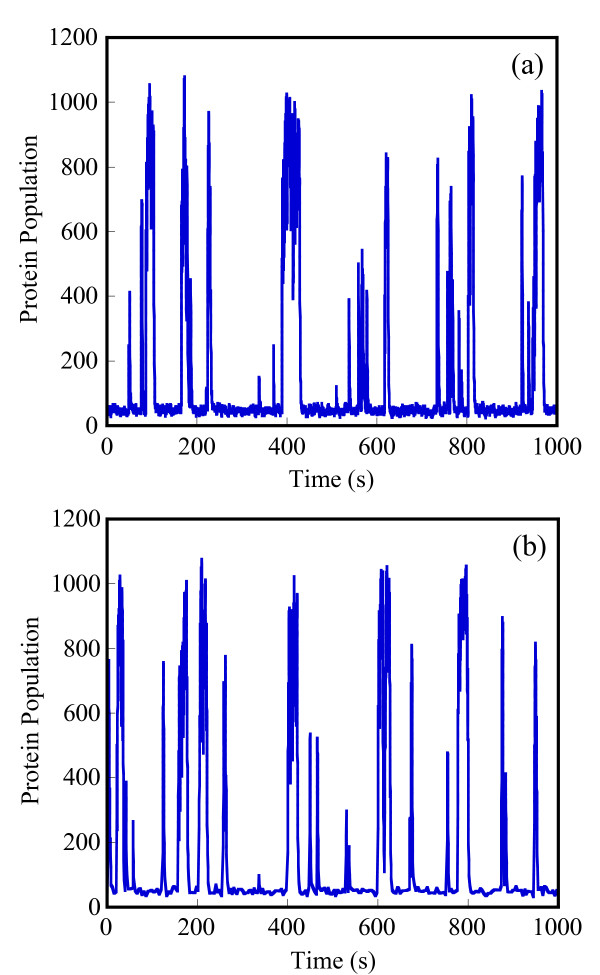
**Population trajectories of the protein species in network (C1)**. The trajectories were generated using (a) the HyMSMC method and (b) the SSA. The simulation was performed using k_1 _= 100 (molc-s)^-1^, k_-1 _= 100 s^-1^, k_2 _= 0.15 (molc-s) ^-1^, k_-2 _= 1.5 s^-1^, k_3 _= 50 s^-1^, k_4 _= 1000 s^-1^, k_5 _= 1 s^-1 ^and an initial state X_M_(t = 0) = 50, X_D_(t = 0) = 0, X_θ0_(t = 0) = 1 and X_θ1_(t = 0) = 0.

**Figure 14:**
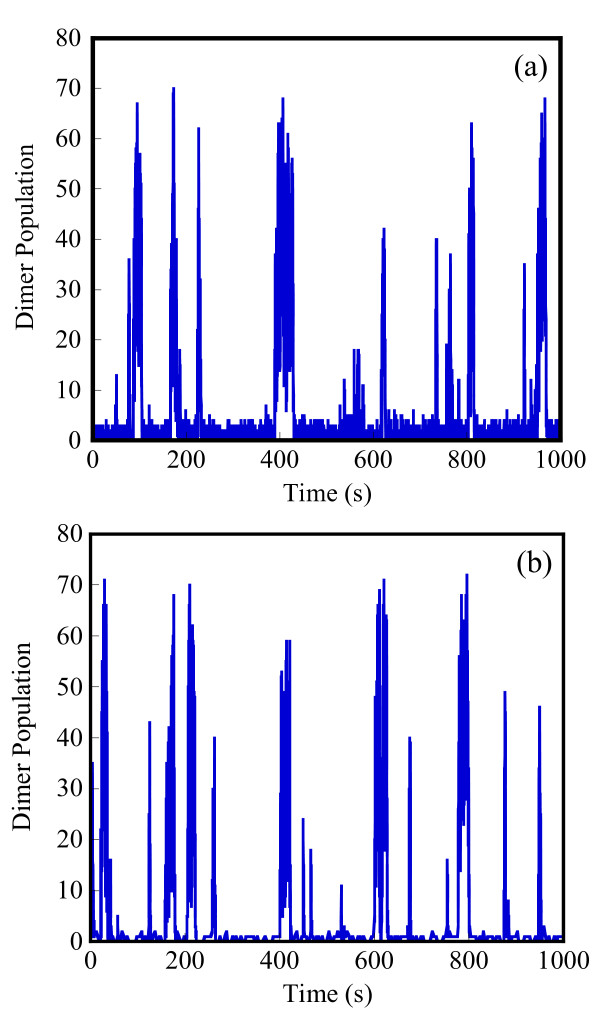
**Population trajectories of the dimer species in network (C1)**. The trajectories were generated using (a) the HyMSMC method and (b) the SSA. The parameters are those of Figure 14.

The separation of scales comes about because the dimerization reaction and its reverse reaction proceed at a much faster rate than the remaining reactions in the network. Hence, in the simulations performed using the exact SSA, most of the simulation time is spent firing the first set of reversible reactions. In simulating 1000 seconds of real time, the HyMSMC algorithm accelerates the simulation by a factor of 140, based on CPU times, and by a factor of 400, based on the number of MC events. The trajectories for the gene product, M, and its dimer, D, are in good visual agreement with those generated using the SSA, as shown in Figure 13 and Figure 14.

**Figure 15:**
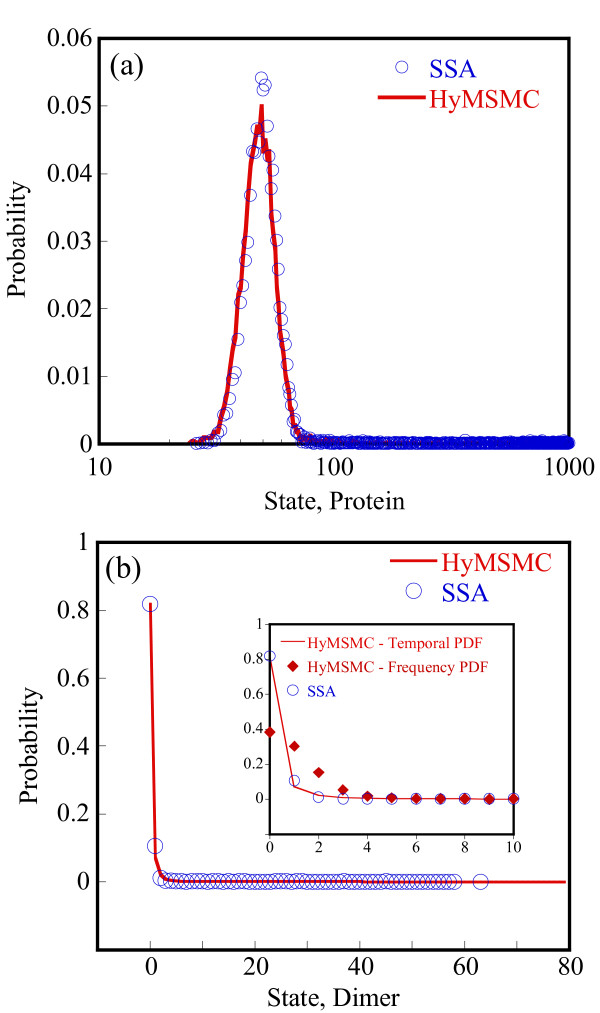
**Probability distribution function (PDF) of the (a) protein and the (b) dimer species in network (C1)**. The PDFs were generated at t = 10 s from 100000 SSA (circles) trajectories and 10000 HyMSMC (solid line) trajectories. The inset in (b) shows a magnified view of the PDF of the dimer species at low population. The parameters are those of Figure 14.

PDFs generated at t = 10 s using the HyMSMC method are in excellent agreement with those of the exact SSA (Figure 15). The low population of the dimer exemplifies a situation where the temporal PDF of the fast network is different from the frequency PDF. As a result, the PDF of the dimer species, generated using the HyMSMC method is in disagreement with the SSA PDF at low population states. However, sampling (t_samp _= 0.001 s) the fast network at 10 s to collect the temporal PDF, followed by the ensemble average eliminates this discrepancy (see inset in Figure 15b).

**Figure 16:**
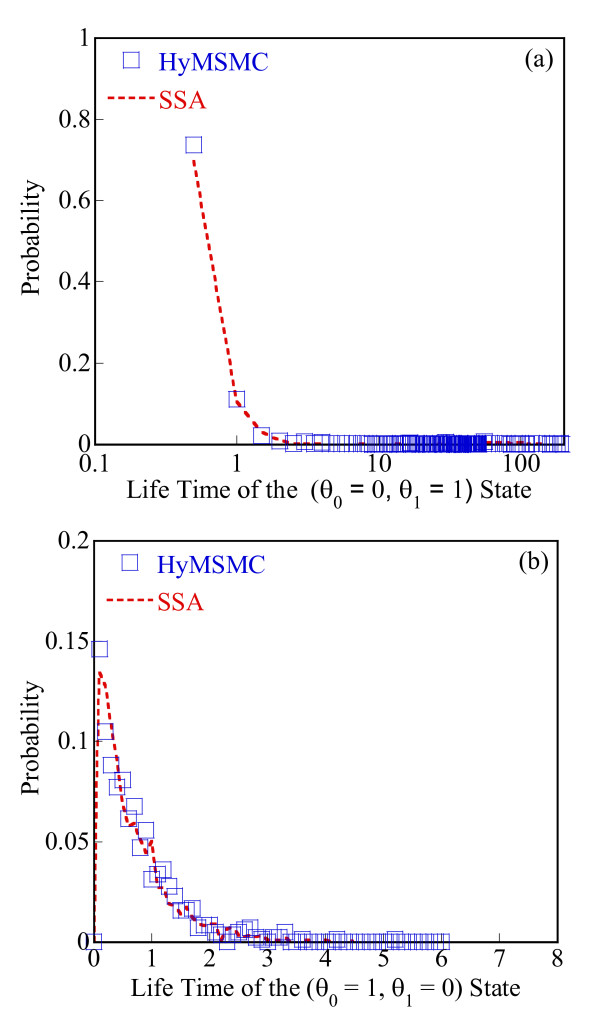
**Probability distribution function (PDF) of the life times of (a) θ_0 _= 1, θ_1 _= 0 state and (b) θ_0 _= 0, θ_1 _= 1 state**. The PDFs were generated using the HyMSMC method (open squares) and the SSA (dashed line). The parameters are those of Figure 14.

As an additional test of accuracy of the HyMSMC method, we compared the distributions of the life-time of the states of the gene (θ_0 _= 0 and θ_0 _= 1) obtained from a single equilibrium run of the HyMSMC method and the SSA. Figure 16 shows that the HyMSMC method correctly captures the PDF of life time of both states.

**Figure 17:**
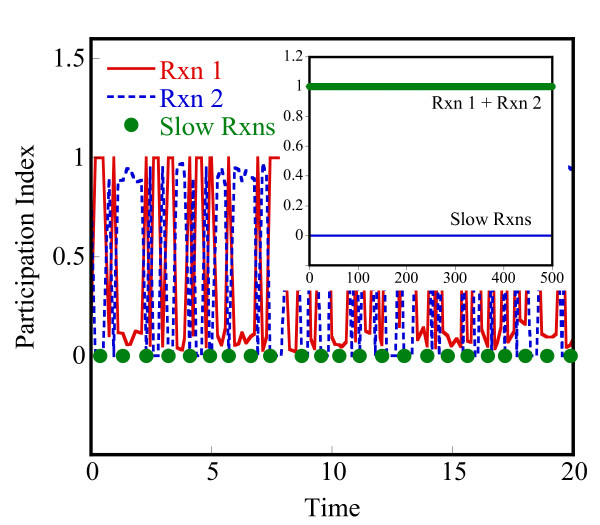
**Participation index (PI) of reactions in the fast modes of network (C1) as a function of time**. The inset shows that the combined PI of reactions 1 and 2 almost equals 1.

Finally, the CSP-analysis shows that there is one predominant eigenvalue (|Λ_11_| ~10^6^) at all times, implying the presence of one fast mode. The microsolver relaxes the fast network in ~10^-4 ^time units based on the statistical criterion, which is greater than the eigenvalue-based relaxation time of 5 × l0^-6^, implying that our statistical relaxation criterion is more conservative and dominates convergence in this example. The contribution of the reactions to the fast mode is shown in Figure 17. The fast mode almost completely consists of reactions 1 and 2 (see inset in Figure 17). However, the contribution of reaction 2 drops to zero when the D = 0 state is encountered. In such a situation, the CSP-identified fast network will consist of only reaction 1. Even the rank based partitioning faces similar problems in such a situation. One way to tackle the problem is to base the partitioning of the network on average propensity values evaluated from a few (depending on network size) MC events. These averaged propensities could effectively be used to perform the CSP analysis.

#### d. Heat Shock Response (HSR) Model

As a final example, we apply the algorithm to the heat shock response model in E. Coli. On sensing high temperature, the cell triggers off a network of biochemical reactions at the genetic and cytoplasmic level to prevent or reduce protein denaturation caused by elevated temperatures. The physiological behavior resulting from these reactions is termed the heat shock response (HSR). Takahashi et al. [29] and E et al. [20] used a modified version of the HSR model as an example of a biological network with multiple time scales. The biochemical reactions constituting the HSR model are presented in Table 3. A large separation of time scales is present in this network, with the first 3 reactions shown in Table 3 being around six orders of magnitude faster than the rest. We applied the HyMSMC scheme to the HSR network.

**Table 3:** Reaction network of the heat shock response model.

**RxnNo.**	**Reactions**	**Rate const**	**Reaction Type**
1	Protein → UnfProt	0.2	Unfolding
2	UnfProt + DnaJ → UnfProt:DnaJ	0.0108	Association
3	UnfProt:DnaJ → Protein + DnaJ	0.2	Dissociation/Folding
4	DNA(σ32) → mRNA(σ32)	1.40E-03	Transcription
5	mRNA(σ32) → σ32	0.07	Translation
6	mRNA(σ32) → degrades	1.40E-06	Degradation
7	σ32 → σ32_RNAP_	0.7	Conformational
8	σ32_RNAP _→ σ32	1.3E-01	Conformational
9	DNA(DnaJ) + σ32_RNAP _→ DNA(DnaJ) + σ32 + DnaJ	4.88E-03	Transcription combined with Translation
10	DNA(FtsH) + σ32_RNAP _→ DNA(FtsH) + σ32 + FtsH	4.88E-03	
11	DNA(GroEL) + σ32_RNAP _→ DNA(GroEL) + σ32 + GroEL	6.29E-03	
12	DnaJ → degrades	6.40E-10	Degradation
13	FtsH → degrades	7.40E-11	
14	GroEL → degrades	1.80E-08	
15	σ32 + DnaJ → σ32:DnaJ	3.62E-04	Association
16	σ32:DnaJ → σ32 + DnaJ	4.40E-04	Dissociation
17	FtsH + σ32:DnaJ → DnaJ + FtsH	1.42E-06	

The units of stochastic rate constants are s^-1 ^for 1^st^-order reactions and (molecule-s)^-1 ^for 2^nd^-order reactions. DNA(X) and mRNA(X) correspond to DNA sequence and mRNA transcripts, respectively, for species X. DNA (or mRNA) molecules do not get consumed in the transcription (or translation) reactions, but simply provide a template for mRNA (or protein) formation.

**Figure 18:**
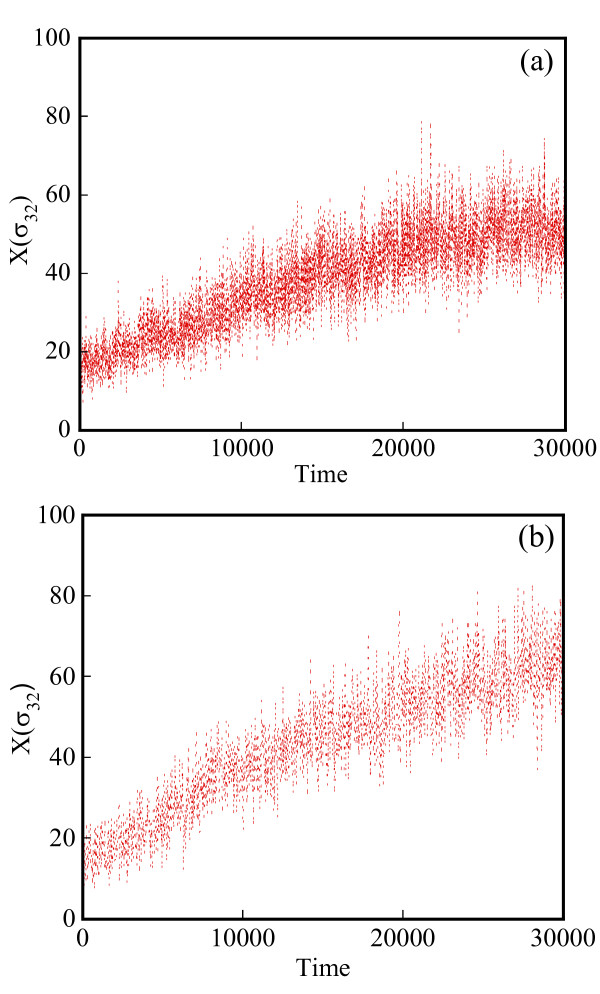
**Temporal profile of species σ^32 ^in the heat shock response model**. Simulations were performed using (a) the HyMSMC method and (b) the SSA. The parameters are given in Table 3.

**Figure 19:**
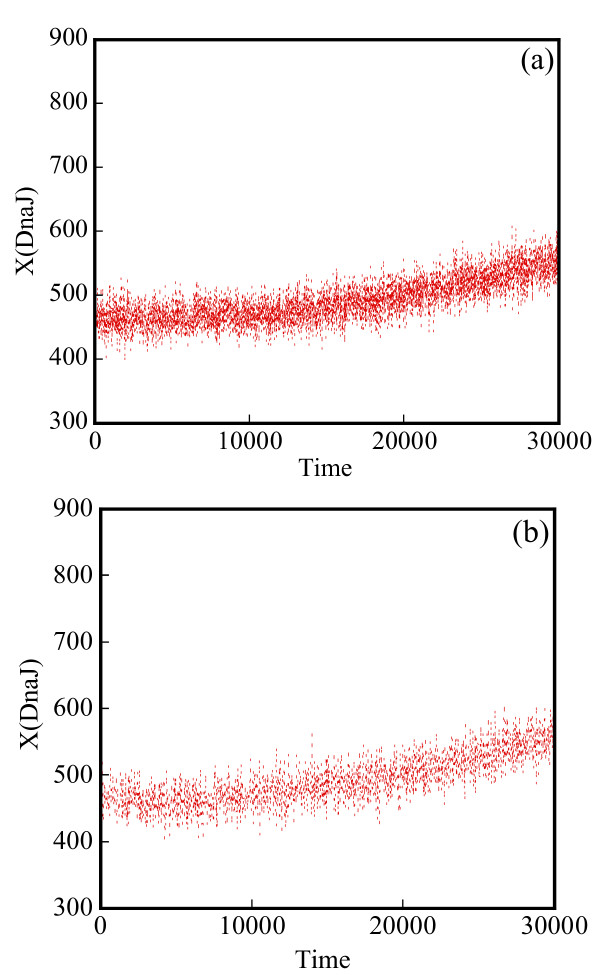
**Temporal profile of species DnaJ in the heat shock response model**. The trajectories were obtained using (a) the HyMSMC method and (b) the SSA. The parameters are given in Table 3.

**Figure 20:**
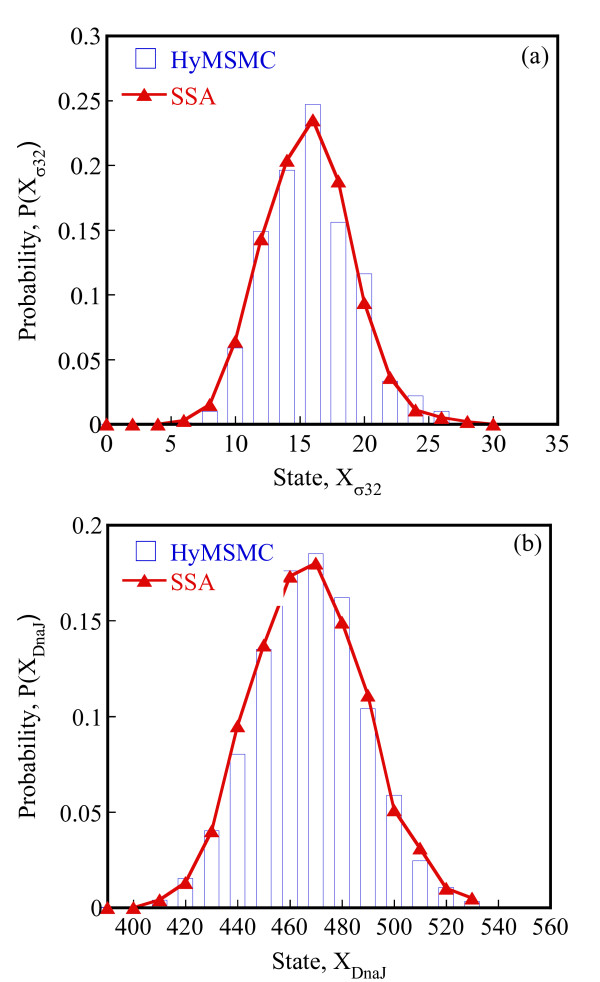
**Normalized histograms of species in the heat shock response model**. The normalized histograms for (a) σ^32 ^and (b) DnaJ at time t = 1 s were obtained by using 1000 trajectories generated with the SSA (symbols) and the HyMSMC (bars).

The transient behavior of the network is captured accurately using the HyMSMC, as can be seen in Figure 18 and Figure 19 which compares trajectories of two species with those of the SSA. Species σ^32 ^and DnaJ were chosen from the fast and slow network, respectively, to demonstrate the accuracy of the algorithm at all scales. Using the HyMSMC method requires 6 s (7 × l0^5 ^MC events) to reach t = 300, compared to 1200 s (9 × 10^8^MC events) required by SSA, thus accelerating the simulation by a factor of 200 (1000 based on MC events). As a test of accuracy, the normalized histograms of states at t = 1 s from 1000 trajectories were generated. The results are in good agreement, as shown in Figure 20. The algorithm accurately captures the noise of species σ^32 ^despite the low population involved (see Figure 20b).

**Figure 21:**
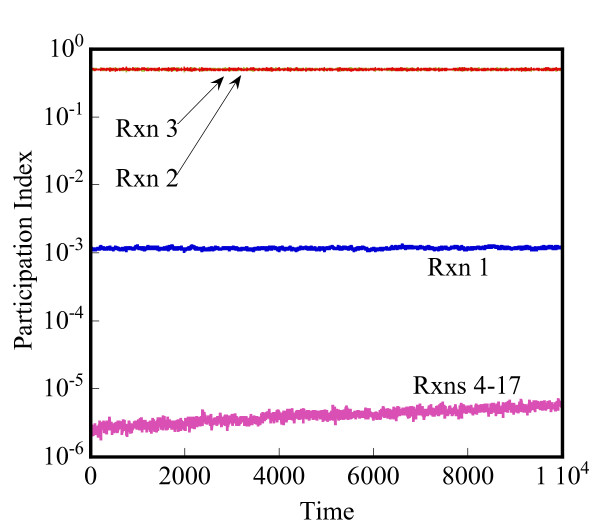
Participation indices of reactions in heat shock response model (Table 3) to the fast mode as a function of time.

The CSP analysis identifies one fast mode in the HSR network, whose eigenvalue is 3 orders of magnitude larger than the next fastest mode. The relaxation time estimated from the eigenvalues (~2.5 × l0^-3^) is in good agreement with the one based on the statistical convergence criterion (order of ~1.2 × l0^-3^). The temporal profile of the participation indices, shown in Figure 21, identifies the first 3 reactions in the network as the fast reactions that contribute the most to the fastest mode. The combined participation index of reactions 4 to 17 is negligible compared to the participation index of reactions 1 to 3. Thus, reactions 4–17 are the slow reactions. Figure 21 also shows that for the time considered, the network partitioning is maintained. Thus, even for a moderately large network, the CSP method offers a systematic method to partition the network. For the HSR model, the CSP analysis increased the CPU cost of the HyMSMC method by less than 4%. Thus, for the largest network simulated in this paper, the CPU overhead of the CSP-assisted partitioning method is negligible.

## Conclusion

In this paper, we have presented a hybrid, multiscale Monte Carlo (HyMSMC) algorithm that can *simultaneously *handle disparity of timescales and species population in well-mixed stochastic networks. The HyMSMC method uses a hybrid SSA-i-leap method [28] as the microscopic and the macroscopic solvers, and employs stochastic singular perturbation concepts when time scale separation exists. As a result, it is able to systematically exploit large populations in the fast and/or slow networks to accelerate stochastic simulations without losing accuracy. Importantly, the absence of *a priori *assumptions about the scales in the network makes the HyMSMC method applicable over a wide range of the population and time-scale separation. Consequently, the HyMSMC method can switch between the exact SSA [3,4], the τ-leap [6], or the MSMC [17] method in a dynamic manner, depending on the instantaneous timescales and populations in the network.

The superiority of the HyMSMC method over the original MSMC method [17] and the SSA [3,4] was demonstrated using various examples, including two networks displaying bistability. The core of most multiscale algorithms is the closures between scales, which strongly influence accuracy. Using the Schlögl model and a simple gene expression model, we showed that the closures used in the HyMSMC method accurately capture the bimodality in the fast and the slow networks.

A second contribution of this paper is the stochastic partitioning and convergence that have not extensively been discussed in previous work. We introduced the computational singular perturbation (CSP) technique [25,26] as a diagnostic tool to systematically identify the fast and the slow networks. In all examples, CSP was able to correctly partition the network and provide estimates of relaxation time for converging the fast network. This latter use of eigenvalues in conjunction with statistical tests is strongly recommended in order to eliminate numerical artifacts of the latter. The computational overhead associated with the evaluation of the Jacobian matrix and the eigenvalue analysis has been small for the examples studied here.

## Authors' contributions

AS developed the algorithm, set up and performed the simulations and drafted the manuscript. DGV and BAO conceived the study, and participated in its design and coordination and helped refine the manuscript. All authors read and approved the final manuscript.

## Appendix A: Evaluation of PDFs and Slow Scale Propensities

### Calculation of PDFs

Once a network has been partitioned, one needs to relax the fast sub-network *(microscopic solver) *until the stochastic low-dimensional manifold has been reached, then compute the QE PDF of the fast network, and subsequently use it to evolve the slow network. In our original MSMC method [17], this was accomplished using the SSA but this can also be done with the τ-leap method. We compute the time-averaged PDF of the fast states

P∞(xf|x)≈ΔtfτEqm,
 MathType@MTEF@5@5@+=feaafiart1ev1aaatCvAUfKttLearuWrP9MDH5MBPbIqV92AaeXatLxBI9gBaebbnrfifHhDYfgasaacH8akY=wiFfYdH8Gipec8Eeeu0xXdbba9frFj0=OqFfea0dXdd9vqai=hGuQ8kuc9pgc9s8qqaq=dirpe0xb9q8qiLsFr0=vr0=vr0dc8meaabaqaciaacaGaaeqabaqabeGadaaakeaaieaacqWFqbaudaWgaaWcbaGaeyOhIukabeaakmaabmaabaacbeGae4hEaG3aaWbaaSqabeaacqGFMbGzaaGccqGG8baFcqGF4baEaiaawIcacaGLPaaacqGHijYUdaWcaaqaaiabgs5aejab=rha0naaCaaaleqabaGae8NzaygaaaGcbaacciGae0hXdq3aaSbaaSqaaiab=veafjab=fhaXjab=1gaTbqabaaaaOGaeiilaWcaaa@43D5@

where Δt^f ^is the total life time of the state **x**^**f **^in an equilibrium simulation of the fast network for time τEqm=∑xf′Δtf′
 MathType@MTEF@5@5@+=feaafiart1ev1aaatCvAUfKttLearuWrP9MDH5MBPbIqV92AaeXatLxBI9gBaebbnrfifHhDYfgasaacH8akY=wiFfYdH8Gipec8Eeeu0xXdbba9frFj0=OqFfea0dXdd9vqai=hGuQ8kuc9pgc9s8qqaq=dirpe0xb9q8qiLsFr0=vr0=vr0dc8meaabaqaciaacaGaaeqabaqabeGadaaakeaaiiGacqWFepaDdaWgaaWcbaacbaGae4xrauKae4xCaeNae4xBa0gabeaakiabg2da9maaqafabaGaeyiLdqKae4hDaq3aaWbaaSqabeaacuGFMbGzgaqbaaaaaeaacqGF4baEdaahaaadbeqaaiqb+zgaMzaafaaaaaWcbeqdcqGHris5aaaa@3D0C@, given that the slow system is at state **x**^**s**^. We obtain Δt^f ^by monitoring the life-time of states accessed once at equilibrium.

In the literature, it is common to generate a PDF based on the frequency of observing a state

P∞(xf|x)≈MCEfMCEEqm.
 MathType@MTEF@5@5@+=feaafiart1ev1aaatCvAUfKttLearuWrP9MDH5MBPbIqV92AaeXatLxBI9gBaebbnrfifHhDYfgasaacH8akY=wiFfYdH8Gipec8Eeeu0xXdbba9frFj0=OqFfea0dXdd9vqai=hGuQ8kuc9pgc9s8qqaq=dirpe0xb9q8qiLsFr0=vr0=vr0dc8meaabaqaciaacaGaaeqabaqabeGadaaakeaaieaacqWFqbaudaWgaaWcbaGaeyOhIukabeaakmaabmaabaacbeGae4hEaG3aaWbaaSqabeaacqGFMbGzaaGccqGG8baFcqGF4baEaiaawIcacaGLPaaacqGHijYUdaWcaaqaaiab=1eanjab=neadjab=veafnaaCaaaleqabaGae8NzaygaaaGcbaGae8xta0Kae83qamKae8xrau0aaSbaaSqaaiab=veafjab=fhaXjab=1gaTbqabaaaaOGaeiOla4caaa@45AD@

Here, MCE^f ^is the number of times a state **x**^**f **^is observed in an equilibrium simulation of MCE_Eqm _MC events of the fast network. We refer to the PDFs based on the life-time of states (Eq. (1)) and frequency of states (Eq. (2)) as the temporal and frequency PDF, respectively. In most cases, the frequency and temporal PDFs are practically the same. However, differences arise when the QE PDFs are multimodal and/or when low populations are encountered, as was shown in Ref. [17]. Numerical examples of this paper further indicate that the frequency PDF method can result in erroneous results in such cases, so we strongly recommend the use of the temporal PDF.

### Calculation of Slow-Scale Propensities

The propensity function of the slow reaction Rjs
 MathType@MTEF@5@5@+=feaafiart1ev1aaatCvAUfKttLearuWrP9MDH5MBPbIqV92AaeXatLxBI9gBaebbnrfifHhDYfgasaacH8akY=wiFfYdH8Gipec8Eeeu0xXdbba9frFj0=OqFfea0dXdd9vqai=hGuQ8kuc9pgc9s8qqaq=dirpe0xb9q8qiLsFr0=vr0=vr0dc8meaabaqaciaacaGaaeqabaqabeGadaaakeaaieWacqWFsbGudaqhaaWcbaGae8NAaOgabaGae83Camhaaaaa@30D2@, given by ajs
 MathType@MTEF@5@5@+=feaafiart1ev1aaatCvAUfKttLearuWrP9MDH5MBPbIqV92AaeXatLxBI9gBaebbnrfifHhDYfgasaacH8akY=wiFfYdH8Gipec8Eeeu0xXdbba9frFj0=OqFfea0dXdd9vqai=hGuQ8kuc9pgc9s8qqaq=dirpe0xb9q8qiLsFr0=vr0=vr0dc8meaabaqaciaacaGaaeqabaqabeGadaaakeaaieaacqWFHbqydaqhaaWcbaGae8NAaOgabaGae83Camhaaaaa@30ED@(**x**^**f**^,**x**^**s**^), is a function of the states of the slow and the fast species. Thus, the distribution of **X**^**f **^results in a distribution of ajs
 MathType@MTEF@5@5@+=feaafiart1ev1aaatCvAUfKttLearuWrP9MDH5MBPbIqV92AaeXatLxBI9gBaebbnrfifHhDYfgasaacH8akY=wiFfYdH8Gipec8Eeeu0xXdbba9frFj0=OqFfea0dXdd9vqai=hGuQ8kuc9pgc9s8qqaq=dirpe0xb9q8qiLsFr0=vr0=vr0dc8meaabaqaciaacaGaaeqabaqabeGadaaakeaaieaacqWFHbqydaqhaaWcbaGae8NAaOgabaGae83Camhaaaaa@30ED@(**x**^*f*^,**x**^*s*^) for all j ∈ Rjs
 MathType@MTEF@5@5@+=feaafiart1ev1aaatCvAUfKttLearuWrP9MDH5MBPbIqV92AaeXatLxBI9gBaebbnrfifHhDYfgasaacH8akY=wiFfYdH8Gipec8Eeeu0xXdbba9frFj0=OqFfea0dXdd9vqai=hGuQ8kuc9pgc9s8qqaq=dirpe0xb9q8qiLsFr0=vr0=vr0dc8meaabaqaciaacaGaaeqabaqabeGadaaakeaaieWacqWFsbGudaqhaaWcbaGae8NAaOgabaGae83Camhaaaaa@30D2@. The transition probabilities used to evolve the slow network should be projected onto the QE PDF of the fast network. In Ref. [17], we underscored that the most rigorous way of evolving the slow processes should be based on a joint PDF that accounts for the life time of various fast states along with the rate at which these states are being changed by slow processes.

Evaluating the first moment of the propensity, ajs
 MathType@MTEF@5@5@+=feaafiart1ev1aaatCvAUfKttLearuWrP9MDH5MBPbIqV92AaeXatLxBI9gBaebbnrfifHhDYfgasaacH8akY=wiFfYdH8Gipec8Eeeu0xXdbba9frFj0=OqFfea0dXdd9vqai=hGuQ8kuc9pgc9s8qqaq=dirpe0xb9q8qiLsFr0=vr0=vr0dc8meaabaqaciaacaGaaeqabaqabeGadaaakeaaieaacqWFHbqydaqhaaWcbaGae8NAaOgabaGae83Camhaaaaa@30ED@(**x**^**f**^,**x**^**s**^), over the temporal QE PDF, P_∞_(**x**^**f **^| **x**), is a simpler way to assimilate the stochastic QE description into the dynamics of the slow network [13]. The SS-SSA [13] uses an analytical expression of QE-PDF to evaluate Eq. (10), which restricts the applicability of the algorithm to simple networks. Alternatively, it uses a deterministic, steady-state solution of the fast network to evaluate the slow-scale propensities. As correctly pointed out by Cao et al. [13], this mean-field approach neglects the correlation in the stochastic QE and is accurate at large populations only. In a numerical example, we show that the mean-field approach also fails when the fast network has multiple steady states. Other stochastic multiscale methods [16,17,20], which employ the slow-scale approximation, obtain the slow-scale propensities via a numerical approximation, and are, therefore, more general than the SS-SSA, at the expense, of course, of increased computational cost.

Here, we follow Ref. [20] and numerically compute the average propensities instead of the entire PDF. However, we ensure that the state we pick to fire from can indeed be fired, i.e., no negative populations arise (see also main text). This approach effectively accounts for the joint PDF approach of Ref. [17]. More specifically, the slow-scale propensity of each slow reaction can be evaluated as

ajs¯(x)=∑xf′Δtf′⋅ajs(xf′,xs)τEqm, for j=1,2,...,ms
 MathType@MTEF@5@5@+=feaafiart1ev1aaatCvAUfKttLearuWrP9MDH5MBPbIqV92AaeXatLxBI9gBaebbnrfifHhDYfgasaacH8akY=wiFfYdH8Gipec8Eeeu0xXdbba9frFj0=OqFfea0dXdd9vqai=hGuQ8kuc9pgc9s8qqaq=dirpe0xb9q8qiLsFr0=vr0=vr0dc8meaabaqaciaacaGaaeqabaqabeGadaaakeaadaqdaaqaaGqaaiab=fgaHnaaDaaaleaacqWFQbGAaeaacqWFZbWCaaaaaGqabOGae4hkaGIae4hEaGNae4xkaKIaeyypa0ZaaSaaaeaadaaeqbqaaiabgs5aejab=rha0naaCaaaleqabaGaf8NzayMbauaaaaGccqGHflY1cqWFHbqydaqhaaWcbaGae8NAaOgabaGae83CamhaaOWaaeWaaeaacqGF4baEdaahaaWcbeqaaiqb+zgaMzaafaaaaOGaeiilaWIae4hEaG3aaWbaaSqabeaacqGFZbWCaaaakiaawIcacaGLPaaaaSqaaiab=Hha4naaCaaameqabaGaf8NzayMbauaaaaaaleqaniabggHiLdaakeaaiiGacqqFepaDdaWgaaWcbaGae8xrauKae8xCaeNae8xBa0gabeaaaaGccqGGSaalcqqGGaaicqWFMbGzcqWFVbWBcqWFYbGCcqqGGaaicqWFQbGAcqGH9aqpcqaIXaqmcqGGSaalcqaIYaGmcqGGSaalcqGGUaGlcqGGUaGlcqGGUaGlcqGGSaalcqWFTbqBdaahaaWcbeqaaiab=nhaZbaaaaa@66DB@

Eq. (3) can be rewritten as follows

ajs¯(x)=∑p=1MCEEqmf(τf⋅ajs)|pτEqm, for j=1,2,...,ms
 MathType@MTEF@5@5@+=feaafiart1ev1aaatCvAUfKttLearuWrP9MDH5MBPbIqV92AaeXatLxBI9gBaebbnrfifHhDYfgasaacH8akY=wiFfYdH8Gipec8Eeeu0xXdbba9frFj0=OqFfea0dXdd9vqai=hGuQ8kuc9pgc9s8qqaq=dirpe0xb9q8qiLsFr0=vr0=vr0dc8meaabaqaciaacaGaaeqabaqabeGadaaakeaadaqdaaqaaGqaaiab=fgaHnaaDaaaleaacqWFQbGAaeaacqWFZbWCaaaaaGqabOGae4hkaGIae4hEaGNae4xkaKcccaGae0xpa0ZaaSaaaeaadaaeWbqaamaaeiaabaWaaeWaaeaaiiGacqaFepaDdaahaaWcbeqaaiab=zgaMbaakiabgwSixlab=fgaHnaaDaaaleaacqWFQbGAaeaacqWFZbWCaaaakiaawIcacaGLPaaaaiaawIa7amaaBaaaleaacqWFWbaCaeqaaaqaaiab=bhaWjabg2da9iabigdaXaqaaiab=1eanjab=neadjab=veafnaaDaaameaacqWFfbqrcqWFXbqCcqWFTbqBaeaacqWFMbGzaaaaniabggHiLdaakeaacqaFepaDdaWgaaWcbaGae8xrauKae8xCaeNae8xBa0gabeaaaaGccqGGSaalcqqGGaaicqWFMbGzcqWFVbWBcqWFYbGCcqqGGaaicqWFQbGAcqGH9aqpcqaIXaqmcqGGSaalcqaIYaGmcqGGSaalcqGGUaGlcqGGUaGlcqGGUaGlcqGGSaalcqWFTbqBdaahaaWcbeqaaiab=nhaZbaaaaa@6AD4@

where MCEEqmf
 MathType@MTEF@5@5@+=feaafiart1ev1aaatCvAUfKttLearuWrP9MDH5MBPbIqV92AaeXatLxBI9gBaebbnrfifHhDYfgasaacH8akY=wiFfYdH8Gipec8Eeeu0xXdbba9frFj0=OqFfea0dXdd9vqai=hGuQ8kuc9pgc9s8qqaq=dirpe0xb9q8qiLsFr0=vr0=vr0dc8meaabaqaciaacaGaaeqabaqabeGadaaakeaaieaacqWFnbqtcqWFdbWqcqWFfbqrdaqhaaWcbaGae8xrauKae8xCaeNae8xBa0gabaGae8Nzaygaaaaa@3541@ is the number of MC events executed by the *microscopic solver *to simulate time (after relaxation has been achieved) τEqm=∑p=1MCEEqmfτpf
 MathType@MTEF@5@5@+=feaafiart1ev1aaatCvAUfKttLearuWrP9MDH5MBPbIqV92AaeXatLxBI9gBaebbnrfifHhDYfgasaacH8akY=wiFfYdH8Gipec8Eeeu0xXdbba9frFj0=OqFfea0dXdd9vqai=hGuQ8kuc9pgc9s8qqaq=dirpe0xb9q8qiLsFr0=vr0=vr0dc8meaabaqaciaacaGaaeqabaqabeGadaaakeaaiiGacqWFepaDdaWgaaWcbaacbaGae4xrauKae4xCaeNae4xBa0gabeaakiabg2da9maaqahabaGae8hXdq3aa0baaSqaaiab+bhaWbqaaiab+zgaMbaaaeaacqGFWbaCcqGH9aqpcqaIXaqmaeaacqGFnbqtcqGFdbWqcqGFfbqrdaqhaaadbaGae4xrauKae4xCaeNae4xBa0gabaGae4NzaygaaaqdcqGHris5aaaa@4647@ of the quasi- equilibrated fast network. τf|p
 MathType@MTEF@5@5@+=feaafiart1ev1aaatCvAUfKttLearuWrP9MDH5MBPbIqV92AaeXatLxBI9gBaebbnrfifHhDYfgasaacH8akY=wiFfYdH8Gipec8Eeeu0xXdbba9frFj0=OqFfea0dXdd9vqai=hGuQ8kuc9pgc9s8qqaq=dirpe0xb9q8qiLsFr0=vr0=vr0dc8meaabaqaciaacaGaaeqabaqabeGadaaakeaadaabcaqaaGGaciab=r8a0naaCaaaleqabaacbaGae4NzaygaaaGccaGLiWoadaWgaaWcbaGae4hCaahabeaaaaa@332E@ is the time increment in the p^th ^MC event, and ajs|p
 MathType@MTEF@5@5@+=feaafiart1ev1aaatCvAUfKttLearuWrP9MDH5MBPbIqV92AaeXatLxBI9gBaebbnrfifHhDYfgasaacH8akY=wiFfYdH8Gipec8Eeeu0xXdbba9frFj0=OqFfea0dXdd9vqai=hGuQ8kuc9pgc9s8qqaq=dirpe0xb9q8qiLsFr0=vr0=vr0dc8meaabaqaciaacaGaaeqabaqabeGadaaakeaadaabcaqaaGqaaiab=fgaHnaaDaaaleaacqWFQbGAaeaacqWFZbWCaaaakiaawIa7amaaBaaaleaacqWFWbaCaeqaaaaa@341E@ is the propensity of the j^th ^slow reaction before the occurrence of the p^th ^MC event.
